# A Redox‐Active Heterobimetallic N‐Heterocyclic Carbene Based on a Bis(imino)pyrazine Ligand Scaffold

**DOI:** 10.1002/anie.202005865

**Published:** 2020-08-28

**Authors:** Nicolas I. Regenauer, Sven Jänner, Hubert Wadepohl, Dragoş‐Adrian Roşca

**Affiliations:** ^1^ Anorganisch-Chemisches Institut Universität Heidelberg Im Neuenheimer Feld 276 69120 Heidelberg Germany

**Keywords:** carbene ligands, heterobimetallic complexes, heterocycles, redox-active ligands, redox chemistry

## Abstract

A new redox‐active N‐heterocyclic carbene (NHC) architecture is obtained using N‐methylated pyrazinediimine iron complexes as precursors. The new species exhibit strong π‐accepting/σ‐donating properties and are able to ligate two metal centres simultaneously. The redox activity was demonstrated by the reversible chemical oxidation of a heterobimetallic Fe^0^/Rh^I^ example, which affords an isolable ligand‐based radical cation. The reversible redox process was then applied in the catalytic hydrosilylation of 4,4′‐difluorobenzophenone, where the reaction rate could be reversibly controlled as a function of the catalyst oxidation state. The new NHC exhibits high electrophilicity and nucleophilicity, which was demonstrated in the reversible activation of alcohols and amines. The electronic structure of the resulting complexes was investigated through various spectroscopic and computational methods.

## Graphical abstract

Symbiosis: A novel redox‐switchable N‐heterocyclic carbene architecture based on pyrazine‐diimine systems is presented. We demonstrate its ambiphilicity in the reversible activation of alcohols and amines. This scaffold is capable of ligating two metals simultaneously, and the resulting heterobimetallic complexes can be reversibly oxidised and reduced.

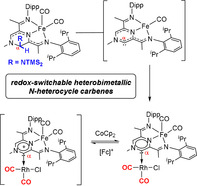

## Introduction

N‐heterocyclic carbenes (NHCs) have had a significant impact on the field of organometallic chemistry and of homogenous catalysis, where they have become widespread ligands for a myriad of transformations. Key to their success was the ability to accommodate a large number of transition metals, as well as facile tunability of steric bulk and electronic σ‐donating/π‐accepting properties. The parametrisation of these properties through the buried volume (%V_bur_) or the Tolman Electronic Parameter (TEP) has provided useful tools for the design of powerful new catalytic systems,^1^ allowing chemists to choose the most suitable candidates from a plethora of possibilities.^2^ In the instances where a facile and significant change in electronic properties is desired, whilst keeping the steric environment unaltered, a convenient strategy is the installation of a redox switch. Redox activity is typically achieved through an organic (e.g. naphthoquinone)^3^ or organometallic (most commonly sandwich‐type structures, for example, ferrocene)^4^ redox‐active fragment. In the latter case, the Fe^II^/Fe^III^ reversible couple enables the modulation of the NHC electronic properties upon reversible oxidation of the ferrocene backbone,^5^ which in turn expands their catalytic scope, in comparison to classical NHCs.^6^ Moreover, their ambiphilicity makes them excellent tools for small molecule activation,^7^ and enables them to ligate a variety of metals, expanding therefore the tool‐box available for redox‐switch catalysis.^4^, ^5^, ^6^ Nevertheless, the prevalence of ferrocene as a redox‐switch narrows the potential‐window needed to be applied for the redox chemistry to occur, making them largely dependent on those of the Fc^0^/Fc^+^ (Fc=ferrocene) couple. Herein, we wish to introduce a new, non‐ ferrocene based redox‐active carbene architecture, which makes use of a Fe^0^‐ligated pyrazinediimine ligand (P^Pz^DI), where both the iron centre and the ligand framework can be involved in the redox activity.^8^ We envisaged that the new ligand scaffold would offer the following advantages: *(a)* as the formally Fe^0^ centre in PDI/P^Pz^DI‐type ligands (PDI=pyridinediimine, P^Pz^DI=pyrazinediimine) is more easily oxidised than the Fe^II^ centre in ferrocene, milder oxidation conditions would allow access to the oxidised form^9^ *(b)* while in ferrocene, the iron centre is more reluctant to engage in reactivity, iron‐PDI complexes display very rich chemistry ranging from catalysis to small molecule activation, making them the systems of choice for a considerable number of transformations^10^ *(c)* the P^Pz^DI‐ligand is itself redox active through the reversible reduction of the imine functionality or ligand core, therefore allowing access to more redox states. Herein, we wish to communicate the synthesis of the new iron‐P^Pz^DI NHC‐like precursors, the redox chemistry of the corresponding rhodium complexes and examples of reversible alcohol and amine activation at the in situ generated carbene centre.

## Results and Discussion

**Scheme 1:**
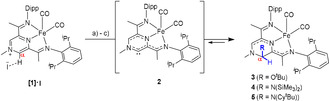
Conditions: a) for **3**: KO^t^Bu, [D_6_]benzene or [D_8_]THF, 20 min, quant. (NMR); b) for **4**: KN(SiMe_3_)_2_, [D_6_]benzene or [D_8_]THF, 15 min, 40–65 % (NMR) c) for **5**: Li(OEt)_2_(NCy^t^Bu), [D_8_]THF, 15 min, 40 % (NMR).

Deprotonation of the iron‐based methylpyrazinium complex **1⋅[I]** in the presence of a weakly nucleophilic base such as KO^t^Bu is accompanied by a rapid colour change from brown to purple. The NMR spectroscopic data suggest the loss of the *C*_2*v*_ symmetry in solution, and the formation of adduct **3** through a formal nucleophilic attack on the α‐C (Scheme 1). Increasing the steric bulk of the base, by employing KN(SiMe_3_)_2_ and even Li(OEt)_2_NCy^t^Bu^11^ does not prevent the addition of the poor nucleophiles even when the reaction is conducted at −40 °C, and the corresponding adducts **4** and **5** could be observed by ^1^H NMR spectroscopy in [D_6_]benzene or [D_8_]THF solutions, suggesting also that the pyrazine core is dearomatised (vide infra). While stable in solution for at least 24 h, complexes, **3**–**5** cannot be isolated as solids: removal of the respective solvent under vacuum, followed by re‐dissolving the reaction mixture in the same NMR solvent shows a complex mixture of species. The reactivity pattern suggests that the addition of the weakly nucleophilic base is reversible, and points towards an unstable NHC **2** intermediate, generated upon subjecting the reaction mixture to high vacuum.^12^ Formal reductive elimination from NHC derivatives on steric grounds is documented in the literature, and in the case of **3**–**5** we assume that the steric bulk of the added base is the driving force for regenerating the free carbene.^13^ We would like to point out, that while analysing the degradation of **2**, we could not observe any evidence for a Wanzlick‐type dimerization; the resulting reaction mixture likely contains paramagnetic species as judged by the broad signals observed by ^1^H NMR spectroscopy.

**Scheme 2:**
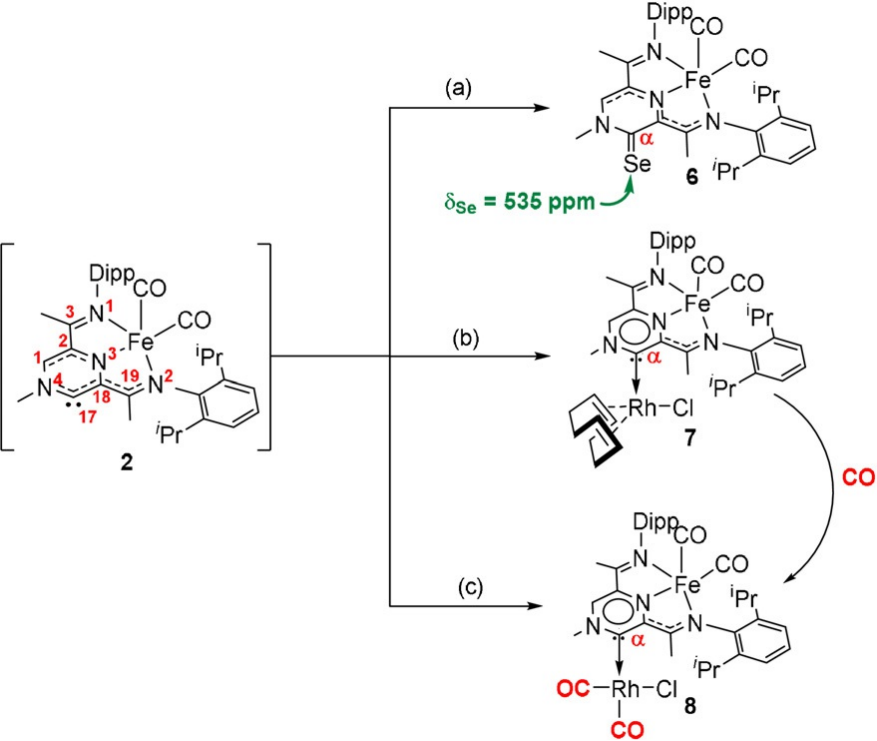
NHC **2** was generated in situ as described in Scheme 1 and its yield was assumed 35 %. Conditions: a) Se (2 equiv), THF, 1 h, quant. (NMR) b) [Rh(COD)Cl]_2_ (0.5 equiv), THF, 2 h, 90 % (isolated). c) [Rh(CO)_2_Cl]_2_ (0.5 equiv), THF, 2 h, 96 % (isolated).

The reactivity of **2** with very poor nucleophiles testifies to the strong electrophilic character of the transient carbene species. In order to further assess this characteristic experimentally, **4** was treated with elemental selenium, which allowed the clean conversion to the selenourea derivative **6** (Scheme 2). Interestingly, even though **4** bears a formally Fe^0^ centre, no oxidation of the metal was observed, and the resulting complex **6** exhibits well‐resolved NMR resonances, typical for a diamagnetic compound. Selenium NMR chemical shifts of selenoureas are an established method to measure the π‐acidity of NHCs.^1^, ^14^ In this respect, a measured ^77^Se NMR signal located at 535 ppm, places **2** significantly downfield compared to the established IPr (*δ*_Se_=87 ppm) and SIPr congeners (*δ*_Se_=181 ppm),^1^ and between Bertrand's 5‐memberd ring cyclic(alkyl)(amino)carbenes (5‐cAAC) (*δ*_Se_=492 ppm) and bicyclic(alkyl)(amino)carbenes BICAAC (*δ*_Se_=645 ppm),^15^ a characteristic that corroborates well with the high electrophilicity observed experimentally.^16^

To further explore the electronic properties of **2**, we have synthesised the corresponding Rh complexes. As described previously, the hexamethyldisilazane (HMDS) adduct proved to be a good precursor for **2**, and reacting a freshly prepared solution of **4** with either [Rh(COD)Cl]_2_ or [Rh(CO)_2_Cl]_2_ readily afforded the heterobimetallic Fe/Rh complexes **7** and **8** (Scheme 2).^17^ The Rh‐carbonyl complex **8** could also be generated by placing **7** under one atmosphere of carbon monoxide. The facile COD displacement testifies to the strong *trans*‐effect exerted by the carbene ligand towards the Rh−COD bond, reducing the energetic barrier for the ligand exchange reaction. Both **7** and **8** display well‐resolved NMR spectra, suggesting that the closed‐shell singlet state of the formally Fe^0^ centre is not changed by the introduction of a second metal such as rhodium. Particularly informative is the ^13^C NMR chemical shift of the rhodium ligated carbene C‐atom, which exhibits a characteristic low field doublet (in **7**: *δ*_C_=204.4 ppm, ^1^ *J*_RhC_=42 Hz; in **8**: *δ*_C_=185.5 ppm, ^1^ *J*_RhC_=37.5 Hz). ^15^N NMR chemical shifts for **6**–**8** suggest that the pyrazine ring retains aromaticity (*δ*_N_ (NMe)=170.6 ppm in **6**, 176.1 in **7**, 171.4 in **8**, similar to 145.5 in **[1]⋅I**).^18^ The average value of the Rh‐carbonyl stretching frequencies (ν^av^_Rh‐CO_) is generally used to measure the overall donor capabilities of NHCs. Dichloromethane solutions of compound **8** exhibit three main CO stretching frequencies, as a result of the overlapping between the Fe‐CO and Rh‐CO vibrational modes.^1^, ^2^ Based on comparison with our previous compounds and DFT calculations (vide infra), we could assign the stretching frequencies located at 2074 and 1994 cm^−1^ to the Rh‐CO fragments (ν^av^_Rh‐CO_=2034 cm^−1^, TEP=2047 cm^−1^). The average value suggests that despite the high electrophilicity, **2** has high electron donating capacity—higher than the standard imidazolin‐2‐ylidene and imidazolidin‐2‐ylidene derivatives, but lower than certain cyclic alkyl amino carbenes (cAACs). Nevertheless, taken together, the spectroscopic data suggest that **2** has an ambiphilic character.^1^, ^19^ The ambiphilicity can be rationalised in the unique combination of a pyrazine ring and a Fe(CO)_2_ fragment. While the diazine ring is π‐acidic, the iron‐ligated nitrogen atom is unable to stabilise the carbene formally empty p_π_ orbital as it is already engaged in interaction with the iron centre (vide infra). To the best of our knowledge, using a pyrazine ring as a scaffold for the generation of NHCs to give well‐defined metal complexes is so far unreported.^20^

**Figure 1:**
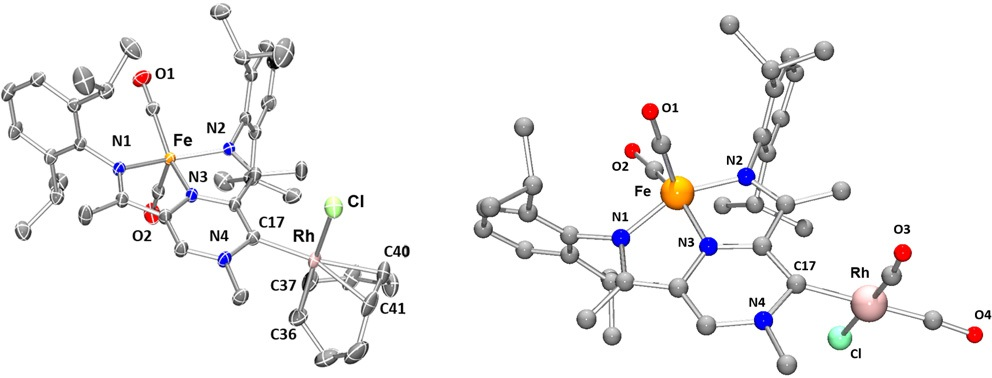
Molecular structure obtained by single crystal X‐ray diffraction of **7** (left) and **8** (right). Hydrogen atoms are omitted for clarity.

The molecular structures of **7** and **8** could be determined by single crystal X‐ray diffraction (Figure 1).^21^ The molecular structure of **7** reveals that the pyrazine ring retains its aromaticity^22^ and is essentially flat. The N‐Cα‐C angle is nevertheless compressed (∡114.29(14)° in **7**, compared to 119.03(18) in **1⋅[I]** 8). The magnitude of the angle compression is similar to the one in the analogous isoquinolin‐1‐ylidine rhodium(I) complexes.^23^ The diffraction data quality obtained for **8** precluded us from discussing the metric parameters; however, the connectivity could be established unambiguously.

In order to probe the redox‐switchability of the isolated heterobimetallic complexes, we have initially performed cyclic voltammetry of **7** and **8**. Both complexes exhibit one quasi‐reversible redox wave at very similar peak potentials (−0.6 V in **7**, and −0.54 V in **8**, vs. Fc/Fc^+^), which we assign to the reversible redox chemistry of the formally Fe^0^ centre. This is followed by two irreversible oxidation waves, which we tentatively assign to the oxidation of the rhodium centre.^24^ Interestingly, unlike the diaminocarbene[3]ferrocenophanes described by Bielawski and Siemeling,5a, 5b where a switch from COD to CO on the rhodium centre had a marked influence on the ferrocene Fe^II^/Fe^III^ redox potentials (of ca. 370 mV), the same variation of substituents appears to have negligible influence on the redox potentials of the Fe^0^/Fe^I^ couple. The marked cathodic shift in diaminocarbene[3]ferrocenophane is explained by *through space* Fe⋅⋅⋅Rh interactions, which would be absent in **7** and **8**. Nevertheless, the attenuated shift in **7** and **8** could also be explained by CO/COD ligand scrambling between the two metal centres in **7**, which is facilitated by the labilisation of the Fe−CO bond as a result of the oxidation of the iron centre, effectively reducing the amount of backbonding interactions.

**Scheme 3:**
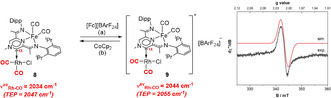
Conditions: a) [Fc][BArF_24_] (1 equiv), CH_2_Cl_2_, 1 h, RT, 74 % (isolated); b) CoCp_2_ (1 equiv), CH_2_Cl_2_, 1 h, RT, quant. (NMR) (Fc=Ferrocenium, BArF_24_=B(3,5‐CF_3_‐C_6_H_3_)_4_) (right). Experimental (black) and simulated (red) X‐band CW‐EPR spectrum recorded at 6 K for **9** (left).

In order to verify the reversibility observed by cyclic voltammetry, we have treated **8** with a ferrocenium salt ([Fc][BArF_24_]) which gave rise to a paramagnetic compound **9** (Scheme 3) displaying a magnetic susceptibility characteristic for one unpaired electron (*μ*_eff_=1.91 *μ*_B_). In contrast to the diaminocarbene[3]ferrocenophanes system described by Bielawski,5a the oxidised **9** product is stable enough to be isolated, even though we could not obtain X‐ray quality crystals despite numerous attempts. Dichloromethane solutions of **9** display three main IR stretching frequencies, as a result of the overlapping between the Fe‐CO (2007 and 1948 cm^−1^) and Rh‐CO (2081 and 2007 cm^−1^) vibrational modes. These values are shifted to higher frequencies compared to the parent compound **8**, in line with the reduced amount of backbonding expected for **9**. Overall, the IR data suggest a significant alteration of the electronic properties of **9** (ν^av^_Rh‐CO_=2044 cm^−1^, TEP=2055 cm^−1^) compared to **8** (ν^av^_Rh‐CO_=2034 cm^−1^, TEP=2047 cm^−1^), with the overall donating properties of **9** being similar to the established IMes and IPr NHCs. This variation in the TEP is comparable to the values reported for other redox‐switchable NHCs.^4^ Additionally, we could prove that this chemical oxidation is fully reversible: treatment of **9** with one equivalent of cobaltocene cleanly regenerates **8** alongside [CoCp_2_][BArF_24_] (Scheme 3).

As the formal oxidation of the iron centre has a marked effect on the stretching frequencies of the Rh‐CO unit, we wondered whether the resulting unpaired electron resides on the iron centre, or it is delocalised over the entire conjugated system. As a Fe (or Rh) based radical would have a specific EPR signature compared to an organic radical, **9** was investigated by X‐band CW‐EPR spectroscopy. The data, recorded at 6 K (Scheme 3, right), reveals a pattern characteristic for a ligand centred radical (S=1/2 ), with no distinguishable hyperfine structure and with very small g anisotropy, centred at *g=*2.0080 (*g*_e_=2.0023). These data suggest reduced spin density on the iron centre and are consistent with significant spin delocalisation. A signal possessing the same characteristics could also be observed at room temperature in THF solutions, albeit with reduced intensity (see the supporting information).

**Figure 2:**
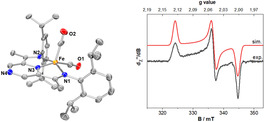
Characterisation data for **10**: Molecular structure obtained by single crystal X‐ray diffraction. The BArF_24_ anion and hydrogen atoms are omitted for clarity (left). (b) Experimental (black) and simulated (red) X‐band CW‐EPR spectrum recorded at 6 K (right).

**Scheme 4:**
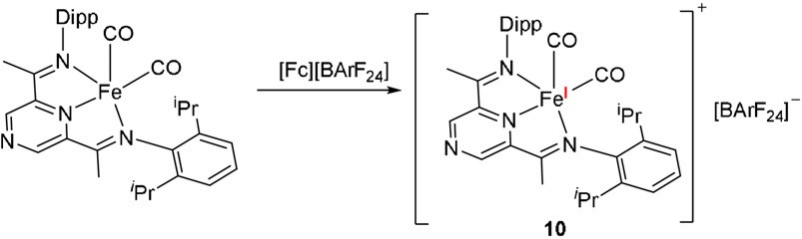
Conditions: [Fc][BArF_24_] (1 equiv), C_6_H_6_, 1 h, RT, 78 % (isolated).

In order to ascertain whether the radical delocalisation over the entire ligand scaffold is a result of the carbene functionality, we have prepared [(P^Pz^DI)Fe(CO)_2_][BArF_24_] **10** (Scheme 4) in an analogous fashion, by oxidising (P^Pz^DI)Fe(CO)_2_ in the presence of [Fc][BArF_24_] (Figure 2, left). In contrast to **9**, the X‐band CW‐EPR spectrum of **10** recorded at 6 K displays a rhombic signal (Figure 2, right), and the fit of the data yielded the following *g* values: *g*_min_=1.997, *g*_mid_=2.044, *g*_max_=2.124, consistent with a S=1/2 compound. The g anisotropy indicates that the singly occupied molecular orbital (SOMO) is iron‐based rather than ligand‐based. The characteristics of **10** are similar to the pyridine‐based Fe^I^ analogue, [(PDI)Fe(CO)_2_][BArF_24_] reported by Chirik.^9^

### Catalytic Redox Switchability

**Scheme 5:**
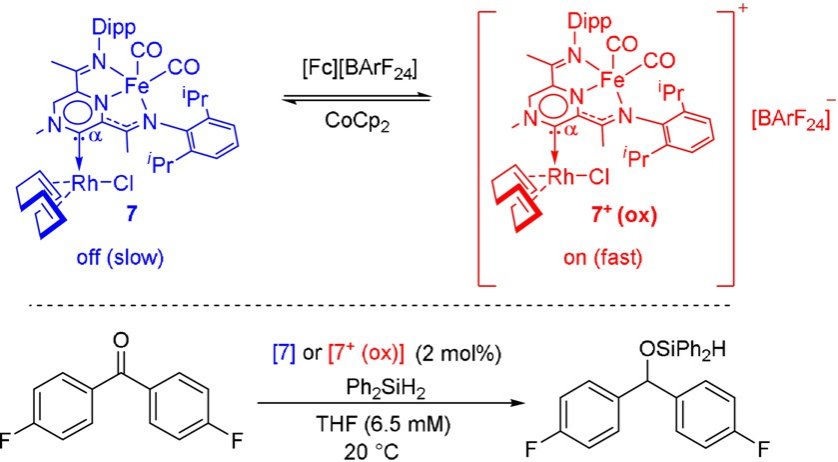
Hydrosilylation of 4,4′‐difluorobenzophenone catalysed by the reduced or oxidised form of **7**.

We have shown that the oxidation of **8** significantly alters the electronic properties of the resulting species, as demonstrated by IR and EPR spectroscopy. As the oxidised species **9** is stable and isolable while undergoing reduction cleanly to regenerate compound **8** (Scheme 3), we wondered whether we could employ the reversible change in electronic properties in redox‐switch catalysis. For demonstrating the change in reactivity between the reduced and oxidised forms, we have investigated the hydrosilylation reaction of 4,4′‐difluorobenzophenone in the presence of Ph_2_SiH_2_. The choice of substrate was made in order to facilitate reaction monitoring by NMR (^19^F) and IR (C=O stretching of the ketone, Si‐H wagging mode of the silane) spectroscopy. In order to minimise the induction time, the more labile COD substituted [FeRh] pre‐catalyst **7** was chosen. Initial catalytic runs were performed in the presence of neutral (**7**) and the in situ‐oxidised analogue (**7^+^**) respectively (Scheme 5). Under the same reaction conditions, while full conversion of 4,4‐difluorobenzophenone in the presence of **7** was observed after 12 hours, a significant acceleration of the reaction rate was observed when **7^+^** was used as a catalyst, with full conversion after 2.5 hours being noted by NMR and IR spectroscopy. A comparison of the reaction rate constants extracted through the initial rates method indicates that the reaction catalysed by the oxidised species is one order of magnitude faster.^25^ As we envisage that a rhodium hydride is the catalytically active species, reduced electron density on the rhodium centre would enhance the hydridic character and facilitate the subsequent insertion step.^26^, ^27^

Taking the significant difference in the reaction rate between the oxidised and reduced forms into account, we sought to demonstrate that the change between the two kinetic regimes was possible through in situ oxidation and reduction. Conducting the catalytic hydrosilylation reaction in a cell fitted with an IR probe allowed us to detect rapid changes in the kinetic profile as a function of external stimuli. Sequential addition of [Fc][BArF_24_] and CoCp_2_ successfully alters the reaction rate by one order of magnitude. In line with the stability of both the neutral (**7**) and oxidised species (**7^+^**), the temporal control was depicted through three “on/off” cycles, which could be performed without apparent loss of catalytic activity.^25^, ^28^ These results demonstrate that PDI‐ligands can be used as redox switches in NHC chemistry, and therefore join the family of more established redox‐switches such as metallocenes and quinones.^29^

**Figure 3:**
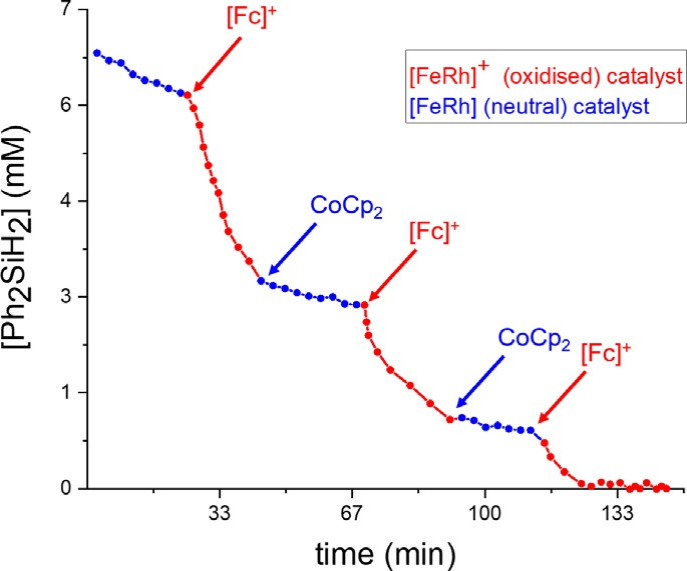
Plot of Ph_2_SiH_2_ consumption over time for the hydrosilylation of 4,4′‐difluorobenzophenone during the in situ oxidation and reduction of complex **7/7^+^** illustrating temporal control. Kinetic profile determined by ReactIR measurements. Measurements were performed every 15 seconds. For clarity, only every 10^th^ data point is displayed.

### Reactivity at the Carbene Centre

**Scheme 6:**
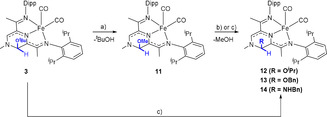
a) MeOH (45 equiv), C_6_H_6_, 10 min, 89 % (isolated) b) NMR experiments: [D_6_]benzene, 10 min—for R=OBn (**13**): BnOH (3 equiv), 75 % (NMR); for R=NHBn (**14**), (2 equiv), 66 % (NMR) or c) Larger scale experiments: R=O^i^Pr (**12**): iPrOH (18 equiv), C_6_H_6_, 5 min, 95 % (isolated); for R=OBn (**13**): BnOH (3 equiv), C_6_H_6_, 30 min, 40 % (isolated).

As detailed above, reacting **1**⋅**[I]** with bulky alkoxides and amides readily affords the corresponding alcohol and amine adducts **3**–**5**, which act as carbene precursors, but cannot be isolated as solids due to the kinetic lability of the bases employed. In order to get more insight in the structure and reactivity of carbene **2**, the structure and reactivity of these adducts would provide valuable information. We therefore envisaged that replacing the bulky amine or alcohol fragments with OMe would increase the stability of the resulting adducts. Reacting derivative **3** with methanol affords indeed the OMe substituted derivative **11** in quantitative yield, alongside ^*t*^BuOH as observed by ^1^H NMR spectroscopy (Scheme 6).

**Figure 4:**
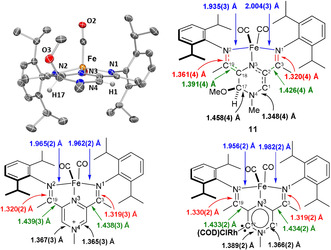
(top) Molecular structure of **11** obtained by single crystal X‐ray diffraction and selected metric data. (bottom) Selected metric data for **[1]⋅[I]** ^8^ and **7** obtained from single crystal X‐ray diffraction.

Unlike **3**–**5**, **11** could be isolated as a solid and recrystallised from pentane and its structure could be determined by single crystal X‐ray analysis (Figure 4, top). NMR data are consistent with a formal MeOH addition to Cα, which induces dearomatisation of the pyrazine ring, evident in the upfield shift of the *δ*_N_ (NMe) to 94.8, when compared to the 145.5 value measured for **1⋅[I]**. The imine character of the C_19_=N_2_ bond adjacent to the MeOH is also diminished, as shown by the significant upfield shift ^15^N NMR signal (*δ*_N2_=188.2 ppm vs. *δ*_N1_=231.4 ppm in **1**⋅**[I]**) (Numbering scheme, Figure 4). These NMR characteristics are also observed for **3**–**5** (see the supporting information). The decrease of electron density on the imine arm revealed by ^15^N NMR spectroscopy is also reflected in the elongation of the C^19^=N^2^ bond to 1.361(4) Å compared to the average value of 1.325 Å observed for **1⋅[I]** and **7** (Figure 4). Furthermore, the ∡C^18^‐C^17^‐N^4^ (110.6(3)°) is significantly compressed compared to ∡C^2^‐C^1^‐N^4^ (122.0(3)°) suggesting a tetrahedral geometry at C^17^, in line with a change of hybridisation from sp^2^ in **1⋅[I]** to sp^3^, as a result of formal methanol addition.

The CO stretches determined by solid state IR spectroscopy are also responsive to the alteration of the electronic environment and shift to lower frequencies (1949, 1882 cm^−1^ in **11**), as expected for a more electron rich system, which enhances backbonding from Fe to the carbonyl ligands.

While **11** is thermodynamically stable, it degrades rapidly in [D_2_]dichloromethane solutions, generating **1⋅[Cl]** alongside unidentified paramagnetic impurities.^30^ This reactivity is also observed for compounds **3**–**5**. In contrast, as expected, compounds **6**–**10** are stable in [D_2_]dichloromethane solutions for long periods of time.

The unconventional way of synthesising **11**, through a formal alkoxide exchange from **3** in the presence of methanol has prompted us to investigate whether this exchange reaction is general. Treating **3**, **4** or **11** with isopropanol or benzylic alcohol affords the corresponding alcohol exchange products **12** and **13** (Scheme 6). Similarly, reaction between **11** and benzylamine afforded the corresponding amine derivative **14**, with the liberation of methanol. Even upon using an excess (2–3 equiv) of the corresponding alcohols or amines, the conversion to **13** and **14** was around 75 % and 66 % respectively, suggesting an equilibrium reaction.^31^ Full conversion in the case of **12** and **13** could however be achieved when starting directly form **3**. Methoxide and isopropoxide derivatives **11** and **12** are stable and could be isolated as solids, whereas benzylic alcohol and amine derivatives **13** and **14** show clear signs of decomposition upon solvent removal. While the fate of the iron species could not be hitherto elucidated, in the case of the benzylic alcohol adduct **13**, benzaldehyde formation could be observed by ^1^H NMR spectroscopy and GC‐MS. The formation of benzaldehyde could be explained by a 1,3‐hydride shift followed by α‐elimination.^32^

### Computational Chemistry

**Figure 5:**
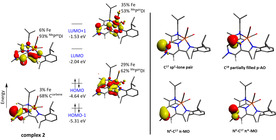
Qualitative molecular orbital diagram of **2**. Canonical molecular orbitals are displayed (left). Representative NBOs describing the bonding situation (right).

To get more insight in the electronic structure of the carbene **2** and its rhodium complexes, we have performed DFT calculations at a B3LYP/def2‐TZVP(‐f) level of theory.^33^ For complexes **1**, **7** and **10**, geometry optimisations produced features which were in good agreement with the metric data from X‐ray crystallography, as well as vibrational data from IR spectroscopy (See the supporting information for details). Potential ligand redox non‐innocence for complexes **2**, **7**, **8** and **11** was investigated through broken‐symmetry (BS) calculations, where all strategies employed converged to the same BS (0,0) (i.e. closed‐shell) solution, similar to the one observed for the starting material **1⋅[I]**, as well as (P^Pz^DI)Fe(CO)_2_.^8^ The data therefore suggest a Fe^0^ metal centre supported by a neutral ligand, in line with the diamagnetism observed for the investigated complexes. We have then proceeded by examining the frontier molecular orbitals (MOs) (Figure 5). In the case of the free carbene **2**, the HOMO and the LUMO are both energetically and morphologically similar to the ones calculated for (P^Pz^DI)Fe(CO)_2_, with a narrow HOMO–LUMO separation (2.60 eV), most likely due to the extended π‐conjugation over the pyrazine ring and imine arms. Interestingly, the carbene based *E*_σ_ and *E*_π*_ are the HOMO−1 (stabilised by 0.68 eV compared to the HOMO) and LUMO+1 (destabilised by 0.50 eV compared to the LUMO). This distribution of molecular orbitals is similar to the one observed for the bisoxazoline‐based NHC IBioxMe_4_,^34^ dipyrido‐annelated NHCs (dipiy),^35^ and the recently reported 1,3‐di(amino)oxyallyl pyrimidine‐based NHC, ^[36]^ where, in some cases, the *E*_σ_ (HOMO−1) is stabilised by ca. 0.50 eV compared to the HOMO. In the case of Siemeling's neopentyl‐substituted diaminocarbene[3]ferrocenophane, the carbene *E*_σ_ is in the HOMO (a typical situation for the vast majority of NHCs), whereas the carbene *E*_π*_ is the LUMO+2, while the LUMO and the LUMO+1 are ferrocene‐based (see the supporting information for a full MO diagram).^37^ The calculated singlet‐triplet gap (Δ*E*_ST_) for **2** appears significantly narrow (37.3 kcal mol^−1^), however, the magnitude is not directly comparable with the other reported NHCs, given the fact that in **2**, the carbene‐based orbitals are not the frontier MOs. A natural bond orbital (NBO) analysis^38^ reveals that the σ‐symmetry carbene lone pair (42 % s, 58 % p character) is only weakly stabilised via hyperconjugation into the N^4^‐C^1^ and N^3^‐C^18^ σ* MOs (see Scheme 2 for numbering), in line with the strong σ‐donating properties measured experimentally. Atoms N^4^‐C^17^‐C^18^ form a 3c/4e bond, where, in the “best” Lewis structure, a partially occupied p‐orbital located at C^18^ interacts with the strongly polarised π‐orbital of N^4^‐C^17^ (83 % N, 17 % C). The p‐type orbital located at C^18^ is also strongly delocalised into the adjacent imine bond C^19^‐N^2^, as well as the pyrazine N^4^‐C^17^ π* orbitals (see supporting information for details). These data suggest that the carbene π‐symmetry orbital is strongly delocalised over the entire diiminopyrazine ligand, and this stabilization is in line with the strong π‐accepting properties determined experimentally. The bonding picture strongly resembles the one in the 1,3‐imidazol‐4‐ylidenes, which belong to the category of mesoionic N‐heterocyclic carbenes (MICs).^39^ Nevertheless, the extent of delocalization in MICs is reduced compared to **2**, and therefore a π* molecular orbital that satisfies the symmetry criteria in order to accept electron density from a metal centre is too high in energy.^40^ Consequently, MICs are quite poor π‐acceptors, whereas in the case of **2**, a molecular orbital of appropriate symmetry is low in energy, accounting for the good π‐accepting properties (see Figure 5, LUMO+1).

**Figure 6:**
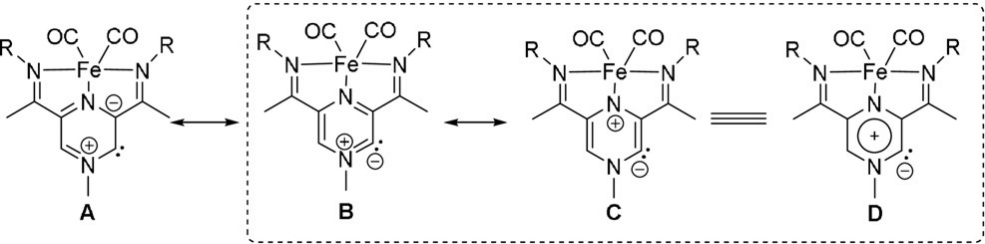
Possible resonance structures for **2**. The structures compatible with our NBO analysis are highlighted.

In line with the MIC‐like character of **2**, four resonance structures can be envisaged (Figure 6). Taking the NBO analysis into account, the localised structures **B** and **C** have important contributions to the overall bonding picture. While representation of the type **A** is common for certain MICs, it does not reflect well the electron distribution for **2**. However, as the NBO analysis suggests a strongly delocalised structure, representation **D** depicts this feature in the best manner. In line with the MIC‐like character, formal charges are required for the representation of all limit structures.

In the case of the neutral metal complexes **7** and **8**, the HOMO and the LUMO are very similar to the ones of the free carbene **2**, while the HOMO−1 is a Rh‐based MO with a predominant d‐character. Interestingly, the energy of the HOMO (ligand and Fe based) is significantly influenced by the nature of the ligand on the rhodium atom, that is, the HOMO is stabilised by 0.26 eV in the case of **8** (−5.12 eV), which contains the strong π‐accepting CO ligands, compared to **7** (−4.86 eV). As expected, this stabilisation is even more pronounced for the rhodium‐based HOMO−1 (by 0.70 eV). An NBO analysis reveals a bonding situation very similar to the one described for **2**. Additionally, the carbene lone pair is involved in a 3c/4e interaction with the *trans* Rh−CO bond, whereas the *cis* Rh−CO bond is involved in a 3c/4e interaction with the chloride ligand.^41^, ^42^ In the case of the formal methanol addition product **11**, NBO analysis reveals that the N^2^‐C^19^‐C^18^ (numbering found in Figure 4) form a 3c/4e electron interaction, formally in a strong donor‐acceptor interaction between a partially filled p orbital at C^18^ and the π* MO of the N^2^‐C^19^ imine. This feature leads to partial loss of double bond character in of the N^2^‐C^19^ imine and a partial gain in double bond character for C^19^‐C^18^, a feature also verified through NMR spectroscopy and X‐ray crystallography (vide supra).^37^

**Figure 7:**
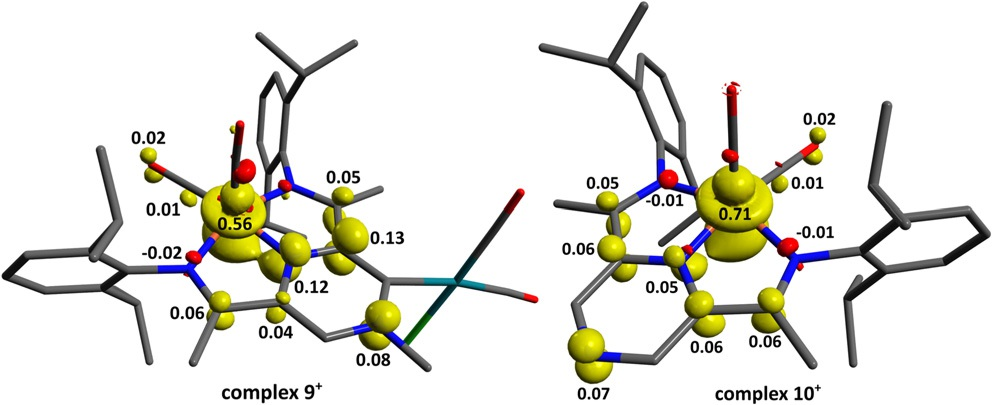
Spin density distribution (isovalue 0.005) of **9^+^** (left) and **10^+^** (right) (B3LYP, def2‐TZVP(‐f)) based on a Mulliken population analysis.

Lastly, we wanted to address the discrepancy between distribution of the unpaired electron in **9** (ligand‐based) and **10** (Fe‐based) observed by EPR.^43^ In the case of **10**, the SOMO was found to be a Fe‐based MO with a predominant d‐character, while the spin‐density plot obtained from a Mulliken‐population analysis reveals 0.71 spin density on the iron centre (Figure 6, right). This observation is in line with the rhombic signal obtained by EPR spectroscopy. On the other hand, for **9**, the qualitative MO diagram and Mulliken population analysis reveals that *(i)* the spin density is more delocalised over the entire ligand framework, with 0.56 spin density on the iron centre (Figure 7, left) and *(ii)* the SOMO (−8.70 eV) is stabilised by 0.21 eV compared to the rhodium‐based HOMO (−8.49 eV). The extent of spin delocalisation on the ligand explains the low g anisotropy observed by EPR spectroscopy.

## Conclusion

We have shown that P^Pz^DI‐type systems are able to support a bimetallic architecture, where the heterocyclic core is able to ligate both a NNN pincer‐type chelating system, while simultaneously acting as an N‐heterocyclic carbene, which can coordinate to a second metal centre. For the heterobimetallic Fe/Rh system studied herein, the NHC exhibits strong σ‐donating/π‐accepting properties. The carbene exhibits a special bonding situation, possessing both the characteristics specific to mesoionic carbenes, explaining the strong σ‐donating properties. Furthermore, the system possesses low lying π* molecular orbitals of the appropriate symmetry for backbonding, due to extensive delocalization of the π electrons over the P^Pz^DI system. These properties can be modulated through reversible chemical oxidation. As both oxidised and reduced forms are isolable, we could directly compare the effect of oxidation on the carbene properties by spectroscopic and computational methods. These studies reveal that, in contrast to standard iron PDI chemistry, where oxidation takes place at the metal, in the case of the heterobimetallic complexes, the unpaired electron is evenly distributed on the ligand core, therefore impacting the electronic properties of both metals involved. The reversible modulation of electronic properties was then applied in the catalytic hydrosilylation reaction of 4,4′‐difluorobenzophenone, where the oxidised species shows a ten‐fold increase in the reaction rate. We have shown that the switch between a slow‐rate regime and a fast‐rate regime could be achieved in situ through the addition of the appropriate external stimulus (oxidising or reducing agent) We have also demonstrated the ability of the NHC‐type Fe‐P^Pz^DI fragment to reversibly activate various alcohols and amines, likely through successively reversible formal oxidative addition/reductive elimination steps at the Cα. The formal oxidative addition of alcohols and amines is accompanied by the dearomatisation of the pyrazinium ring. This reactivity pattern is likely due to the ambiphilicity of the systems as a result of combining a π‐acidic pyrazine system with a Fe(CO)_2_ fragment which prevents the second nitrogen atom from engaging in the stabilisation of the carbene atom. It remains to be established if the redox‐state dependent change in reaction rate or chemoselectivity of the bimetallic species is general, and can be fostered for the design of redox‐switchable bimetallic catalysts. It is also to be expected that increasing the steric bulk of the substituents at the periphery of the carbene centre would allow the isolation of the free NHC. These directions are currently pursued in our laboratory.

## Conflict of interest

The authors declare no conflict of interest.

## Supporting information

As a service to our authors and readers, this journal provides supporting information supplied by the authors. Such materials are peer reviewed and may be re‐organized for online delivery, but are not copy‐edited or typeset. Technical support issues arising from supporting information (other than missing files) should be addressed to the authors.

Supplementary
