# Supplementary material for: A Redox‐Active Heterobimetallic N‐Heterocyclic Carbene Based on a Bis(imino)pyrazine Ligand Scaffold
Source: Angew Chem Int Ed Engl. 2020 Aug 28;59(43):19320–8. doi: 10.1002/anie.202005865 (PMC7590088; doi:10.1002/anie.202005865)
Supplement: Supplementary file 1 — Supplementary [file ANIE-59-19320-s001.pdf]

Angewandte Chemie
Eine Zeitschrift der Gesellschaft Deutscher Chemiker

Supporting Information

A Redox-Active Heterobimetallic N-Heterocyclic Carbene Based on a Bis(imino)pyrazine Ligand Scaffold

*Nicolas I. Regenauer[+], Sven Jänner[+], Hubert Wadepohl, and Dragoş-Adrian Roşca**

anie_202005865_sm_miscellaneous_information.pdf

Table of Contents

General S2

Synthetic Procedures S3

Stoichiometric alcohol dehydrogenation mechanism S12

Electronic Properties comparison with standard NHCs S13

Supporting Crystallographic Information S14

NMR Spectra and overview of the ^{15}N chemical shifts S21

IR Spectra and overview of the CO stretching frequencies S39

Cyclic Voltammetry S45

EPR Spectroscopy S46

Kinetic data S48

Computational Details S50

Cartesian Coordinates for Optimised Structures S61

General. Unless otherwise stated, all manipulations were performed using standard Schlenk techniques under dry argon in flame-dried glassware or in an argon filled MBraun glovebox. Anhydrous solvents were freshly distilled from appropriate drying agents (CH_2Cl_2 and CH_3CN over CaH_2, THF over Na/benzophenone, EtOH over Na) or dried over activated alumina columns (M. Braun SPS 800 - Et_2O, hexane, toluene, methanol) and were transferred under argon.

Flash chromatography: Merck silica gel 60 (40-63 µm).

MS EI: Finnigan MAT 8200 (70 eV), ESI-MS: ESQ 3000 (Bruker). Accurate mass determinations: JEOL JMS-700 magnetic sector at the mass spectrometry facility of the Institute for Organic Chemistry at the University of Heidelberg.

NMR spectroscopy ^{1}H, $^{13}C\{^{1}H\}$, $^{19}F\{^{1}H\}$ (referenced externally to CCl_3F), $^{31}P\{^{1}H\}$ (referenced externally to H_3PO_4). ^{15}N NMR data were obtained from ^{15}N-^{1}H HMBC measurements. NMR spectra were recorded using a Bruker Avance VIII-400 or Bruker Avance III HD 600 MHz spectrometer. Deuterated solvents were distilled from the appropriate drying agents, degassed by three freeze-pump-thaw cycles and stored over 4 Å molecular sieves prior to use. ^{1}H NMR spectra (400.1 MHz or 600.1 MHz) were referenced to the residual protons of the deuterated solvent used. $^{13}C\{^{1}H\}$ NMR spectra were referenced internally to the D-coupled ^{13}C resonances of the NMR solvent. Where appropriate, resonances were assigned using 2D NMR homo- and heterocorrelation (COSY, HMBC, HSQC) techniques. Chemical shifts (δ) are given in ppm, relative to TMS, coupling constants (*J*) in Hz.

Cyclic Voltammetry data were collected in a THF solution (1 mM) with $[nBu_4N][B(C_6F_5)_4]$[1] (0.1 M) electrolyte, using a 3 mm glassy carbon working electrode, platinum wire as the counterelectrode, and silver wire as the reference electrode in a flame-dried and degassed electrochemical cell, under an argon atmosphere. The glassy carbon electron was freshly polished and sonicated before each measurement. THF was freshly dried over Na/benzophenone and distilled prior to use. CVs were recorded using a EG & G Princeton electrochemical workstation. Unless otherwise stated, all CVs were run at a scan rate of 100 mV/s at 295 K. Potentials are reported versus the ferrocene/ferrocenium couple.[2]

---

[1] E. J. Lawrence, V. S. Oganesyan, G. G. Wildgoose and A. E. Ashley *Dalton Trans.* **2013**, *42*, 782.
[2] E. W. Geigner, *Organometallics* **2007**, *26*, 5738.

Continuous-wave X-band EPR spectra (ca. 9 GHz) were measured on a Bruker Biospin Elexsys E500 EPR spectrometer fitted with super high Q cavity. The magnetic field and the microwave frequency were calibrated with a Bruker ER 041XK Teslameter and a Bruker microwave frequency counter. The temperature of the sample was adjusted using a flow-through cryostat in conjuction with a Eurotherm (B-VT-2000) variable temperature controller. EPR spectra simulations were carried out using the EasySpin module run through Matlab R2019b.

IR: ATR (solid state) measurements were performed using a Bruker Alpha Spectrometer. Solution measurements were performed on an Agilent 3100 FT-IR Spectrometer (Excalibur Series), using a liquid cell.

Elemental Analyses were carried out on a Elementar vario MICRO cube in the Microanalysis Laboratory of the Heidelberg Chemistry Department.

Literature known compounds are referenced in the synthetic procedures.

Preparation of precursors

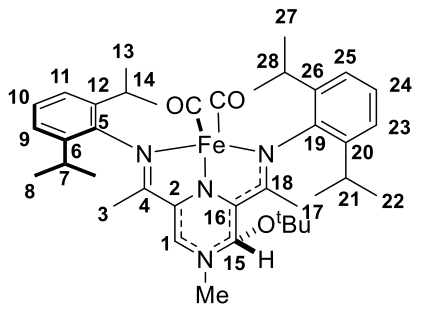

[(Me)(P^{pz}DI)(^{t}BuOH)Fe(CO)$_2$] (3) A suspension of **[(Me)(P^{pz}DI)Fe(CO)$_2$]I (1 · [I])**[3] (30 mg, 0.047 mmol) in C_6D_6 (1 mL) was treated with solid KO^{t}Bu (4.56 mg, 0.047 mmol) and the resulting purple mixture was stirred for 20 min. The solution was then passed through a syringe filter and transferred in an NMR tube. NMR data shows that conversion to the title compound was quantitative.

^{1}H NMR (400 MHz, C_6D_6) δ 7.18 – 7.02 (m, 6H, C_9H - C_{11}H + C_{23}H- C_{25}H), 6.37 (s, 1H, C_1H), 6.08 (s, 1H, C_{15}H), 3.37 – 3.14 (apparent m, CH ^{i}Pr, 4H), 2.45 (s, 3H, NCH_3), 1.84 (s, 3H, C_{17}H), 1.62 (s, 3H, C_3H), 1.58 (d, J = 6.7 Hz, 3H, CH_3 ^{i}Pr),1.49 (d, J = 6.7 Hz, 3H, CH_3 ^{i}Pr), 1.47 (d, J = 6.7 Hz, 3H, CH_3 ^{i}Pr), 1.41 (d, J = 6.7 Hz, 3H, CH_3 ^{i}Pr), 1.22 (d, J = 6.9 Hz, 3H), 1.15 (d, J = 6.9 Hz, 3H), 1.07 (s, 9H, O^{t}Bu), 1.07 – 0.97 (d +d overlapping , J = 6.9 Hz, 6H). **^{13}C{^{1}H} NMR** (101 MHz, C_6D_6) δ 219.7 (CO), 219.3 (CO), 163.7 (C4/C2), 151.4 (C_q Ar), 147.3 (C_{18}), 146.5 (C_q Ar), 143.1 (C_q Ar), 141.3 (C_q Ar), 140.7 (C_q Ar), 140.2 (C_q Ar), 129.8 (C_1), 129.5 (C_4/C_2), 126.5 (CH Ar), 125.8 (CH Ar), 125.1 (C_{16}), 124.3 (CH Ar), 123.8 (CH Ar), 123.3 (CH Ar), 81.9 (C_{15}), 73.4 (O^{t}Bu), 40.6 (NCH_3), 31.5 (O^{t}Bu), 31.3 (CH ^{i}Pr), 29.6 (CH ^{i}Pr), 29.5 (CH ^{i}Pr), 28.3 (CH ^{i}Pr), 27.9 (CH_3 ^{i}Pr), 27.7 (CH_3 ^{i}Pr), 27.2 (CH_3 ^{i}Pr), 26.1 (CH_3 ^{i}Pr), 25.2 (CH_3 ^{i}Pr), 25.0 (CH_3 ^{i}Pr), 24.3 (CH_3 ^{i}Pr), 15.4 (C_3), 14.9 (C_{17}).

[3] N. I. Regenauer, S. Settele, E. Bill, H. Wadepohl, D.-A. Roșca *Inorg. Chem.* **2020**, *59*, 2604-2612

Placing a sample of **3** under vacuum for a prolonged amount of time, followed by dissolution in C_6D_6 indicated an indecipherable mixture of products by ^{1}H NMR spectroscopy. The compound is thermally unstable in solution, and signs of decomposition are observed by ^{1}H NMR spectroscopy at room temperature over 72h.

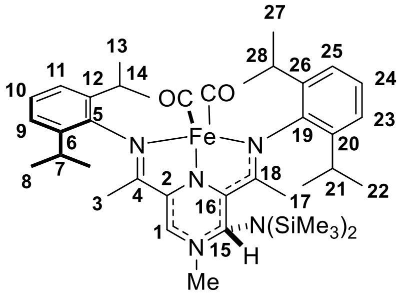

[(Me)(P^{pz}DI)(HMDS)Fe(CO)$_2$] (4) A suspension of **[(Me)(P^{pz}DI)Fe(CO)$_2$]I (1 · [I])** (30 mg, 0.0468 mmol) in C_6D_6 (1 mL) was treated with $KN(SiMe_3)_2$ (8.1 mg, 0.0468 mmol) and the resulting purple mixture was stirred for 20 min. The solution was then passed through a syringe filter and transferred in an NMR tube. NMR data shows that conversion to the title compound was 50-75%.

^{1}H NMR (600 MHz, C_6D_6) δ 7.26 – 6.98 (m, 6H, C_9H - C_{11}H + C_{23}H- C_{25}H), 6.29 (s, 1H, C_1H), 5.93 (s, 1H, C_{15}H), 3.48 (hept, *J* = 6.6 Hz, 1H, CH ^{i}Pr), 3.37 – 3.25 (p + p overlapping, *J* = 6.6 Hz, 2H, CH ^{i}Pr), 3.23 (p, *J* = 6.8 Hz, 1H ,CH ^{i}Pr), 2.23 (s, NCH_3, 1H), 1.67 (s, 3H, C_{17}H), 1.62 (s, 3H, C_3H), 1.57 (d, *J* = 6.6, 3H, CH_3 ^{i}Pr), 1.49 (d + d, overlapping, *J* = 6.8, 6H, CH_3 ^{i}Pr), 1.45 (d, *J* = 6.8 Hz, 3H, CH_3 ^{i}Pr), 1.22 (d + d, overlapping, *J* = 6.9, 6H, CH_3 ^{i}Pr), 1.12 (d + d, overlapping, *J* = 6.8 Hz, 6H, CH_3 ^{i}Pr), 0.15 (s, 9H, $SiMe_3$), 0.12 (s, 9H, $SiMe_3$). **^{13}C{^{1}H} NMR** (151 MHz, C_6D_6) δ 217.9 (CO), 217.7 (CO), 160.6 (C4/C2), 149.2 (C_q Ar), 144.9 (C_{18}), 143.2 (C_q Ar), 140.6 (C_q Ar), 139.4 (C_q Ar), 138.4 (C_q Ar), 138.3 (C_q Ar), 128.9 (C_1), 124.7 (C_{16}), 124.0 (CH Ar), 123.2(CH Ar), 121.9 (CH Ar), 121.5 (CH Ar), 121.4 (CH Ar), 120.9 (CH Ar), 68.9 (C_{15}), 37.9 (NMe), 25.8 (CH ^{i}Pr), 25.4 (CH ^{i}Pr), 25.3 (CH ^{i}Pr), 24.9 (CH ^{i}Pr), 23.5 (CH_3 ^{i}Pr), 23.2 (CH_3 ^{i}Pr), 22.7 (CH_3 ^{i}Pr), 22.5 (CH_3 ^{i}Pr), 22.4 (CH_3 ^{i}Pr), 22.3 (CH_3 ^{i}Pr), 22.2 (CH_3 ^{i}Pr), 14.0 (C_3), 12.4 (C_{17}), 1.3 ($SiMe_3$), 0.1 ($SiMe_3$).**^{29}Si – ^{1}H HMBC** (119 MHz, C_6D_6) δ 3.08 (cross-peak to δ_H 5.93 and 0.15), -22.3 (cross-peak to δ_H 0.12) **^{15}N – ^{1}H HMBC** (61 MHz, C_6D_6) 233.3 (N-Fe, cross peak to δ_H 6.29 and 5.93), 224.6 (C_4=N, cross peak to δ_H 1.62), 188.3 (C_{18}=N, cross peak to δ_H 1.67), 103.3 (NMe, cross peak to δ_H 6.29 and 2.23), δ 58.1 ($N(SiMe_3)_2$ cross peak to δ_H 5.93, 0.15 and 0.12). The sample contains residual $HN(SiMe_3)_2$.

Placing a sample of **4** under vacuum for a prolonged amount of time, followed by dissolution in C_6D_6 indicated an indecipherable mixture of products by ^{1}H NMR spectroscopy.

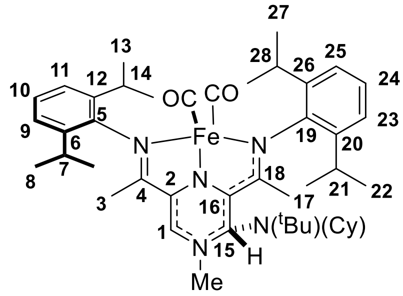

[(Me)(P^{pz}DI)(HN^{t}BuCy)Fe(CO)$_2$] (5) A suspension of **[(Me)(P^{pz}DI)Fe(CO)$_2$]I (1 · [I])** (30 mg, 0.047 mmol) in THF-d_8 (0.6 mL) was treated with $Li(OEt)_2N^tBuCy$ (8.1 mg, 0.047 mmol)[4] and the resulting purple mixture was stirred for 20 min. The solution was then passed through a syringe filter and transferred in an NMR tube. NMR data shows that conversion to the title compound was 40-50%. NMR data for **5**: ^{1}H NMR (600 MHz, THF-d_8) δ 7.40 (s, 1H, C_1H), 6.32 (s, 1H, C_{15}H), 3.28 (s, 3H, NMe), 3.06 (q, 1H, CH ^{i}Pr), 2.99 - 2.94 (m, 2H, CH ^{i}Pr), 2.88 (q, 1H, CH

[4] H. Fujieda, M. Kanai, T. Kambara, A. Iida, K. Tomioka *J. Am. Chem. Soc.* **1997***, 119*, 2060-2061

^{i}Pr), 2.46 – 2.37 (m, Cy), 1.87 (s, 3H, C3H), 1.70 – 1.67 (m, 8H), 1.34 (s, 3H, $C^{17}H$), 1.27 – 1.15 (m, 24H, CH_3 ^{i}Pr), 0.56 (s, 9H). – Spectrum contains $HN^{t}BuCy$ and unidentified impurities.

Trapping experiment

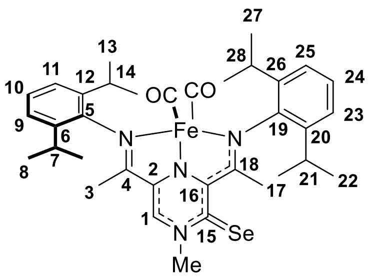

[(Me)(P^{pz}DI)(Se)Fe(CO)$_2$]. (6) A scintillation vial was charged with **1 · [I]** (30 mg, 0.047 mmol) and KHMDS (8.1 mg, 0.047 mmol), to which THF-d_8 (0.6 mL) was added. The reaction mixture was then stirred for 15 minutes, during which a colour change from brown to purple was noted. The resulting suspension was filtered to remove the formed KI, was subsequently treated with elemental Se (4 mg, 0.05 mmol) and transferred to an NMR tube. After 1h, full conversion to **6** was noted. The solvent was removed *in vacuo* and the resulting powder was washed with pentane (1 mL). ^{1}**H NMR** (400 MHz, THF-d_8) δ 8.71 (s, 1H, C_1H), 7.24 – 7.03 (m, 6H, C_9H-$C_{11}H$ + $C_{23}H$-$C_{25}H$), 4.11 (s, 3H, NMe), 2.74 (s, 3H, $C_3H/C_{17}H$), 2.63 (p, J = 7 Hz, 2H, CH^{i}Pr), 2.54 (p, J = 7 Hz, 2H, CH ^{i}Pr), 2.34 (s, 3H, $C_3H/C_{17}H$), 1.21 (dd + dd overlapping, J = 7 Hz, 12H, CH_3 ^{i}Pr), 1.03 (dd + dd, J = 7 Hz, 12H, CH_3 ^{i}Pr). ^{13}**C NMR** (101 MHz, THF-d_8) δ 214.9 (CO), 170.0 (C_{16}), 165.0 (C_4/C_{18}), 164.4 (C_4/C_{18}), 150.1 (C_q Ar), 147.0 (C_q Ar), 144.0 (C_q Ar), 140.2 (C_q Ar), 139.7, 137.1, 129.0 (C_1H), 126.9 (CH Ar), 126.1 (CH Ar), 123.7 (CH Ar), 123.3 (CH Ar), 47.7 (NMe), 27.6 (CH iPr), 27.4 (CH3 iPr), 25.4 (CH3 ^{i}Pr), 22.2 (CH_3 iPr), 15.5 (CH_3 ^{i}Pr), 14.7 (C_3/C_{17}), 13.4 (C_3/C_{17}). Missing CH_3 ^{i}Pr resonances are overlapping with the THF-d_8 residual signal. ^{15}**N –** ^{1}**H HMBC** (61 MHz, THF-d_8) 250.6 (cross peak to δ_H 2.32), 248.6 (N-Fe, cross peak to δ_H 8.71 and 2.77), 170.6 (NMe, cross peak to δ_H 4.11). ^{77}**Se NMR** (114 MHz, THF-d_8) 557.0 (C=Se) ^{77}**Se NMR** (114 MHz, $(CD_3)_2CO$ 536 (C=Se) **IR** (ATR, ν, cm^{-1}) 2961, 2867, 1992 (CO), 1938 (CO), 1617, 1462, 1396, 1364, 1274, 1196, 1102. Anal. Calcd. For $C_{40}H_{56}FeN_4O_2Se$ [M · pentane]: C, 63.24; H, 7.53; N, 7.37. Found: C, 63.80; H, 7.12; N, 7.57

Attempted reaction between [(Me)(P^{pz}DI)Fe(CO)$_2$]I and (PPh)$_5$. A scintillation vial was charged with **[(Me)(P^{pz}DI)Fe(CO)$_2$]I (1 · [I])** (30 mg, 0.047 mmol) and KHMDS (8.1 mg, 0.047 mmol), to which THF-d_8 (0.6 mL) was added. The reaction mixture was then stirred for 15 minutes, during which a colour change from brown to purple was noted. The resulting suspension was filtered to remove the formed KI, was subsequently treated with (PhP)$_5$ [5] (4.4 mg, 0.008 mmol) and transferred into an NMR tube. The reaction was monitored by NMR spectroscopy, showing gradual degradation of the in situ formed **4** (judged by ^{1}H NMR spectroscopy) and no consumption of (PhP)$_5$ (judged by ^{1}H and ^{31}P{^{1}H} NMR spectroscopy).

[5] L. Wu, S. S. Chitnis, H. Jiao, V. T. Annibale, I. Manners *J. Am. Chem. Soc.* **2017**, *139,* 16780-16790.

Preparation of bimetallic complexes

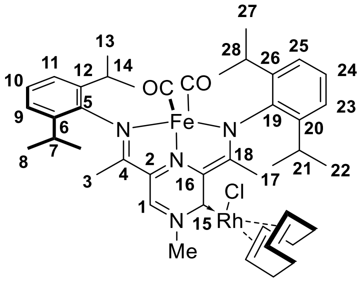

[(Me)(P^{pz}DI)Fe(CO)$_2$(Rh(COD)Cl)] (7). NMR experiment: A scintillation vial was charged with **(1 · [I]** (30 mg, 0.047 mmol) and KHMDS (8.1 mg, 0.041 mmol), to which THF-*d$_8$* (0.6 mL) was added. The reaction mixture was then stirred for 15 minutes, during which a colour change from brown to purple was noted. The resulting suspension was filtered to remove the formed KI, was subsequently treated with [Rh(COD)Cl]$_2$ (8.2 mg, 0.017 mmol) and transferred into an NMR tube. After 1 h, full conversion to **7** was noted.

Larger scale preparation. A Schlenk-flask was charged with **(1 · [I]** (200 mg, 0.271 mmol) and KHMDS (54 mg, 0.271 mmol). THF (5 mL) was subsequently added and the reaction mixture was stirred for 15 minutes, during which a colour change to purple was noted. The suspension was then filtered and [Rh(COD)Cl]$_2$ (55 mg, 0.112 mmol) was added. Stirring was continued for 16 hours. Subsequently, the volatile components were removed *in vacuo* and the resulting dark residue was washed with cold pentane (1 mL), to give the title compound as a dark brown solid. Yield 167 mg (88 %).[6]

^{1}H NMR (400 MHz, THF-*d$_8$*) δ 8.70 (s, 1H, C$_1$H), 7.17 – 6.84 (m, C$_9$H-C$_{11}$H + C$_{23}$H-C$_{25}$H), 4.99 (s, 2H, COD), 4.95 (s, 3H, NMe), 3.66* (s, C$_{17}$H/C$_3$H) 2.96 (br t, 1H, *J* = 14 Hz, COD), 2.84 (br t, 1H, *J* = 14 Hz, CH COD) 2.74 (s, 1H), 2.61 – 2.50 (m, 3H, CH$_2$ – COD + CH ^{i}Pr, overlapping), 2.43 – 2.33 (m, 5H, CH$_2$ – COD + CH ^{i}Pr, overlapping), 2.30 (s, 3H, C$_3$H/C$_{17}$H), 2.24 – 2.17 (m, 2H, CH ^{i}Pr), 2.02 – 1.91 (CH$_2$-COD) 1.20 (d, 6H, *J* = 8 Hz, CH$_3$ ^{i}Pr), 1.04 (d, 6H, *J* = 8 Hz, CH$_3$ ^{i}Pr), 0.89 (d, 6H, *J* = 8 Hz, CH$_3$ ^{i}Pr), 0.86 (d, 6H, *J* = 8 Hz, CH$_3$ ^{i}Pr) **^{13}C{^{1}H} NMR** (151 MHz, THF-*d$_8$*) δ 214.9 (CO), 213.6 (CO), 205.6 (d, $^1J_{RhC}$ = 43.6 Hz, C$_{15}$), 168.0 (C$_4$/C$_{18}$), 163.8 (C$_4$/C$_{18}$), 149.4, 146.4 (C$_2$/C$_{16}$), 140.7 (C$_q$ Ar), 140.4 (C$_q$ Ar), 139.8 (C$_2$/C$_{16}$), 139.4 (C$_q$ Ar), 139.1 (C$_q$ Ar), 130.6 (C$_1$), 126.9 (CH Ar), 126.4 (CH Ar), 123.8 (CH Ar), 123.7 (CH Ar), 123.6 (CH Ar), 123.4 (CH Ar), 97.4 (d, J_{RhC} = 7 Hz, CH COD), 96.5 (d, J_{RhC} = 7 Hz, CH COD), 68.3 (d, J_{RhC} = 14.5 Hz, CH COD), 67.7 (d, J_{RhC} = 14.5 Hz, CH COD) 52.2 (NMe), 33.6 (CH$_2$, COD), 31.6 (CH$_2$, COD), 30.7 (CH$_2$-COD), 29.5 (CH$_2$-COD), 27.9 (CH iPr), 27.7 (CH ^{i}Pr), 27.5 (CH iPr), 27.3 (CH ^{i}Pr), 24.4* (CH$_3$ ^{i}Pr), 24.2* (CH$_3$ ^{i}Pr) 24.0* (CH$_3$ ^{i}Pr), 23.5 (CH$_3$ ^{i}Pr), 22.4 (C$_3$/C$_{17}$), 15.4 (C$_3$/C$_{17}$). **^{15}N – ^{1}H HMBC** (61 MHz, THF-*d$_8$*) 256.0 (C=N, cross peak to δ$_H$ 2.30), 235.1 (cross peak to δ$_H$ 3.66), 233.0 (N-Fe, cross peak to δ$_H$ 8.70), 176.1 (NMe, cross peak to δ$_H$ 4.95 and 8.73). (* denotes signals overlapping with THF-*d$_8$* or residual THF in THF-*d$_8$*, which have been assigned through 2D-NMR spectroscopy) **IR** (ATR, ν, cm^{-1}) 2961, 2867, 1980 (CO), 1919 (CO), 1611, 1449, 1403, 1383, 1256, 1198, 1100. Anal. Calcd. for $C_{37}H_{44}ClFeN_4O_4Rh$: C, 60.40 H, 6.60; N, 6.55. Found: C, 59.88; H, 6.98; N, 6.91.

---

[6] The yield is reported with respect to the limiting reagent ([Rh(COD)Cl]$_2$)

[(Me)(P^pz DI)Fe(CO)$_2$(Rh(CO)$_2$Cl)] (8). NMR experiment. A scintillation vial was charged with **1 · [I]** (30 mg, 0.047 mmol) and KHMDS (8.1 mg, 0.041 mmol), to which THF-*d*$_8$ (0.6 mL) was added. The reaction mixture was then stirred for 15 minutes, during which a colour change from brown to purple was noted. The resulting suspension was filtered to remove the formed KI, was subsequently treated with $[Rh(CO)_2Cl]_2$ (6.7 mg, 0.017 mmol) and transferred into an NMR tube. After 1 h, full conversion to **8** was noted.

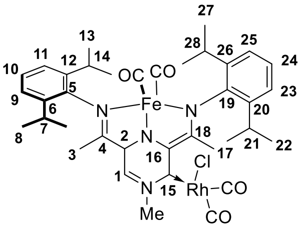

Larger scale preparation. A Schlenk flask was charged with **1 · [I]** (0.250 g, 0.339 mmol) and KHMDS (0.068 g, 0.339 mmol). Subsequently, THF (6.0 mL) was added, and the reaction mixture was stirred for 15 minutes. A colour change from black to purple was noted. The mixture was then filtered and subsequently treated with $[Rh(CO)_2Cl]_2$ (0.046 g, 0.119 mmol). Stirring was continued for 1 hour. The solvent was then removed *in vacuo* and the resulting brown residue was washed with cold pentane (3 × 1 mL), to give the title complex as a brown powder. Yield: 0.183 g (96%).[7]

^{1}H NMR (400 MHz, THF-*d*$_8$) δ 8.87 (s, 1H, C_1H), 7.25 – 7.11 (m, 6H, C_9H-$C_{11}H$ + $C_{23}H$-$C_{25}H$), 4.64 (s, 3H, NMe), 3.09 (s, 3H, $C_{17}H/C_3H$), 2.50 – 2.41 (p + p overlapping *J* = 7.6 Hz, 2H, CH ^{i}Pr), 2.37 – 2.28 (p + p overlapping *J* = 7.6 Hz, 2H, CH ^{i}Pr) 2.36 (s, 3H, $C_{17}H/C_3H$), 1.19 – 0.91 (m, 24H, overlapping non-equivalent CH_3 ^{i}Pr) **^{13}C{^{1}H} NMR** (101 MHz, THF-*d*$_8$) δ 212.8 (Fe-CO), 212.7 (Fe-CO), 186.5 (d, *J* = 53.4 Hz, Rh-CO *cis*), 184.9 (d, *J* = 37.0 Hz, C_{15}), 183.0 (d, *J* = 76.0 Hz, Rh-CO *trans*), 167.7 (C_4/C_{18}), 163.4 (C_4/C_{18}), 148.1 (C_q Ar), 146.3 (C_q Ar), 146.1 (C_2/C_{16}), 139.6 (C_q Ar), 139.4 (C_q Ar), 139.2 (C_2/C_{16}), 138.8 (C_q Ar), 138.7 (C_q Ar), 133.7 (C_1H), 126.4 (CH Ar), 126.0 (CH Ar), 123.4 (CH Ar), 123.1 (CH Ar), 123.0 (CH Ar), 122.9 (CH Ar), 122.7, 53.3 (NMe), 27.8 (CH ^{i}Pr), 26.8 (CH ^{i}Pr, 2 resonances overlapping), 26.7 (CH ^{i}Pr), 24.7 (CH_3 ^{i}Pr), 23.2* (CH_3 ^{i}Pr), 23.0 (CH_3 ^{i}Pr), 22.8 (CH_3 ^{i}Pr), 21.9 (C_3/C_{17}), 15.8 (C_3/C_{17}). **^{15}N – ^{1}H HMBC** (61 MHz, THF-*d*$_8$) 256.4 (C=N, cross peak to δ_H 2.36), 243.5 (C=N, cross peak to δ_H 3.09), 171.4 (NMe, cross peak to δ_H 4.64). (* denotes signals overlapping with THF-*d*$_8$ or residual THF in THF-*d*$_8$, which have been assigned through 2D-NMR spectroscopy). **IR** (ATR, ν, cm^{-1}) 2963, 2870, 2066 (Rh-CO), 1988 (Rh-CO + Fe-CO overlapping), 1926 (Fe-CO), 1612, 1437, 1384, 1363, 1254, 1201, 1102. **IR** (KBr, CH_2Cl_2, ν, cm^{-1}) 2074 (Rh-CO), 1995 (Fe-CO + Rh-CO), 1931 (Fe-CO). Anal. Calcd. for $C_{37}H_{44}ClFeN_4O_2Rh$ [M]: C, 55.34; H, 5.52; N, 6.98. Found: C, 55.58; H, 5.77; N, 7.00.

[7] The yield is reported with respect to the limiting reagent ($[Rh(CO)_2Cl]_2$)

Redox chemistry

Oxidation

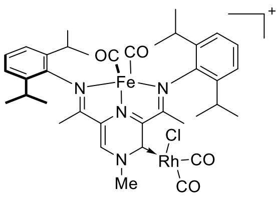

[(Me)(P^{pz}DI)Fe(CO)$_2$(Rh(CO)$_2$Cl)][BArF$_{24}$] (9). A scintillation vial was charged with [(Me)(P^{pz}DI)Fe^0(CO)$_2$Rh^I(CO)$_2$Cl] (0.183 g, 0.228 mmol) and dissolved in CH_2Cl_2 (6 mL). The resulting solution was treated with [Fc][BArF$_{24}$][8] (0.239 g, 0.228 mmol), resulting in a colour change from dark brown to dark green. Stirring was continued for 1 hour. Removal of the volatile components *in vacuo* resulted in a black paste, which was washed with toluene/pentane (1/1, 3 × 2 mL). The resulting solid was then dried under reduced pressure to give **9** as a black powder. Yield 0.280 g (74%).

^{1}H NMR (600 MHz, CD_2Cl_2) δ 8.34 (134 Hz), 7.70 (140 Hz), 7.51 (105 Hz), 7.24 (214 Hz), 4.44 (267 Hz), 3.02 (300 Hz), 2.33 (388 Hz), 1.10 (356 Hz). **Magnetic susceptibility (Evans):** μ_{eff} = 1.91 μ_B ($CHDCl_2$ in CD_2Cl_2, 298 K). **IR** (ATR, ν, cm^{-1}) 2969, 2932, 2874, 2084 (Rh-CO), 2004 (Rh-CO + Fe-CO), 1944 (Fe-CO), 1611, 1464, 1353, 1273, 1116. **IR** (KBr, CH_2Cl_2, ν, cm^{-1}) 2081 (Rh-CO), 2007 (Fe-CO + Rh-CO), 1948 (Fe-CO). Anal. Calcd. for $C_{69}H_{56}BClF_{24}FeN_4O_4Rh$ [M]: C, 49.74; H, 3.39; N, 3.36. Found: C, 49.42; H, 3.46; N, 3.19.

Reduction

A scintillation vial was charged with **9** (0.050 g, 0.030 mmol) and dissolved in CH_2Cl_2 (2.0 mL). The resulting solution was treated with $CoCp_2$ (5.7 mg, 0.030 mmol), resulting in a colour change from dark green to dark brown. Stirring was continued for 1 hour. The volatile components were removed *in vacuo* and the product was extracted with benzene. The benzene solution was taken to dryness and the resulting residue was dissolved in CD_2Cl_2 and analysed by ^{1}H NMR spectroscopy, which showed quantitative formation of **8**.

Oxidation of model

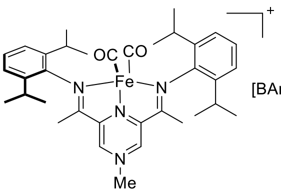

[(P^{pz}DI)Fe(CO)$_2$][BArF$_{24}$] (10). A scintillation vial was charged with [(P^{pz}DI)Fe(CO)$_2$[3] (0.150 mg, 0.252 mmol) and C_6H_6 (3 mL) was added. Subsequently, [Fc][BArF$_{24}$] (0.265 g, 0.252 mmol) was added and stirring was continued for 1h. Pentane (3 mL) was added, and the resulting suspension was filtered. The resulting precipitate was washed with pentane (3 x 1 mL) and was dried *in vacuo* to give the title compound as a dark green solid which was further recrystallized from layering a CH_2Cl_2 solution with pentane. The crystals were suitable for X-ray diffraction. Yield 0.328 g, 78 % **Magnetic susceptibility (Evans)** μ_{eff} = 1.94 μ_B ($CHDCl_2$ in

[8] I. Chavez, A. Alvarez-Carena, E. Molins, A. Roig, W. Maniukiewicz, A. Aranciba, V. Aranciba, H. Brand, J. M. Manriquez *J. Organomet. Chem.* **2000**, *601*, 126-132.

CD_2Cl_2, 298 K) **IR** (ATR, ν, cm^{-1}) 2971, 2968, 2024 (CO), 1986 (CO), 1602 (C=N), 1353, 1273, 1115. Anal. cald. for $C_{72}H_{67}BCl_2F_{24}FeN_4O_2$ (M · CH_2Cl_2 · pentane): C, 53.58; H, 4.18; N, 3.47. Found: C, 52.91; H, 4.71; N, 3.23

Alcohol and amine exchange

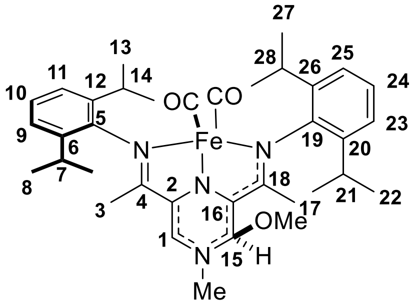

[(Me)(P^{pz}DI)(MeOH)Fe(CO)$_2$]. (11) To a suspension of **1 · [I]** (0.200 mg, 0.272 mmol) in benzene (10 mL), excess methanol (0.5 mL, 12.33 mmol) was added, followed by KO^{t}Bu (0.030 g, 0.272 mmol). An instant colour change from dark brown to purple was observed. The reaction mixture was stirred for 10 minutes, and the KI was filtered off. The supernatant was then concentrated *in vacuo* to give the title compound as a purple powder. Yield: 89%, 0.155 mg. Crystals suitable for single-crystal X-ray diffraction were grown from a concentrated pentane solution at -35°C.

^{1}H NMR (600 MHz, C_6D_6) δ 7.28 – 7.11 (m, 6H, C_9H - C_{11}H + C_{23}H + C_{25}H), 6.57 (s, 1H, C_1H), 5.94 (s, 1H, C_{15}H), 3.46 (p, *J* = 6.9 Hz, 1H, CH ^{i}Pr), 3.34 (q, *J* = 6.7 Hz, 1H, CH ^{i}Pr), 3.29 (q, *J* = 6.9 Hz, 1H, CH ^{i}Pr), 3.22 (q, *J* = 6.9 Hz, 1H, CH ^{i}Pr), 2.93 (s, 3H, OCH_3), 2.50 (s, 3H, NCH_3), 1.88 (s, 3H, C_{17}H), 1.69 (s, 3H, C_3H), 1.65 (d, *J* = 6.7 Hz, 3H, CH_3 ^{i}Pr), 1.59 (d, *J* = 6.8 Hz, 3H, CH_3 ^{i}Pr), 1.56 (d, *J* = 6.7 Hz, 3H, CH_3 ^{i}Pr), 1.53 (d, *J* = 6.8 Hz, 3H, CH_3 ^{i}Pr), 1.30 (d, *J* = 6.9 Hz, 3H, CH_3 ^{i}Pr), 1.26 (d, *J* = 6.9 Hz, 3H, CH_3 ^{i}Pr), 1.13 (dd, *J* = 6.9, 3.7 Hz, 6H, CH_3 ^{i}Pr). **^{13}C{^{1}H} NMR** (151 MHz, C_6D_6) δ 219.7 (CO), 219.2 (CO), 163.3 (C_4/C_2), 151.1 (C_q Ar), 147.0 (C_{18}), 146.9 (C_q Ar), 142.6 (C_q Ar), 141.6 (C_q Ar), 140.3 (C_q Ar), 139.9 (C_q Ar), 130.2 (C_1), 129.1 (C_4/C_2), 126.3 (*p*-Ph), 125.5 (*p*-Ar), 123.9 (CH Ar), 123.7 (CH Ar), 123.5 (C_{16}), 123.4 (CH Ar), 123.1 (CH Ar), 83.6 (C_{15}), 48.4 (OCH_3), 40.0 (NCH_3), 27.9 (CH ^{i}Pr), 27.7 (CH ^{i}Pr), 27.6 (CH ^{i}Pr), 27.4 (CH ^{i}Pr), 25.7 (CH_3 ^{i}Pr), 25.3 (CH_3 ^{i}Pr), 25.0(CH_3 ^{i}Pr), 24.7 (CH_3 ^{i}Pr), 24.6 (CH_3 ^{i}Pr), 24.5 (CH_3 ^{i}Pr), 24.1 (CH_3 ^{i}Pr), 24.0 (CH_3 ^{i}Pr), 14.6 (C_3), 14.5 (C_{17}). **^{15}N – ^{1}H HMBC** (61 MHz, C_6D_6) 231.4 (N-Fe, cross peak to δ_H 6.57 and 5.94 and C_4=N, cross peak to δ_H 1.69, overlapping), 188.2 (C_{18}=N, cross peak to δ_H 1.88), 94.8 (NMe, cross peak to δ_H 6.57 and 2.50). **Magnetic susceptibility (Evans)** μ_{eff} = 0 μ_B ($SiMe_4$ in CD_2Cl_2, 298 K) **IR** (ATR, ν, cm^{-1}) 2966, 2927, 2837, 1949 (CO), 1882 (CO), 1617, 1440, 1384, 1334, 1287, 1194, 1028. **IR** (pentane, ν, cm^{-1}) 1974 (CO), 1906 (CO). Anal. Calcd. for $C_{36}H_{48}FeN_4O_3$: C, 67.49; H, 7.55; N, 8.75. Found: C, 66.80; H, 7.81; N, 8.48.

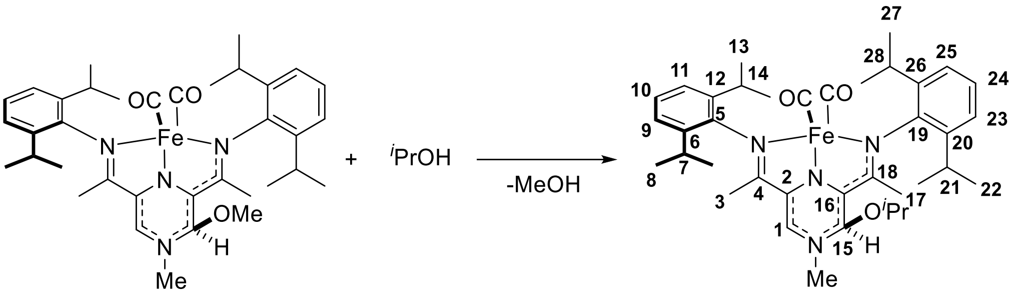

[(Me)(P^{pz}DI)(^{i}PrOH)Fe(CO)$_2$] (12) A solution of **11** (45 mg, 0.07 mmol) in C_6H_6 (3 mL) was treated with excess isopropanol (0.1 mL, 1.30 mmol) and the resulting mixture was stirred at room temperature for 5 min. The volatile components were removed *in vacuo* to give the title compound as a purple powder. (95%, 44 mg).

^{1}H NMR (600 MHz, C_6D_6) δ 7.29 – 7.12 (m, 6H, C_9H - C_{11}H + C_{23}H + C_{25}H), 6.60 (s, 1H, C_1H), 5.98 (s, 1H, C_{15}H), 3.62 (p, *J* = 6.1 Hz, 1H, CH O^{i}Pr), 3.45 (p, *J* = 6.8 Hz, 1H, CH ^{i}Pr), 3.36 (p, *J* = 6.8 Hz, 1H, CH ^{i}Pr), 3.28 (p, *J* = 6.6 Hz, 1H, CH ^{i}Pr), 3.24 (p, *J* = 6.6 Hz, 1H, CH ^{i}Pr), 2.56 (s, 3H, NCH_3), 1.91 (s, 3H, C_{17}H), 1.72 (s, 3H, C_3H), 1.66 (d, *J* = 6.6 Hz, 3H, CH_3 ^{i}Pr), 1.56 (d + d overlapping, *J* = 6.8, 6H, CH_3 ^{i}Pr), 1.51 (d, *J* = 6.8 Hz, 3H, CH_3 ^{i}Pr), 1.32 (d, *J* = 6.8 Hz, 3H, CH_3 ^{i}Pr), 1.24 (d, *J* = 6.9 Hz, 3H, CH_3 ^{i}Pr), 1.11 (d + d overlapping, *J* = 6.8 Hz, 6H, CH_3 ^{i}Pr), 1.06 (d, *J* = 6.1 Hz, 6H, CH_3 O^{i}Pr). **^{13}C{^{1}H} NMR** (151 MHz, C_6D_6) δ 219.4 (CO), 219.3 (CO), 163.3 (C_4), 151.1 (C_q Ar), 147.0 (C_{18}), 146.6 (C_q Ar), 142.9 (C_q Ar), 141.2 (C_q Ar), 140.4 (C_q Ar), 139.8 (C_q Ar), 129.9 (C_1), 126.3 (CH Ar), 125.6 (CH Ar), 124.3 (C_{18}), 124.1 (CH Ar), 123.6 (CH Ar), 123.5 (CH Ar), 123.1 (CH Ar), 82.9 (C_{15}), 65.6 (CH O^{i}Pr), 40.2 (NCH_3), 28.0 (CH ^{i}Pr), 27.6 (CH ^{i}Pr), 27.5 (CH ^{i}Pr), 27.1 (CH ^{i}Pr), 25.9 (CH_3 ^{i}Pr), 25.7 (CH_3 ^{i}Pr), 25.2 (CH_3 ^{i}Pr), 24.9 (CH_3 ^{i}Pr), 24.8 (CH_3 ^{i}Pr), 24.2 (CH_3 ^{i}Pr), 24.0 (CH_3 ^{i}Pr), 23.9 (CH_3 ^{i}Pr), 14.7 (C_3), 14.6 (C_{17}). Sample used for NMR measurements contains traces of isopropanol. **IR** (ATR, ν, cm^{-1}) 2962, 2925, 2867, 1947 (CO), 1880 (CO), 1617, 1521, 1437, 1381, 1362, 1320, 1242, 1194, 1176, 1090, 1055.

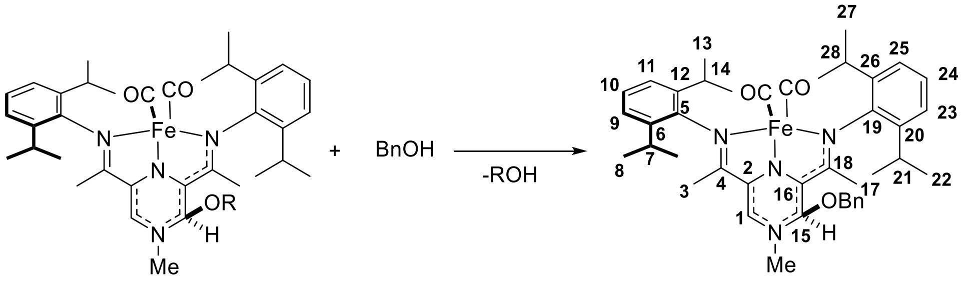

[(Me)(P^{pz}DI)(BnOH)Fe(CO)$_2$] (13) Procedure A. To a suspension of **1 · [I]** (0.200 g, 0.272 mmol) in benzene (10 mL), KO^{t}Bu (0.031 g, 0.272 mmol) was added. An instant colour change from dark brown to purple was observed. The mixture was stirred at room temperature for 20 minutes and the resulting precipitate (KI) was filtered. The supernatant was then treated with pentane (3 mL) and benzylic alcohol (85 µL, 0.813 mmol). Stirring was continued for an additional 30 minutes, during which, a precipitate was observed. The purple powder was collected, washed with 1 mL cold pentane and was briefly dried under vacuum for one minute. Yield: 40%, 0.07 g. **Procedure B.** In a J-Young NMR tube, a solution of **11** (15 mg, 0.023 mmol) in C_6D_6 (0.6 mL) was treated with BnOH (3 eq) (7.5 µL, 0.072 mmol). The NMR tube was briefly shaken and placed in an NMR spectrometer. A conversion of 75% to **13**, alongside unreacted starting material and methanol was observed. The conversion did not improve upon the subsequent addition of 2 eq BnOH.

^{1}H NMR (400 MHz, C_6D_6) δ 7.50 – 6.96 (m, Bn + C_9H - C_{11}H + C_{23}H + C_{25}H, 11H), 6.38 (s, C_1H, 1H), 5.98 (s, 1H, C_{15}H), AB spin system with A: 4.23 B: 4.06 (^{2}J = 11.5 Hz, 4H, CH_2 of Bn), 3.42 (br p, *J* = 6.6 Hz, 1H, CH ^{i}Pr), 3.28 – 3.01

(apparent m, 3H, CH ^{i}Pr), 2.44 (s, 3H, NMe), 1.82 (s, 3H, C_{17}H), 1.61 (s, 3H, C_3H), 1.55 – 1.38 (apparent m, 12H, CH_3 ^{i}Pr), 1.16 (dd, J = 11.0, 6.9 Hz, 5H), 1.06 (dd, J = 15.2, 6.8 Hz, 5H). **^{13}C{^{1}H} NMR** (101 MHz, C_6D_6) δ 219.9 (CO), 219.4 (CO), 163.6 (C_4/C_2), 151.3 (C_q Ar), 147.3 (C_q Ar), 142.8 (C_q Ar), 141.8 (C_q Ar), 140.6 (C_q Ar), 140.1 (C_q Ar), 139.2 (C_{18}), 130.3 (C_q Ar), 129.5 (C_q Ar), 128.3 (CH Ar) 127.8 (CH Ar), 127.5 (CH Ar), 126.6 (CH Ar), 125.8 (CH Ar), 124.2 (CH Ar), 124.0 (CH Ar), 123.9 (C_{16}), 123.7 (CH Ar), 123.4 (CH Ar), 83.8 (C_{15}), 64.4 (CH_2 of Bn), 40.4 (NMe_3), 28.2 (CH ^{i}Pr), 28.0 (CH ^{i}Pr), 27.8 (CH ^{i}Pr), 27.6 (CH ^{i}Pr), 26.0 (CH_3 ^{i}Pr), 25.6 (CH_3 ^{i}Pr), 25.1 (CH_3 ^{i}Pr), 25.0 (CH_3 ^{i}Pr), 24.9 (CH_3 ^{i}Pr), 24.7 (CH_3 ^{i}Pr), 24.4 (CH_3 ^{i}Pr), 24.3 (CH_3 ^{i}Pr), 14.8 (C_3 + C_{17}).

Placing samples of **13** under vacuum for longer than one minute or storing dried samples over longer than one day results in the formation of benzaldehyde, alongside unidentified iron-containing residues. The presence of benzaldehyde was also confirmed by GC/MS analysis.

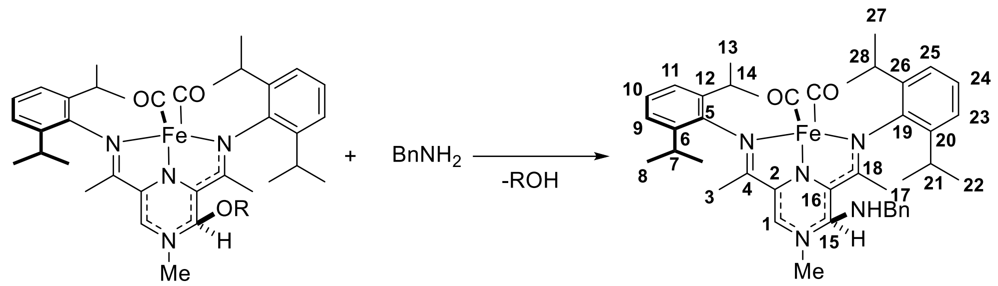

[(Me)(P^{pz}DI)(BnNH$_2$)Fe(CO)$_2$] (14). In a J-Young NMR tube, a solution of **11** (18 mg, 0.028 mmol) in C_6D_6 (0.6 mL) was treated with $BnNH_2$ (2 eq) (6.2 µL, 0.056 mmol). The NMR tube was quickly shaken and placed in an NMR spectrometer. A conversion of 66% to **14**, alongside unreacted starting material and methanol was observed. The conversion did not improve upon further the addition of 2eq $BnNH_2$. Data for **14**:

^{1}H NMR (400 MHz, C_6D_6) δ 7.35 – 6.95 (m, Bn + C_9H - C_{11}H + C_{23}H- C_{25}H, 11H), 6.31 (s, C_1H, 1H), 5.04 (d, J = 5Hz, 1H, C_{15}H), 3.61 – 3.15 (m, 7H, overlapping four hept x CH ^{i}Pr, one t NH, one AB spin system CH_2 of Bn) 2.24 (s, 3H, NMe), 1.74 (NH, 1H, J = 4.5 Hz, br q) 1.66 (s, 3H, C_{17}H), 1.58 (s, 3H, C_3H), 1.56 (d, 3H, J = 6.8 Hz, CH_3 ^{i}Pr), 1.53 (d, 3H, J = 6.8 Hz, CH_3 ^{i}Pr) 1.46 (app t, J = 6.8 Hz , 6H, CH_3 ^{i}Pr), 1.26 (d, 3H, J = 6.8 Hz, CH_3 ^{i}Pr), 1.21 (d, 3H, J = 6.8 Hz ,CH_3 ^{i}Pr), 1.09 (d, 3H, J = 6.8 Hz, CH_3 ^{i}Pr) 1.04 (d, 3H, J = 6.8 Hz, CH_3 ^{i}Pr) **^{13}C{^{1}H} NMR** (101 MHz, C_6D_6) δ 220.4 (CO), 219.8 (CO), 163.2 (C4/C2), 151.7 (C_q Ar), 147.3 (C_q Ar), 144.1 (C_{18}), 143.0 (C_q Ar), 142.0 (C_q Ar), 140.9 (C_q Ar), 140.8 (C_q Ar), 140.3 (C_q Ar), 131.1 (C_1), 129.2 (C_q Ar), 127.3 (CH Ar), 126.7 (CH Ar), 126.4 (CH Ar), 125.6 (CH Ar), 125.2 (CH Ar), 124.1 (CH Ar), 123.8 (CH Ar), 123.6 (C_{16}), 123.3 (CH Ar), 70.4 (C_{15}), 46.4 (CH_2 of Bn), 39.8 (NMe_3), 28.2 (CH ^{i}Pr), 28.0 (CH ^{i}Pr), 27.8 (CH ^{i}Pr), 27.6 (CH ^{i}Pr), 26.1 (CH_3 ^{i}Pr), 25.7 (CH_3 ^{i}Pr), 25.2 (CH_3 ^{i}Pr), 25.1 (CH_3 ^{i}Pr), 24.8 (CH_3 ^{i}Pr), 24.7 (CH_3 ^{i}Pr), 24.4 (CH_3 ^{i}Pr), 24.3 (CH_3 ^{i}Pr), 15.1 (C_3), 14.6 (C_{17}). **^{15}N – ^{1}H HMBC** (61 MHz, C_6D_6) 231.3 (N-Fe, cross peak to δ_H 6.32 and 5.04), 227.0 (C_4=N, cross peak to δ_H 1.58), 188.7 (C_{18}=N, cross peak to δ_H 1.66), 97.4 (NMe, cross peak to δ_H 6.29 and 2.25), δ 62.1 (BnNH cross peak to δ_H 5.04).

Postulated mechanism for stoichiometric alcohol dehydrogenation

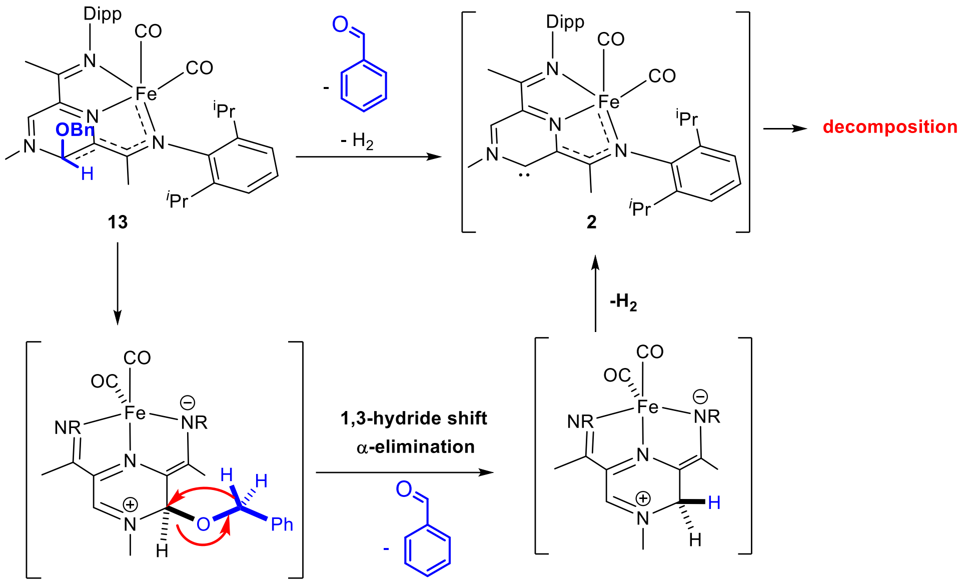

Scheme S1. Proposed mechanism for the alcohol dehydrogenation reaction

Additional attempted reactions

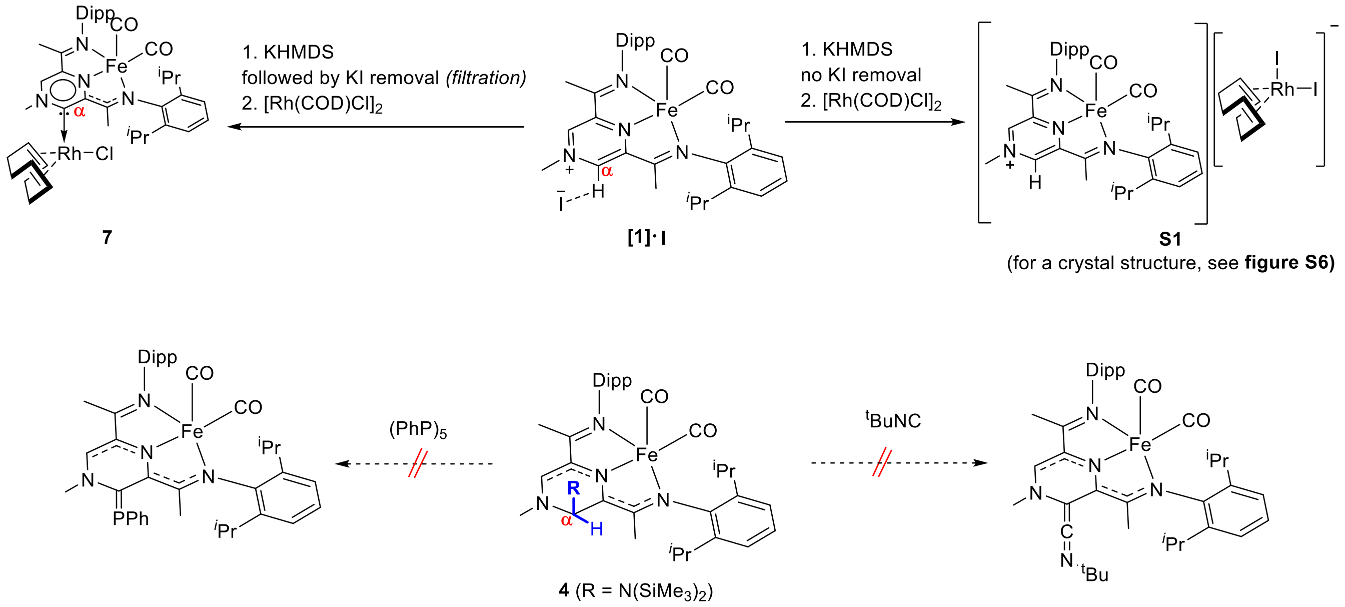

Scheme S2. Additional reactivity studies

Electronic Properties comparison with standard NHCs

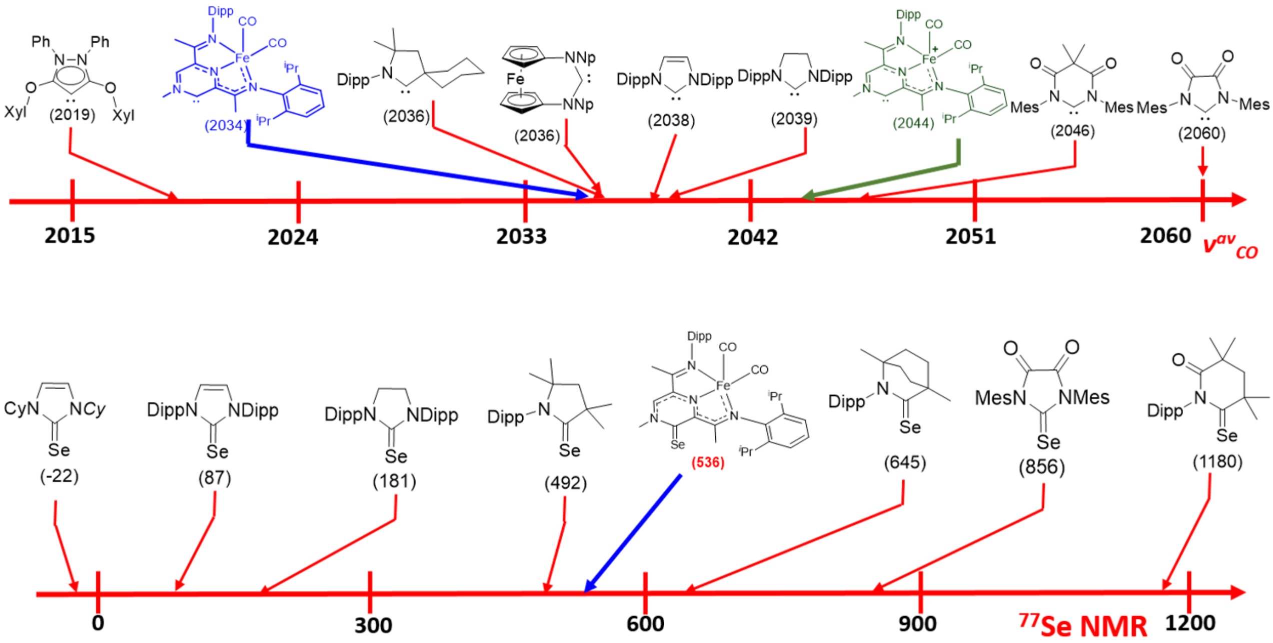

Figure S1. Comparison of the net overall donating properties of **2** through the ν^{av}_{CO} stretching frequencies (cm^{-1}) of the corresponding $Rh(CO)_2Cl$ complexes (in CH_2Cl_2 solutions) (above). Comparison of π-accepting properties of **2** through ^{77}Se NMR spectroscopy (in acetone-d_6) of the corresponding selenourea derivatives (below). The comparison values are taken from the literature.[9]

[9] (a) H. V. Huynh *Chem. Rev.* **2018**, *118*, 9457; (b) C. M. Weinstein, G. P. Junor, D. R. Tolentino, R. Jazzar, M. Melaimi, G. Bertrand *J. Am. Chem. Soc.* **2018**, *140*, 9255.

Supporting Crystallographic Information

X-ray Crystal Structure Determinations

Crystal data and details of the structure determinations are compiled in Table S1 – S3. Full shells of intensity data were collected at low temperature with an Agilent Technologies Supernova-E CCD diffractometer (Mo- or Cu-K_α radiation, microfocus X-ray tubes, multilayer mirror optics). Detector frames (typically ω-, occasionally φ-scans, scan width 0.4...1°) were integrated by profile fitting.[10,11] Data were corrected for air and detector absorption, Lorentz and polarization effects[11] and scaled essentially by application of appropriate spherical harmonic functions.[11,12,13] Absorption by the crystal was treated numerically (Gaussian grid).[13,14] An illumination correction was performed.[13]

The structures were solved by the heavy atom method combined with structure expansion by direct methods applied to difference structure factors[15] (complexes **8** and **11**) or by the charge flip procedure[16] (all other complexes) and refined by full-matrix least squares methods based on F^2 against all unique reflections.[17] All non-hydrogen atoms were given anisotropic displacement parameters. Hydrogen atoms were input at calculated positions and refined with a riding model.[18] Disorder of some of the CF_3 groups of the $BArF_{24}$ anion in **10**·0.5 *n*-pentane was treated with split atom models. Suitable geometry and adp constraints/restraints were applied.[18,19] The non-centrosymmetric structure of **S1**·*solv.* was refined as an inversion twin.

---

[10] K. Kabsch, in: M. G. Rossmann, E. Arnold (eds.), *"International Tables for Crystallography" Vol. F*, Ch. 11.3, Kluwer Academic Publishers, Dordrecht, The Netherlands, **2001**.

[11] *CrysAlisPro*, Agilent Technologies UK Ltd., Oxford, UK **2011-2014** and Rigaku Oxford Diffraction, Rigaku Polska Sp.z o.o., Wrocław, Poland **2015-2019**.

[12] R. H. Blessing, *Acta Cryst.* **1995**, *A51*, 33.

[13] *SCALE3 ABSPACK, CrysAlisPro*, Agilent Technologies UK Ltd., Oxford, UK **2011-2014** and Rigaku Oxford Diffraction, Rigaku Polska Sp.z o.o., Wrocław, Poland **2015-2019**.

[14] W. R. Busing, H. A. Levy, *Acta Cryst.* **1957**, *10*, 180.

[15] (a) P. T. Beurskens, G. Beurskens, R. de Gelder, J. M. M. Smits, S. Garcia-Granda, R. O. Gould, *DIRDIF-2008*, Radboud University Nijmegen, The Netherlands, **2008**; (b) P. T. Beurskens, in: G. M. Sheldrick, C. Krüger, R. Goddard (eds.), *Crystallographic Computing 3*, Clarendon Press, Oxford, UK, **1985**, p. 216.

[16] (a) L. Palatinus, *SUPERFLIP*, EPF Lausanne, Switzerland and Fyzikální ústav AV ČR, v. v. i., Prague, Czech Republic, **2007-2014**; (b) L. Palatinus, G. Chapuis, *J. Appl. Cryst.* **2007**, 40, 786.

[17] (a) G. M. Sheldrick, *SHELXL-20xx*, University of Göttingen and Bruker AXS GmbH, Karlsruhe, Germany **2012-2018**; (b) W. Robinson, G. M. Sheldrick in: N. W. Isaaks, M. R. Taylor (eds.) „*Crystallographic Computing 4*“, Ch. 22, IUCr and Oxford University Press, Oxford, UK, **1988**; (c) G. M. Sheldrick, *Acta Cryst.* **2008**, *A64*, 112; (d) G. M. Sheldrick, *Acta Cryst.* **2015**, *C71*, 3.

[18] (a) J. S. Rollett in: F. R. Ahmed, S. R. Hall, C. P. Huber (eds.) „*Crystallographic Computing*“ p. 167, Munksgaard, Copenhagen, Denmark, **1970**; (b) D. Watkin in: N. W. Isaaks, M. R. Taylor (eds.) „*Crystallographic Computing 4*“, Ch. 8, IUCr and Oxford University Press, Oxford, UK, **1988**; (c) P. Müller, R. Herbst-Irmer, A. L. Spek, T. R. Schneider, M. R. Sawaya in: P. Müller (ed.) *"Crystal Structure Refinement"*, Ch. 5, Oxford University Press, Oxford, UK, **2006**; (d) D. Watkin, *J. Appl. Cryst.* **2008**, *41*, 491.

[19] A. Thorn, B. Dittrich, G. M. Sheldrick, *Acta Cryst.* **2012**, *A68*, 448.

Due to severe disorder, electron density attributed to solvent of crystallization was removed from the structures of **7** (presumably *n*-pentane), **10** (*n*-pentane) and **S1** (*n*-pentane and/or thf) with the BYPASS procedure,[20] as implemented in PLATON (squeeze/hybrid).[21] Partial structure factors from the solvent masks were included in the refinement as separate contributions to F_{calc}.

Crystals of **8** were of bad quality. Intensity data could be collected from one specimen to an effective resolution of about 0.95 Å. The structure could be solved but not be refined satisfactorily, due to the low number of observed reflections. We chose to limit discussion to atomic connectivity only, which was established unambiguously.

CCDC 1968503 and 1998354 - 1998356 contains the supplementary crystallographic data for this paper. These data can be obtained free of charge from the Cambridge Crystallographic Data Centre's and FIZ Karlsruhe's joint Access Service via https://www.ccdc.cam.ac.uk/structures/?.

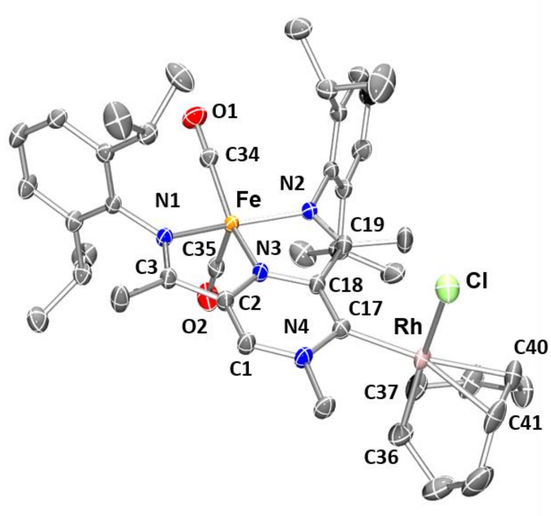

Figure S2. Molecular structure of **7** with displacement ellipsoids drawn at 50% probability. Hydrogen atoms are omitted for clarity. Selected bond distances [Å] and angles [deg]: Fe–C34 1.8018(16), Fe–C35 1.755(17), Fe–N1 1.9824(13), Fe–N2 1.9563(13), Fe–N3 1.8256(13), N1–C3 1.3194(19), C3–C2 1.434(2), C2–N3 1.374(2), N3–C18 1.3909(19), C18–C19 1.433(2), C19–N2 1.330(2), C18-C17 1.402(2), C17-N4 1.389(2), C17-Rh 2.0218(6), C35–Fe–C34 94.70(8), C2—C1—N4 119.74(15), C18—C17—N4 114.29(14).

[20] P. v. d. Sluis, A. L. Spek, *Acta Cryst.* **1990**, *A46*, 194; (b) A. L. Spek, *Acta Cryst.* **2015**, *C71*, 9.
[21] A. L. Spek, *PLATON*, Utrecht University, The Netherlands; (b) A. L. Spek, *J. Appl. Cryst.* **2003**, *36*, 7.

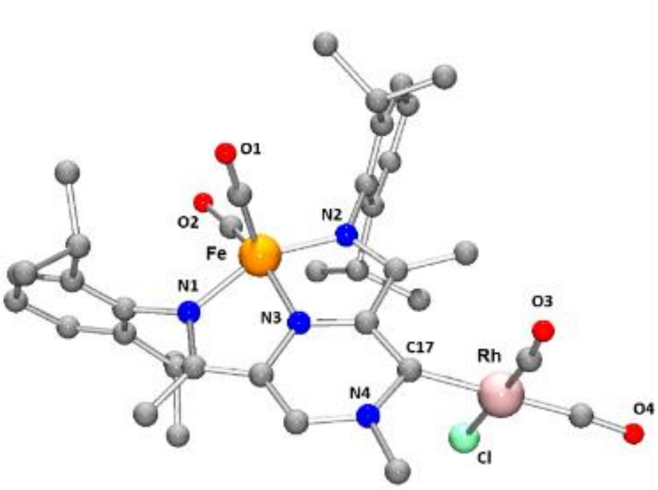

Figure S3. Ball-and-stick molecular structure of **8.** Hydrogen atoms are omitted for clarity.

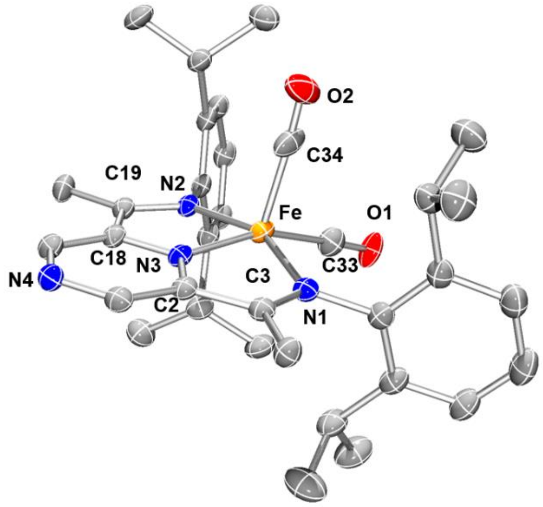

Figure S4. Molecular structure of **10** with displacement ellipsoids drawn at 30% probability. Hydrogen atoms and the B(3,5-CF_3-C_6H_3)$_4$ anion are omitted for clarity. Selected bond distances [Å] and angles [deg]: Fe–C33 1.799(4), Fe–C34 1.801(5), Fe–N1 1.992(3), Fe–N2 1.989(3), Fe–N3 1.836(3), N1–C3 1.313(4), C3–C2 1.438(5), C2–N3 1.379(4), N3–C18 1.375(4), C18–C19 1.443(5), C19–N2 1.311(4), C33–Fe–C34 94.4(2) , N1–Fe–C33 98.55(15), N1–Fe–C34 99.15(14).

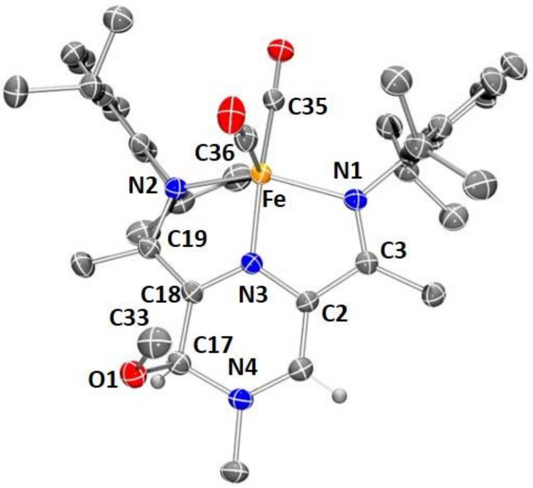

Figure S5. Molecular structure of **11** with displacement ellipsoids drawn at 50% probability. Hydrogen atoms are omitted for clarity. Selected bond distances [Å] and angles [deg]: Fe–C35 1.770(4), Fe–C36 1.752(4), Fe–N1 2.004(3), Fe–N2 1.935(3), Fe–N3 1.845(3), N1–C3 1.320(4), C3–C2 1.426(4), C2–N3 1.389(4), N3–C18 1.360(4), C18–C19 1.391(4), C19–N2 1.361(4), C35–Fe–C36 95.41(17) , N1–Fe–C35 97.89(13), N1–Fe–C36 99.10(13).

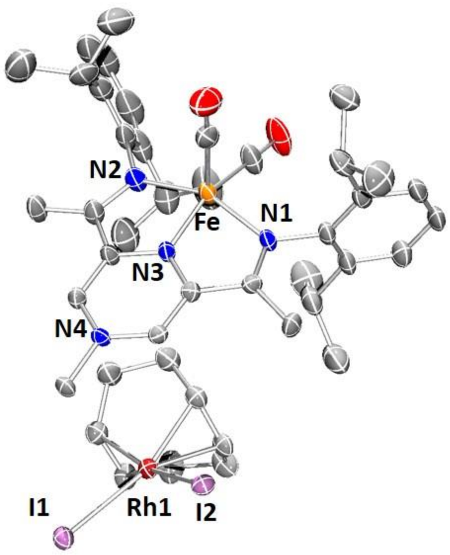

Figure S6. Molecular structure of **S1** with displacement ellipsoids drawn at 50% probability. Hydrogen atoms are omitted for clarity. Selected bond distances [Å] and angles [deg]:]: Fe–C34 1.792(5), Fe–C33 1.809(5), Fe–N1 1.964(3), Fe–N2 1.981(3), Fe–N3 1.817(3), N1–C3 1.330(5), C3–C2 1.444(5), C2–N3 1.375(5), C18–C19 1.429(5), C33-Fe-C34 95.8(2).

Table S1. Details of crystal structure determinations of **7**, **8**, **10** and **S1.**

	**7**·*x n*-pentane	**8**
formula	$C_{43}H_{56}ClFeN_4O_2Rh$	$C_{37}H_{44}ClFeN_4O_4Rh$
crystal system	monoclinic	triclinic
space group	*C* 2/*c*	*P* -1
a /Å	35.8785(3)	11.8086(3)
b /Å	12.15433(11)	12.6461(3)
c /Å	20.8985(2)	12.9526(3)
α /°		102.663(2)
β /°	95.3430(8)	93.753(2)
γ /°		99.771(2)
V /Å^3	9073.80(14)	1849.17(8)
Z	8	2
M_r	855.12	802.97
F_{000}	3568	828
d_c /Mg·m^{-3}	1.252	1.442
μ /mm^{-1}	0.779	16.455
max., min. transmission factors	1.000, 0.627	
X-radiation, λ /Å	Mo *K*α, 0.71073	Cu *K*α, 1.54184
data collect. temperat. /K	120(1)	120(1)
θ range /°	2.3 to 34.2	3.5 to 67.8
index ranges *h*,*k*,*l*	-56 ... 56, -18 ... 19, -32 ... 32	-14 ... 14, -15 ... 14, -15 ... 15
reflections measured	149550	78259
unique [R_{int}]	18212 [0.0534]	6595 [0.2470]
observed [$I \geq 2\sigma(I)$]	14328	2206
data / restraints /parameters	18212 / 0 / 480	
GooF on F^2	1.041	
R indices [$F>4\sigma(F)$] $R(F)$, $wR(F^2)$	0.0399, 0.0948	
R indices (all data) $R(F)$, $wR(F^2)$	0.0569, 0.1018	
largest residual peaks /e·Å^{-3}	1.662, -0.544	
**CCDC deposition number**	**1998354**	

Table S2. Details of crystal structure determinations of **10**·0.5 *n*-pentane and **11**.

	**10**·0.5 *n*-pentane	**11**
formula	$C_{68.50}H_{60}BF_{24}FeN_4O_2$	$C_{36}H_{48}FeN_4O_3$
crystal system	monoclinic	monoclinic
space group	$P\,2_1/c$	$P\,2_1/n$
a /Å	17.40812(15)	10.4618(3)
b /Å	17.89181(17)	22.6514(7)
c /Å	22.5201(3)	14.4987(5)
β /°	106.1983(10)	98.319(3)
V /Å^3	6735.72(12)	3399.65(19)
Z	4	4
M_r	1493.86	640.63
F_{000}	3048	1368
d_c /Mg·m^{-3}	1.473	1.252
μ /mm^{-1}	2.820	3.868
max., min. transmission factors	1.000, 0.689	1.000, 0.715
X-radiation, λ /Å	Cu-$K\alpha$, 1.54184	Cu-$K\alpha$, 1.54184
data collect. temperat. /K	120(1)	120(1)
θ range /°	2.6 to 71.5 °	3.647 to 67.203
index ranges h,k,l	-21 ... 21, -21 ... 21, -27 ... 25	-12 ... 12, -27 ... 27, -17 ... 16
reflections measured	211144	89272
unique [R_{int}]	12978 [0.0493]	6082 [0.1577]
observed [$I \geq 2\sigma(I)$]	11172	4005
data / restraints /parameters	12978 / 270 / 949	6082 / 0 / 409
GooF on F^2	1.040	1.021
R indices [$F>4\sigma(F)$] $R(F)$, $wR(F^2)$	0.0709, 0.1927	0.0523, 0.1118
R indices (all data) $R(F)$, $wR(F^2)$	0.0802, 0.2001	0.0953, 0.1322
largest residual peaks /e·Å^{-3}	1.703, -1.117	0.234, -0.510
**CCDC deposition number**	**1998355**	**1968503**

Table S3. Details of crystal structure determinations of **S1**·*solv.*

	**S1**·*solv.*
formula	$C_{57}H_{89}FeI_2N_4O_3Rh$
crystal system	tetragonal
space group	P -4$2_1c$
a /Å	23.13493(14)
b /Å	23.13493(14)
c /Å	20.6820(2)
β /°	90
V /Å^3	11069.49(17)
Z	8
M_r	1290.88
F_{000}	5280
d_c /Mg·m^{-3}	1.549
μ /mm^{-1}	1.722
max., min. transmission factors	1.000, 0.785
X-radiation, λ /Å	Mo $K\alpha$, 0.71073
data collect. temperat. /K	120(1) K
θ range /°	2.2 to 34.2 °
index ranges h,k,l	-35 ... 36, -35 ... 36, -32 ... 32
reflections measured	500709
unique [R_{int}]	22666 [0.1307]
observed [$I \geq 2\sigma(I)$]	17437
data / restraints /parameters	22666 / 0 / 490
GooF on F^2	0.973
R indices [$F>4\sigma(F)$] $R(F)$, $wR(F^2)$	0.0374, 0.0631
R indices (all data) $R(F)$, $wR(F^2)$	0.0622, 0.0685
largest residual peaks /e·Å^{-3}	0.846, -0.381
**CCDC deposition number**	**1998356**

NMR Spectra

Table S4. Comparison of ^{15}N NMR chemical shifts of the selected compounds

Number	Compound	δ_{N1}	$\delta_{N2}{}^{a}$	δ_{N4}
**1 · [I]**	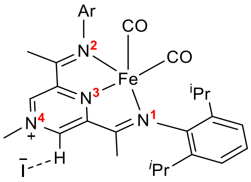 [1]·I	261.9	–	145.5
**4**	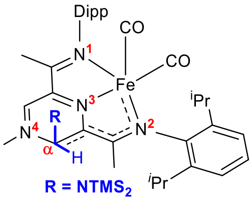	224.6	188.3	103.3
**6**	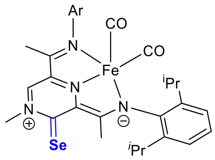	250.6	248.6	170.6
**7**	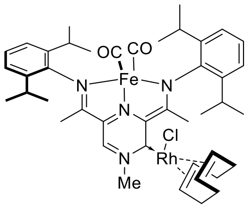	256.0	235.1	176.1
**8**	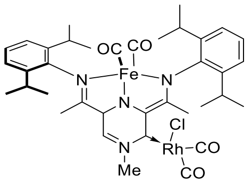	256.4	243.5	171.4
**11**	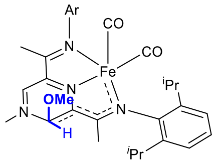	231.4	188.2	94.8

^{a} Displayed when $\delta_{N1} \neq \delta_{N2}$

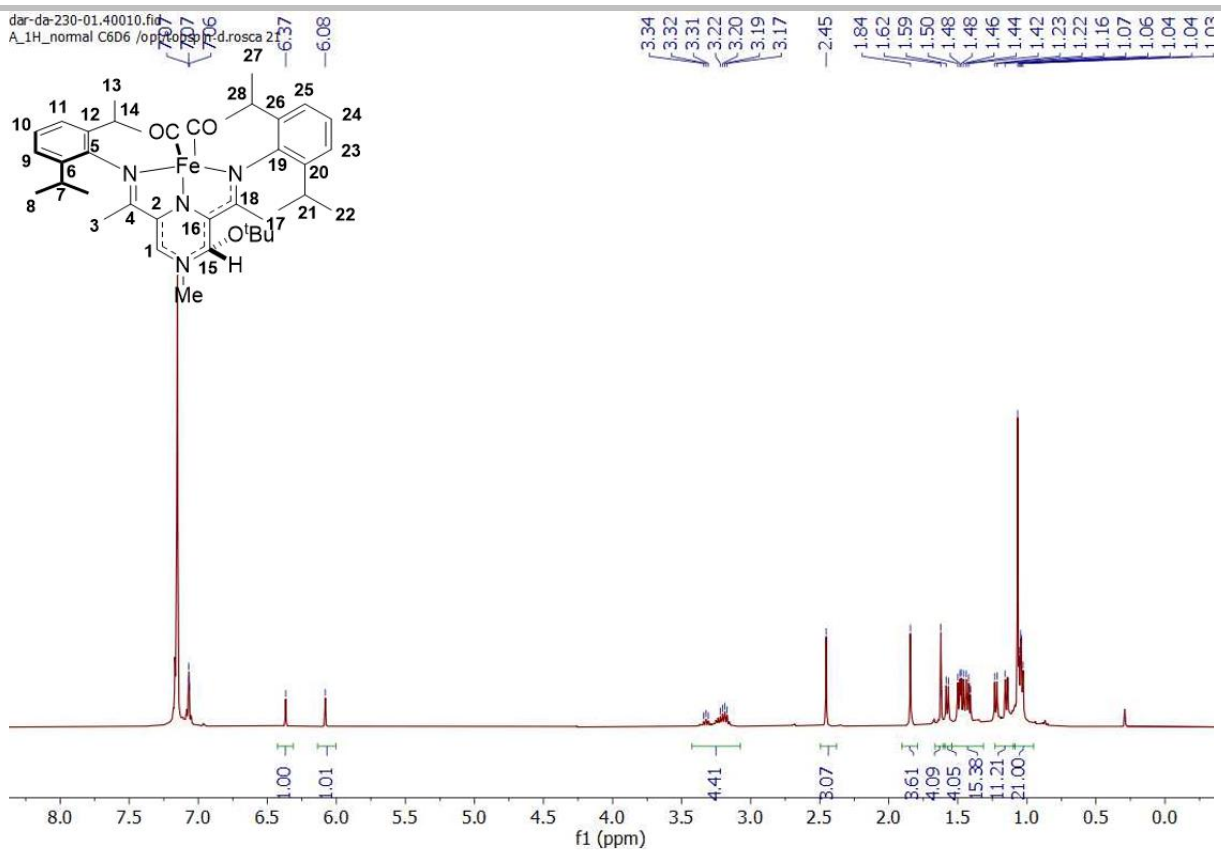
dar-da-230-01.40010.fid
A_1H_normal C6D6 /opt/topspin d.rosca 21
f1 (ppm)

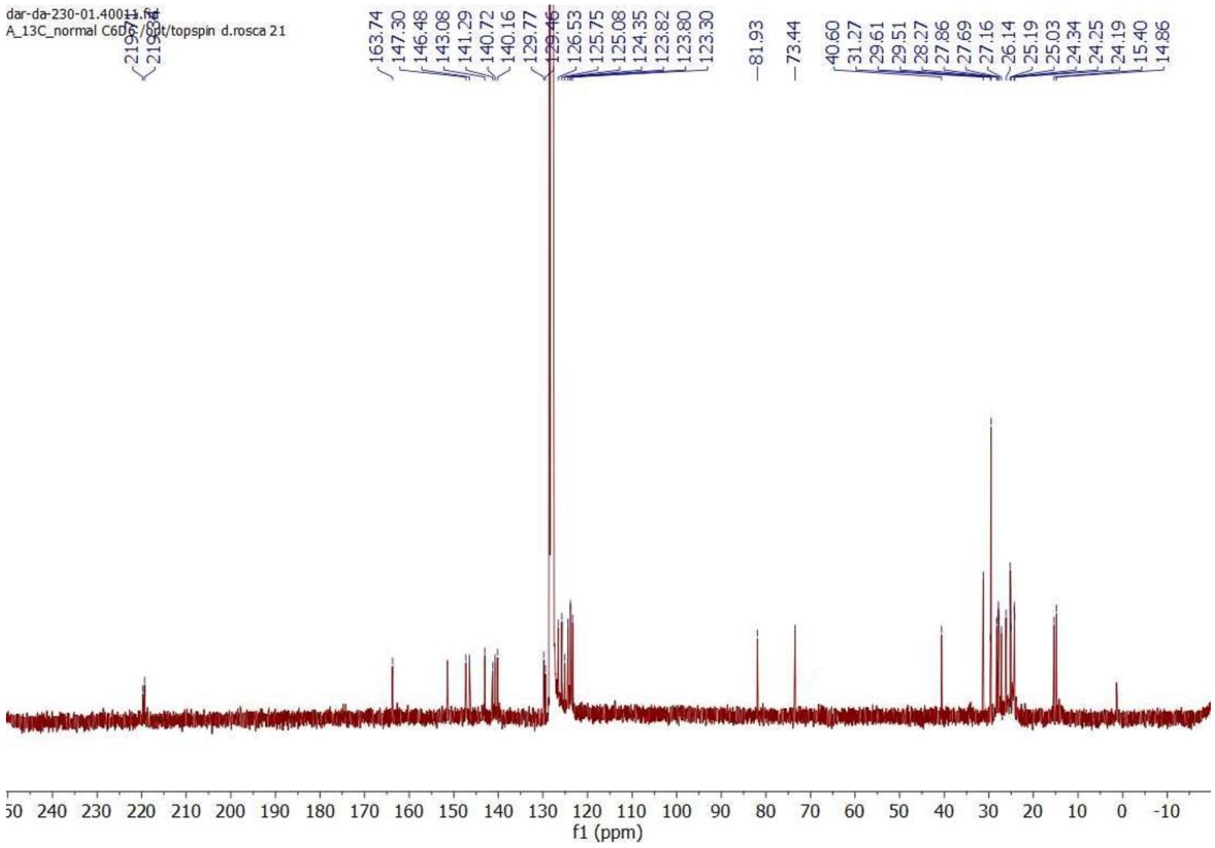
dar-da-230-01.40011.fid
A_13C_normal C6D6 /opt/topspin d.rosca 21
f1 (ppm)

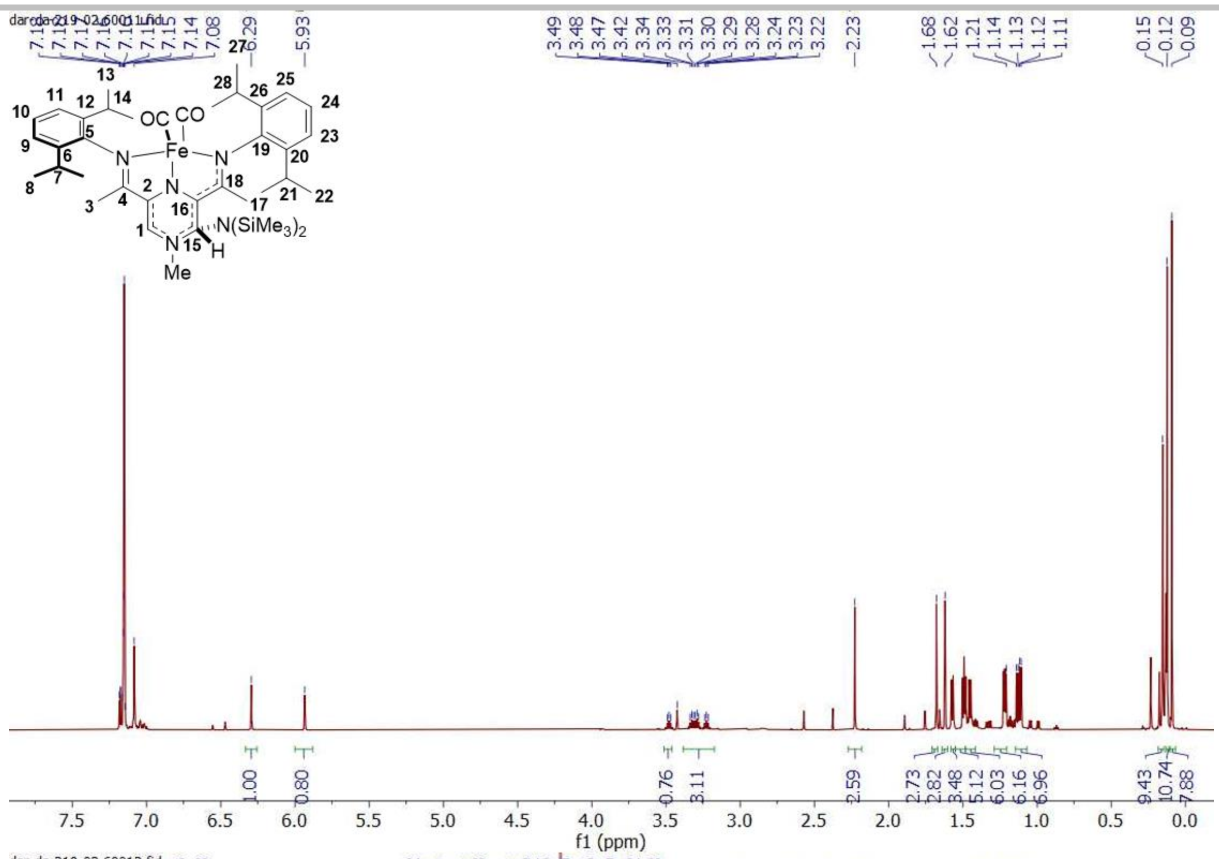
dar-da-219-02.60011.fid
6.29
5.93
3.49
3.48
3.47
3.42
3.34
3.33
3.31
3.30
3.29
3.28
3.24
3.23
3.22
2.23
1.68
1.62
1.21
1.14
1.13
1.12
1.11
0.15
0.12
0.09
OC
CO
Fe
N
N(SiMe3)2
H
Me
1.00
0.80
0.76
3.11
2.59
2.73
2.82
3.48
5.12
6.03
6.16
6.96
9.43
10.74
7.88
f1 (ppm)

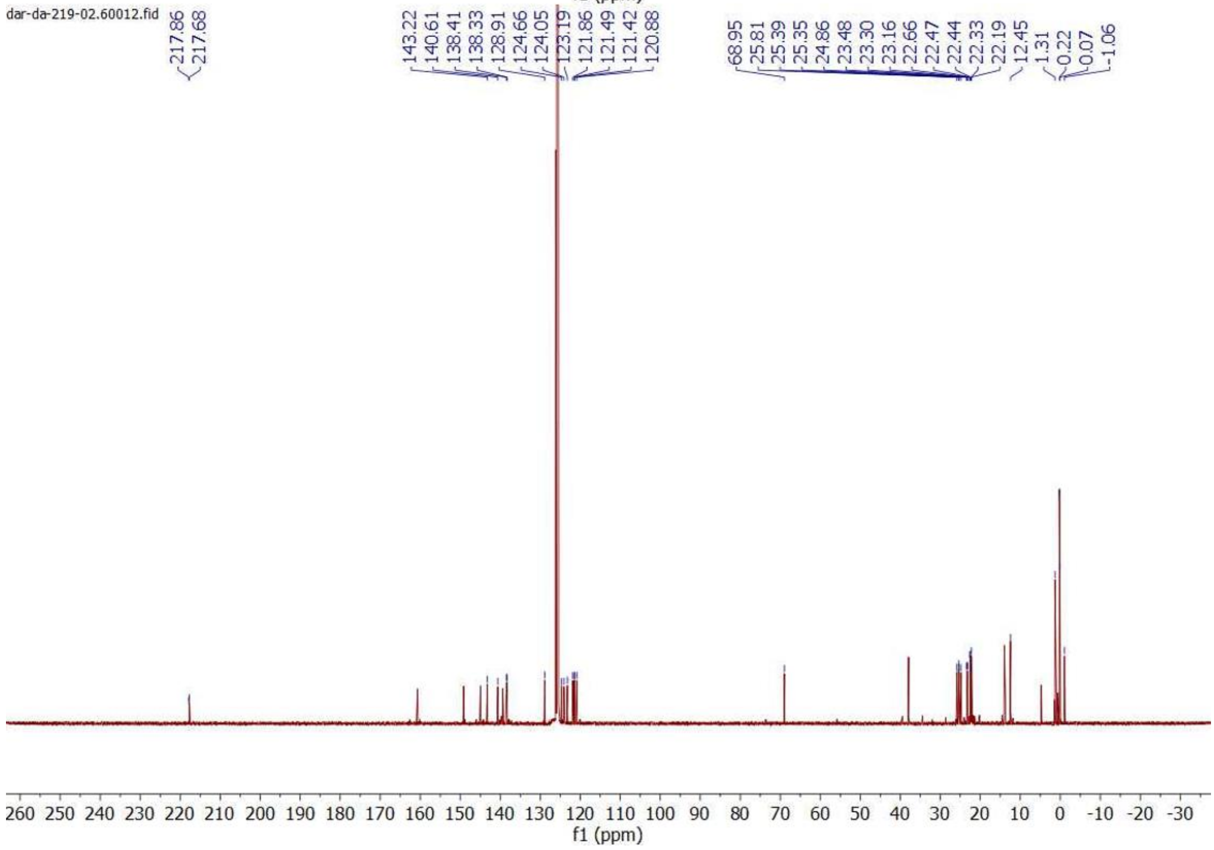
dar-da-219-02.60012.fid
217.86
217.68
143.22
140.61
138.41
138.33
128.91
124.66
124.05
123.19
121.86
121.49
121.42
120.88
68.95
25.81
25.39
25.35
24.86
23.48
23.30
23.16
22.66
22.47
22.44
22.33
22.19
12.45
1.31
0.22
0.07
-1.06
f1 (ppm)

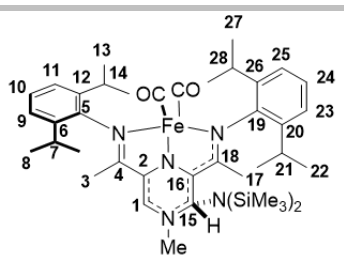
OC
CO
Fe
N
N(SiMe3)2
H
Me

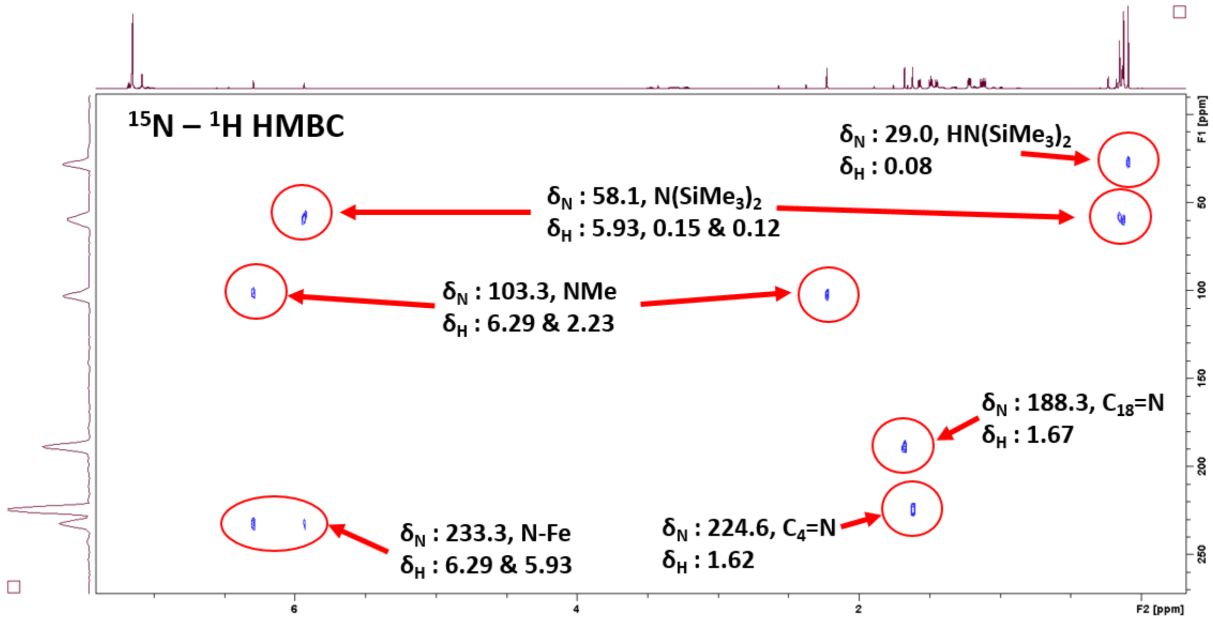
15N – 1H HMBC
δN : 29.0, HN(SiMe3)2
δH : 0.08
δN : 58.1, N(SiMe3)2
δH : 5.93, 0.15 & 0.12
δN : 103.3, NMe
δH : 6.29 & 2.23
δN : 188.3, C18=N
δH : 1.67
δN : 233.3, N-Fe
δH : 6.29 & 5.93
δN : 224.6, C4=N
δH : 1.62
F1 [ppm]
F2 [ppm]

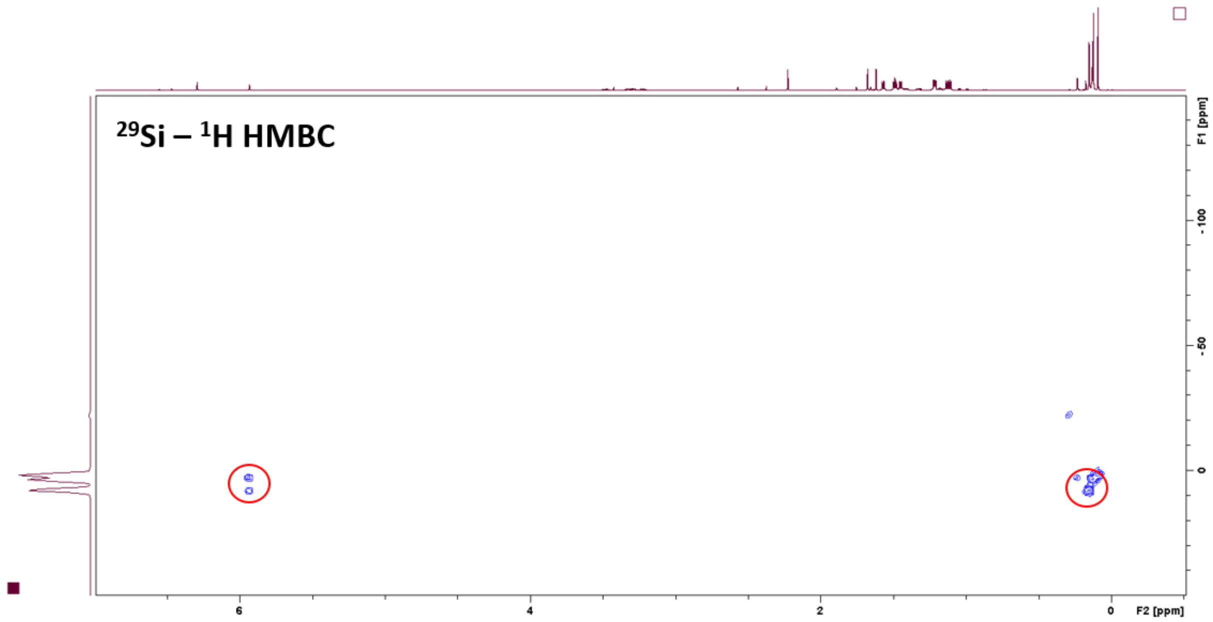
29Si – 1H HMBC
F1 [ppm]
F2 [ppm]

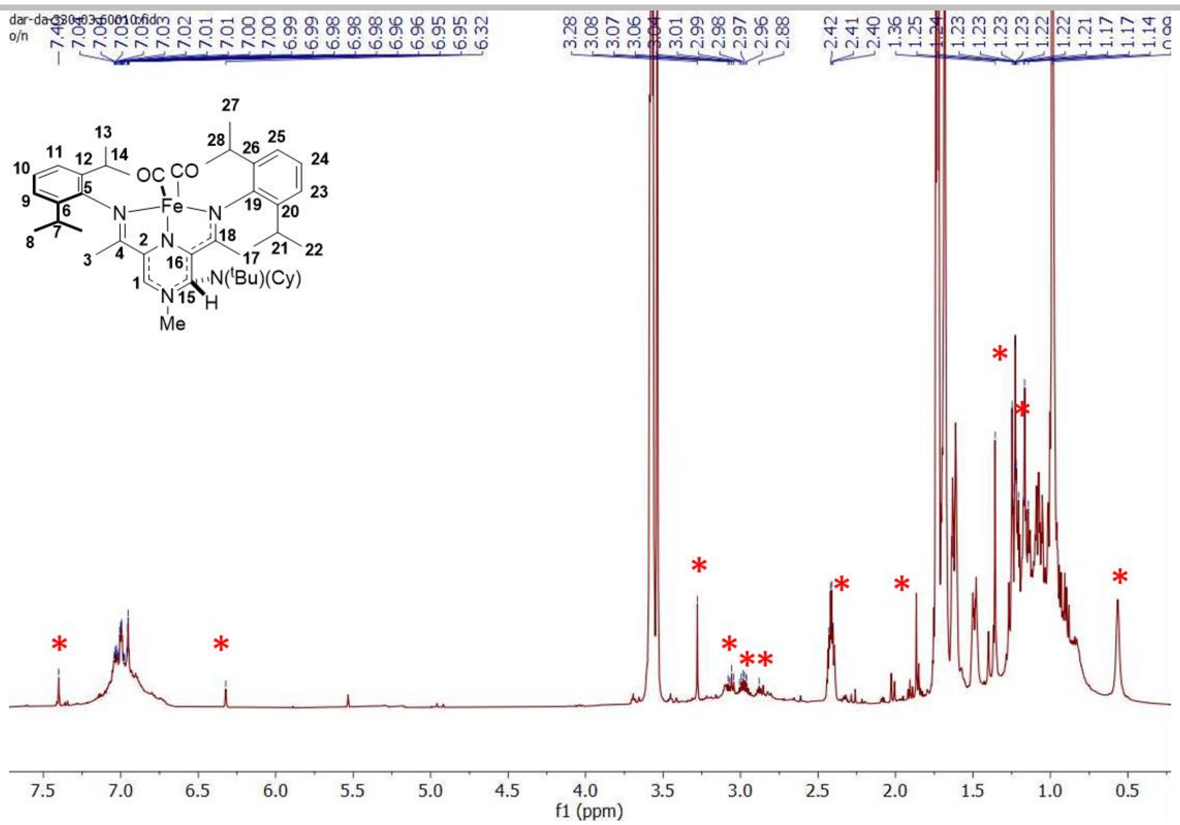

* Denotes signals belonging to the title complex

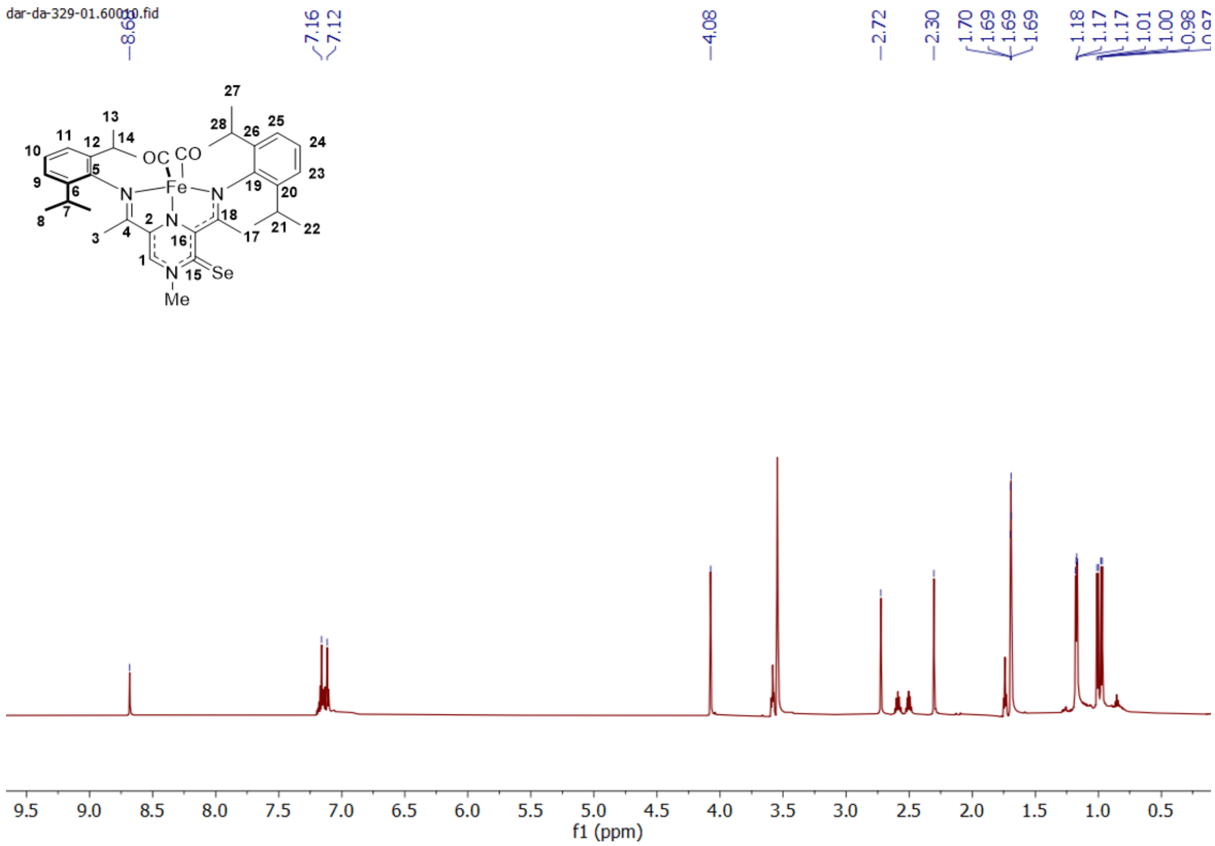
dar-da-329-01.60010.fid
8.69
7.16
7.12
4.08
2.72
2.30
1.70
1.69
1.69
1.69
1.18
1.17
1.17
1.01
1.00
0.98
0.97
OC
CO
Fe
N
Se
Me
f1 (ppm)

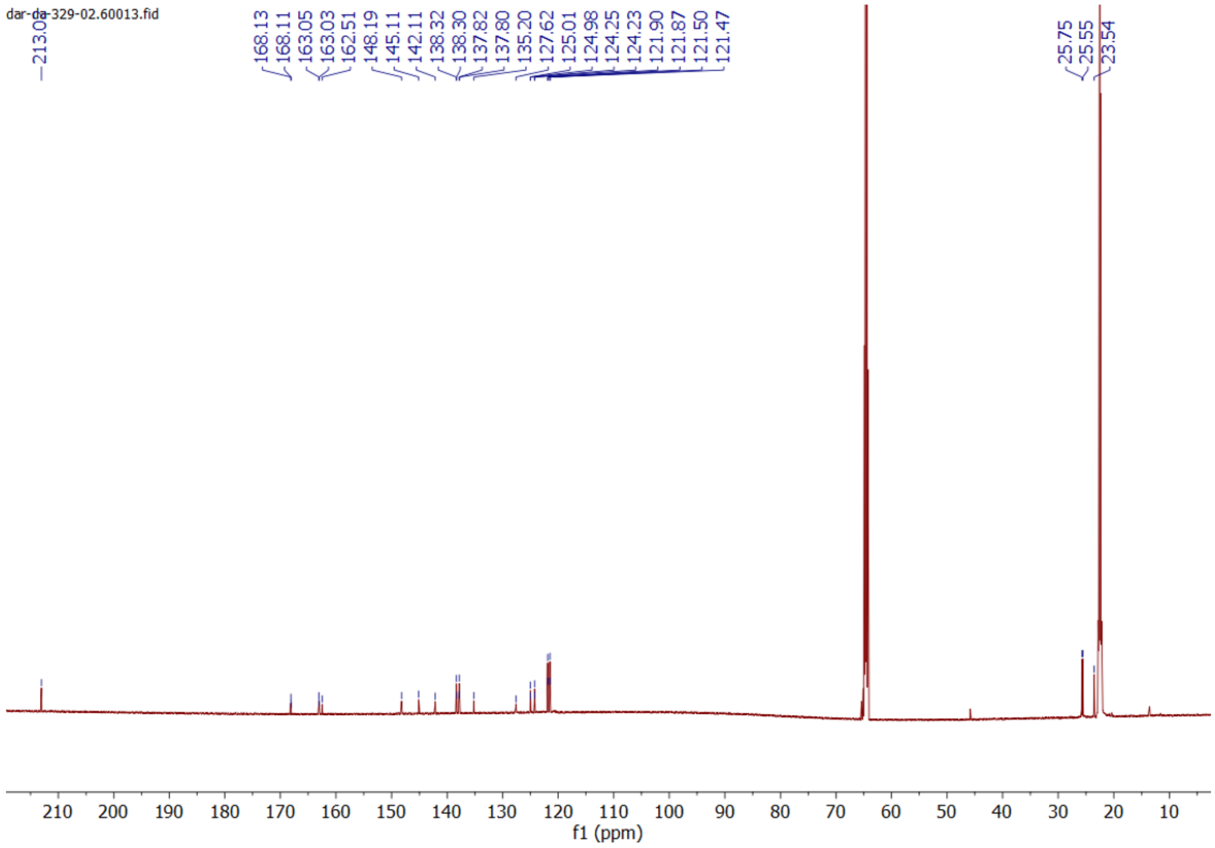
dar-da-329-02.60013.fid
213.09
168.13
168.11
163.05
163.03
162.51
148.19
145.11
142.11
138.32
138.30
137.82
137.80
135.20
127.62
125.01
124.98
124.25
124.23
121.90
121.87
121.50
121.47
25.75
25.55
23.54
f1 (ppm)

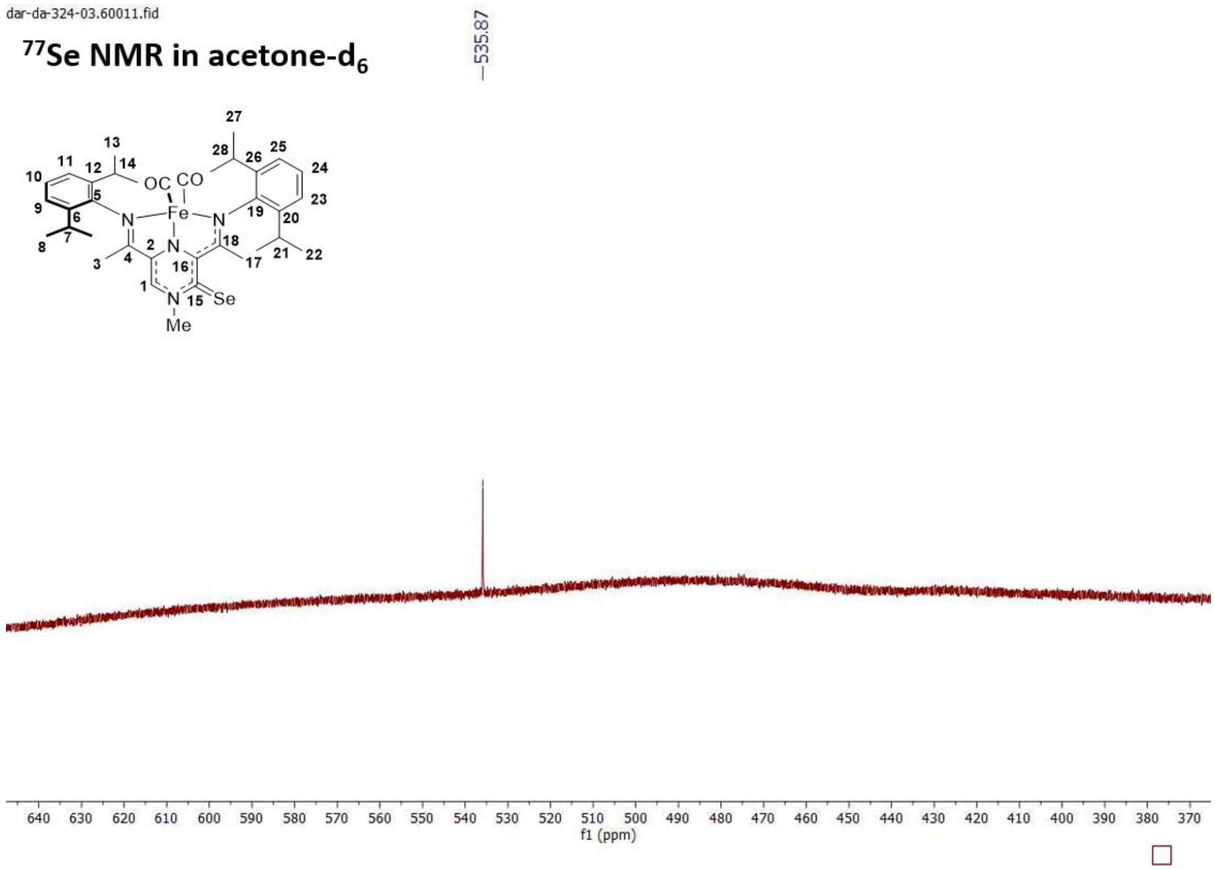
dar-da-324-03.60011.fid
^{77}Se NMR in acetone-d_6
—535.87
Fe
OC
CO
N
Se
Me
f1 (ppm)

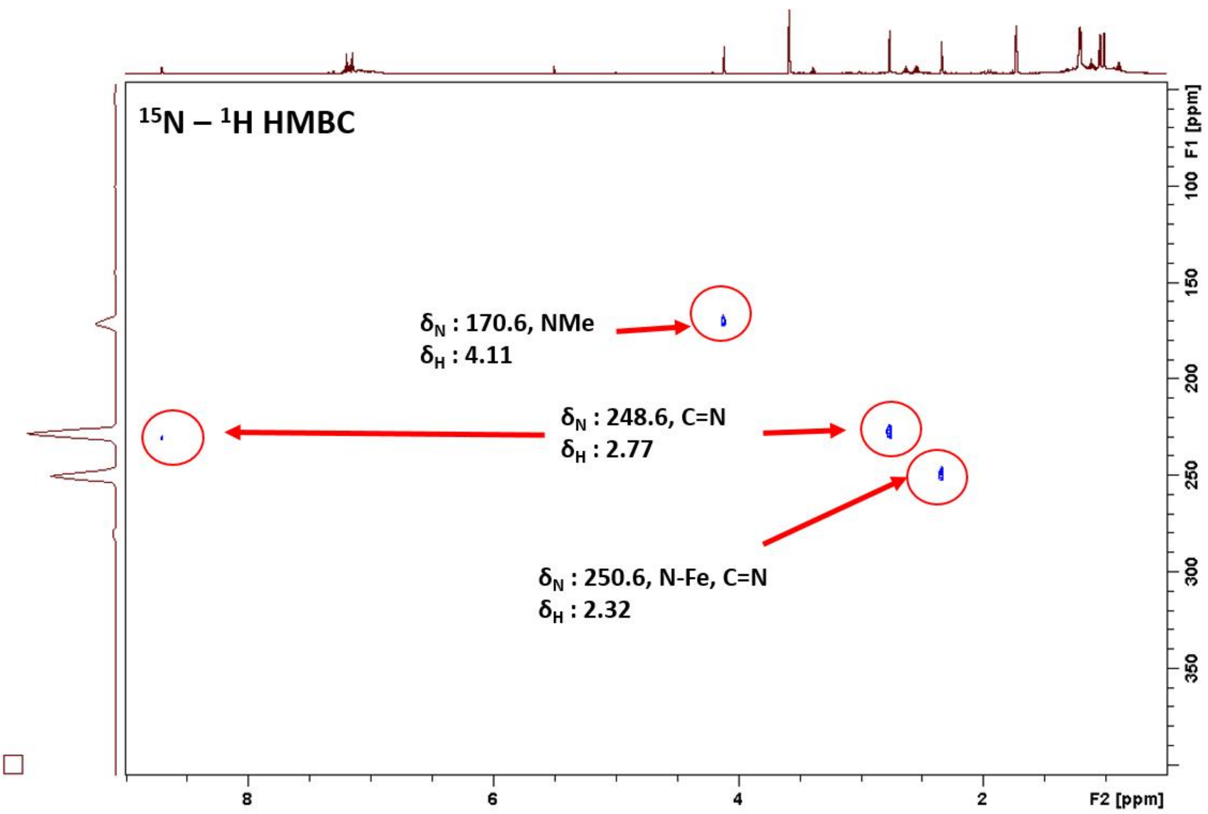
^{15}N – ^{1}H HMBC
δ_N : 170.6, NMe
δ_H : 4.11
δ_N : 248.6, C=N
δ_H : 2.77
δ_N : 250.6, N-Fe, C=N
δ_H : 2.32
F1 [ppm]
F2 [ppm]

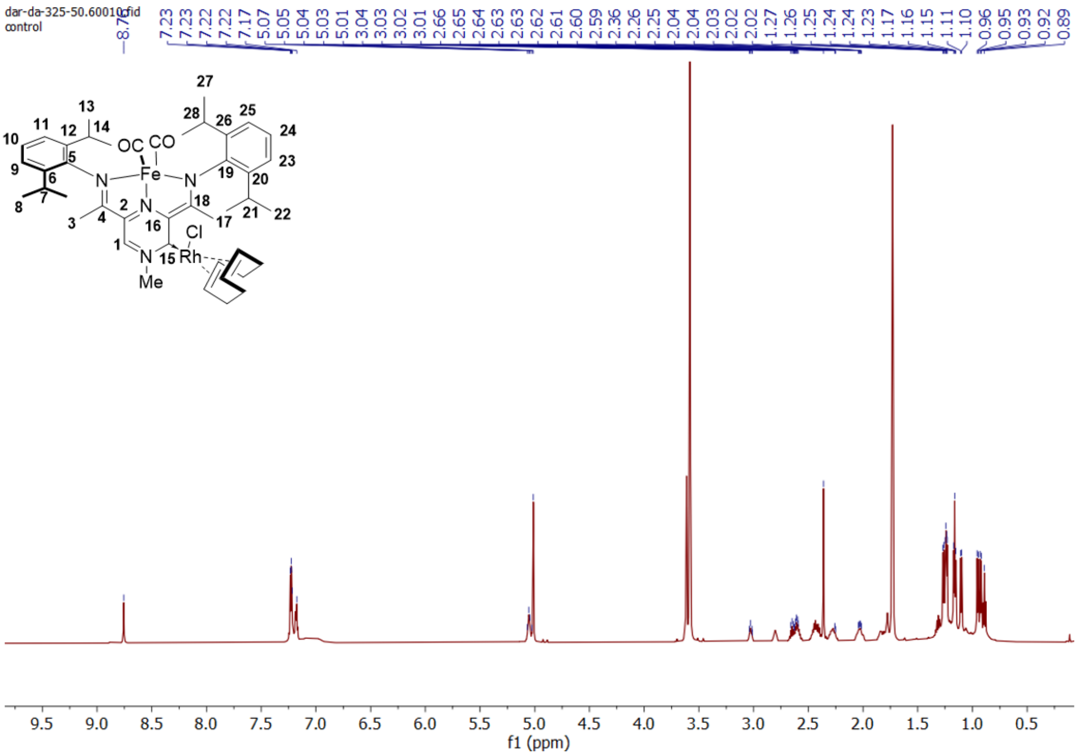
dar-da-325-50.60010.fid
control
7.23
7.23
7.22
7.22
7.17
5.07
5.05
5.04
5.03
5.01
3.04
3.03
3.02
3.01
2.66
2.65
2.64
2.63
2.63
2.62
2.61
2.60
2.59
2.36
2.26
2.25
2.04
2.04
2.03
2.02
2.02
1.27
1.26
1.25
1.24
1.24
1.23
1.17
1.16
1.15
1.11
1.10
0.96
0.95
0.93
0.92
0.89
OC
CO
Fe
N
Cl
Rh
Me
f1 (ppm)

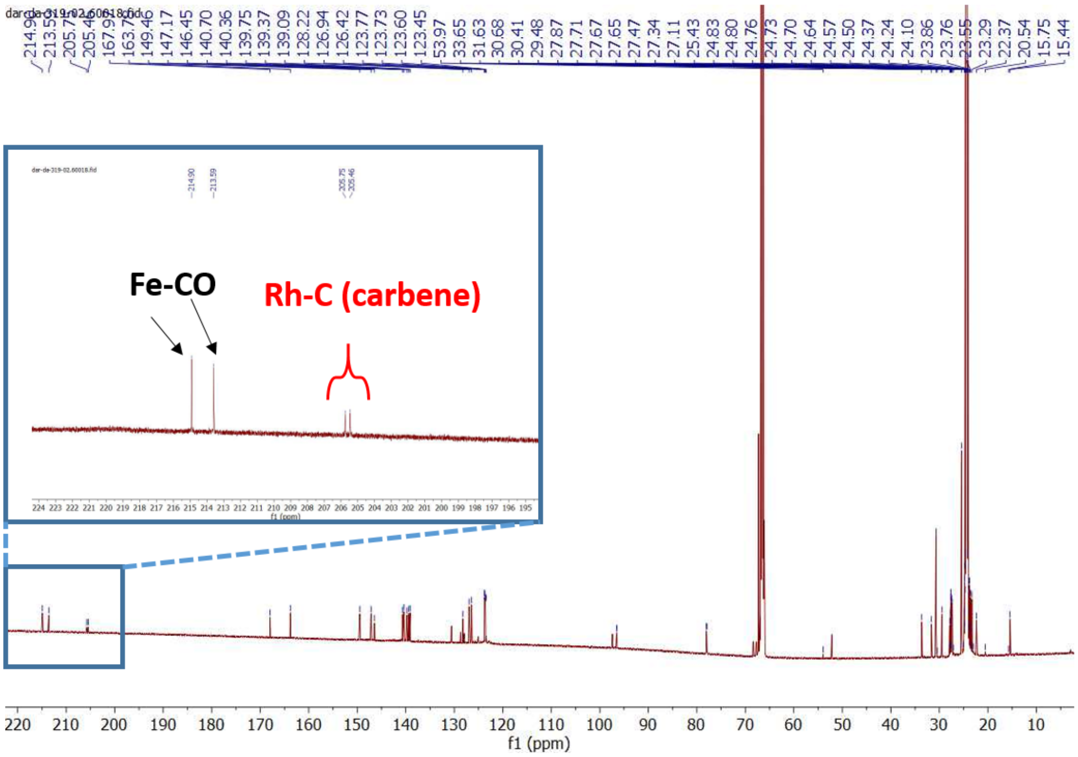
167.97
163.77
149.40
147.17
146.45
140.70
140.36
139.75
139.37
139.09
128.22
126.94
126.42
123.77
123.73
123.60
123.45
53.97
33.65
31.63
30.68
30.41
29.48
27.87
27.71
27.67
27.65
27.47
27.34
27.11
25.43
24.83
24.80
24.76
24.73
24.70
24.64
24.57
24.50
24.37
24.24
24.10
23.86
23.76
23.55
23.29
22.37
20.54
15.75
15.44
Fe-CO
Rh-C (carbene)
f1 (ppm)

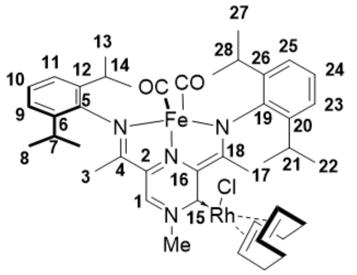
27
13
28
26
25
11
12
14
OC
CO
24
10
5
9
N
Fe
N
19
23
6
20
7
2
N
18
8
3
4
16
17
21
22
Cl
1
N
15
Rh
Me

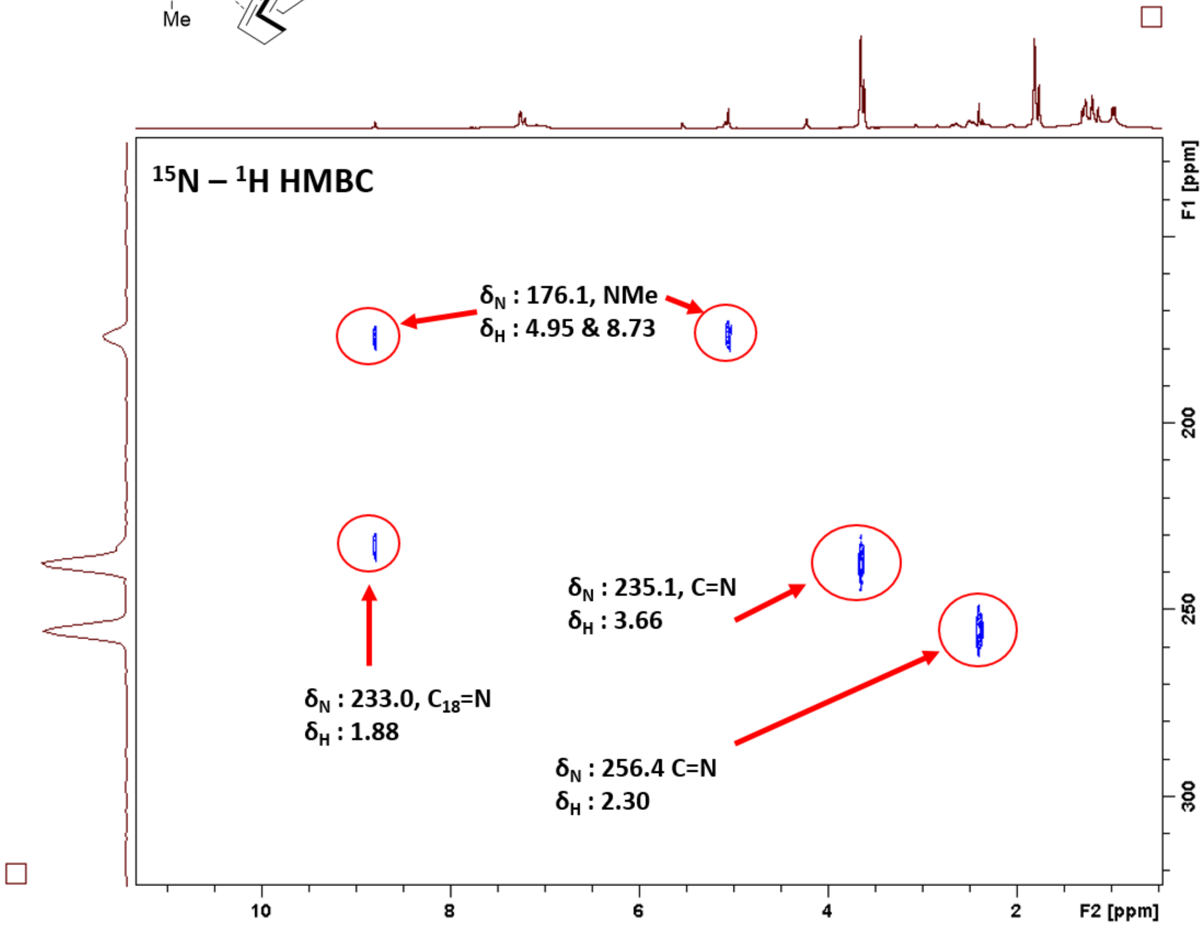
$^{15}N - ^{1}H$ HMBC
δ_N : 176.1, NMe
δ_H : 4.95 & 8.73
δ_N : 235.1, C=N
δ_H : 3.66
δ_N : 233.0, C_{18}=N
δ_H : 1.88
δ_N : 256.4 C=N
δ_H : 2.30
F1 [ppm]
200
250
300
10
8
6
4
2
F2 [ppm]

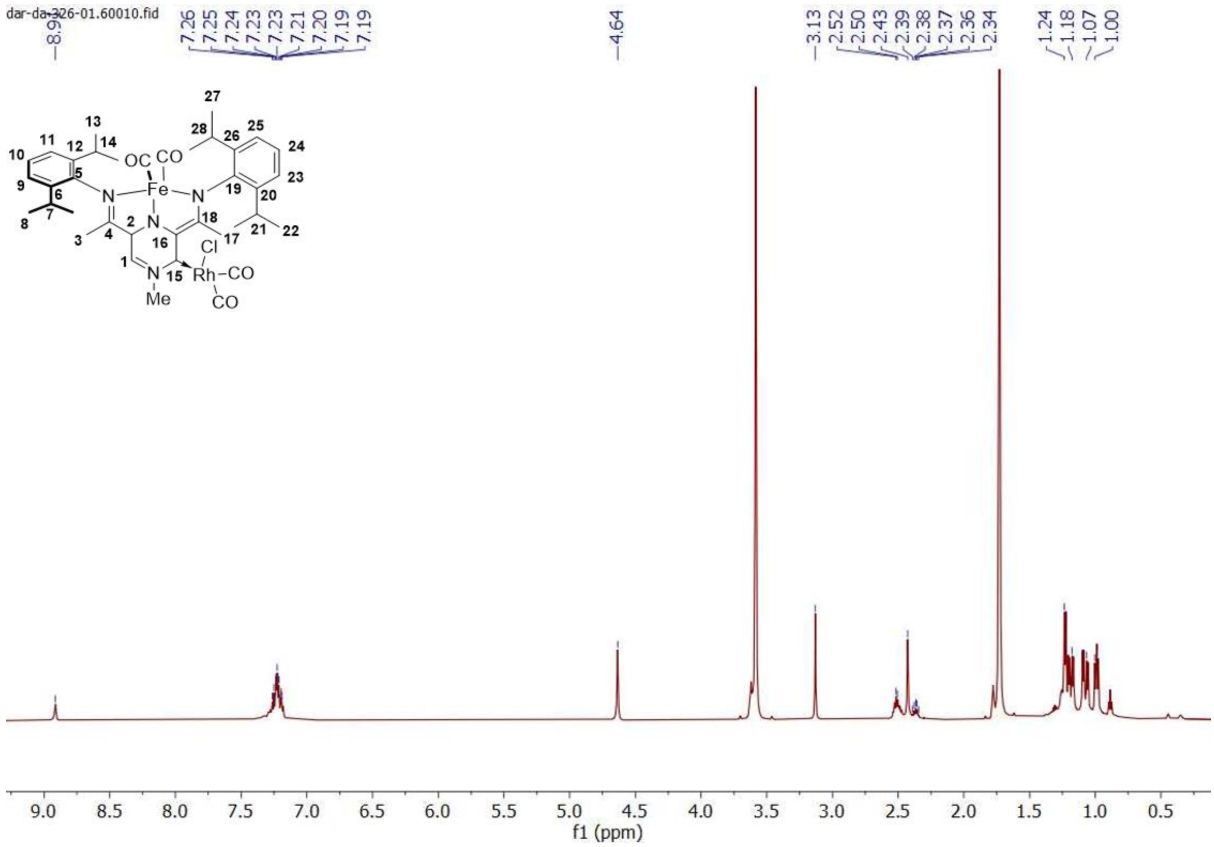
dar-da-326-01.60010.fid
8.91
7.26 7.25 7.24 7.23 7.23 7.21 7.20 7.19 7.19
4.64
3.13 2.52 2.50 2.43 2.39 2.38 2.37 2.36 2.34
1.24 1.18 1.07 1.00
OC CO
Fe
N
Cl
Rh–CO
CO
Me
f1 (ppm)

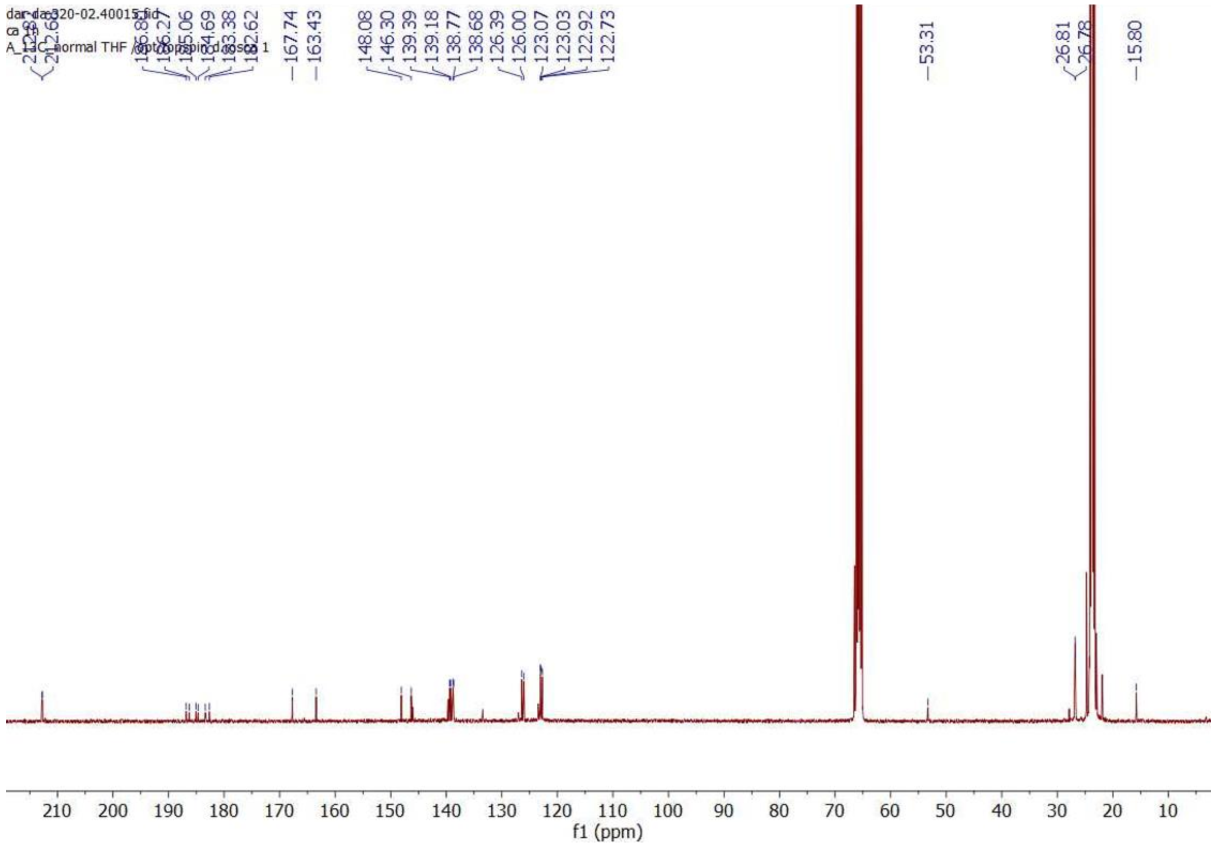
167.74
163.43
148.08 146.30 139.39 139.18 138.77 138.68 126.39 126.00 123.07 123.03 122.92 122.73
53.31
26.81 26.78
15.80
f1 (ppm)

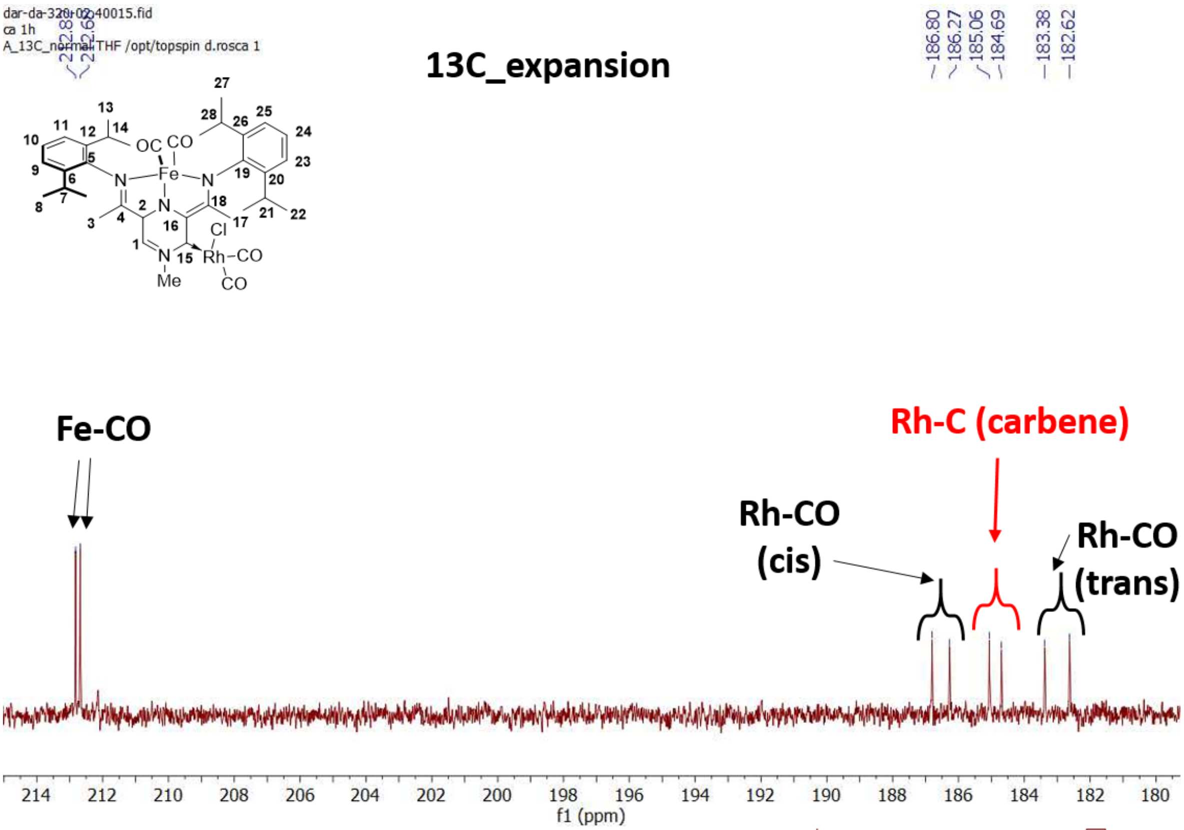
dar-da-320-02-40015.fid
ca 1h
A_13C_normal THF /opt/topspin d.rosca 1
13C_expansion
186.80
186.27
185.06
184.69
183.38
182.62
Fe-CO
Rh-C (carbene)
Rh-CO
(cis)
Rh-CO
(trans)
f1 (ppm)

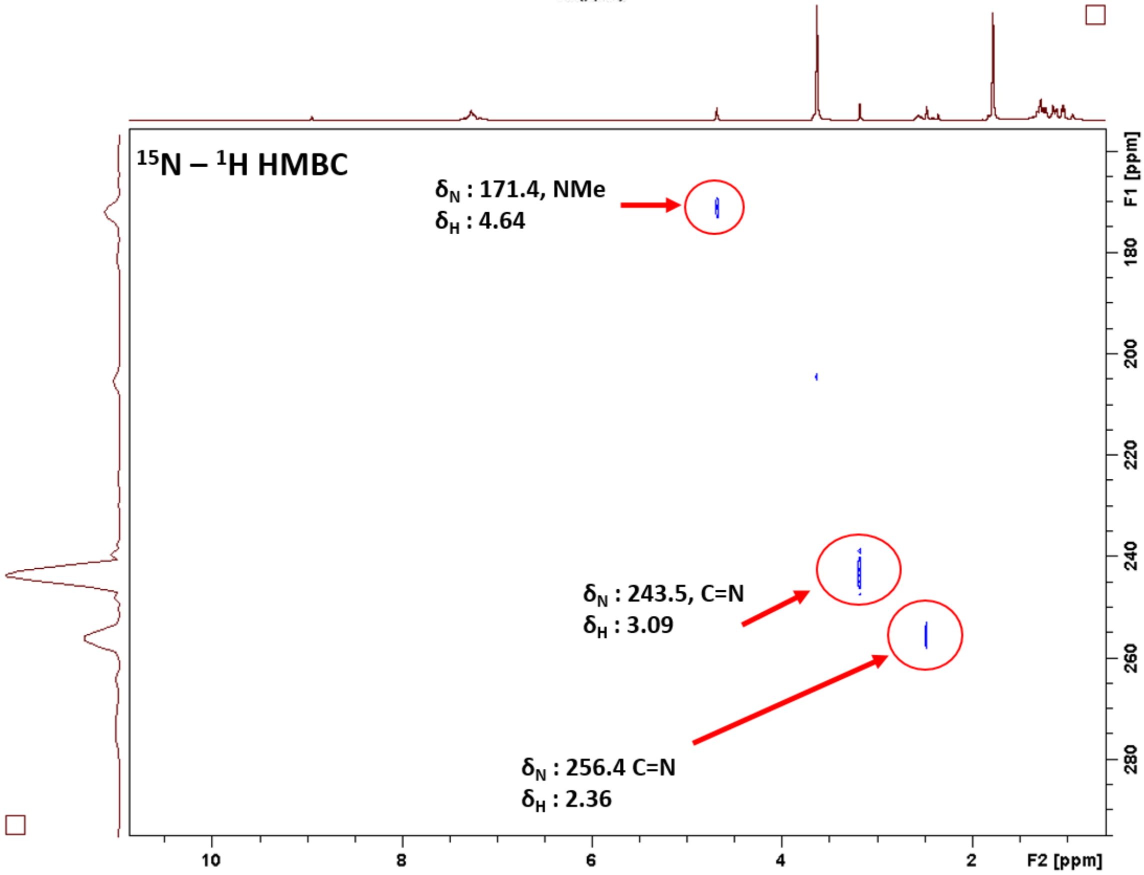
$^{15}N - ^{1}H$ HMBC
δ_N : 171.4, NMe
δ_H : 4.64
δ_N : 243.5, C=N
δ_H : 3.09
δ_N : 256.4 C=N
δ_H : 2.36
F1 [ppm]
F2 [ppm]

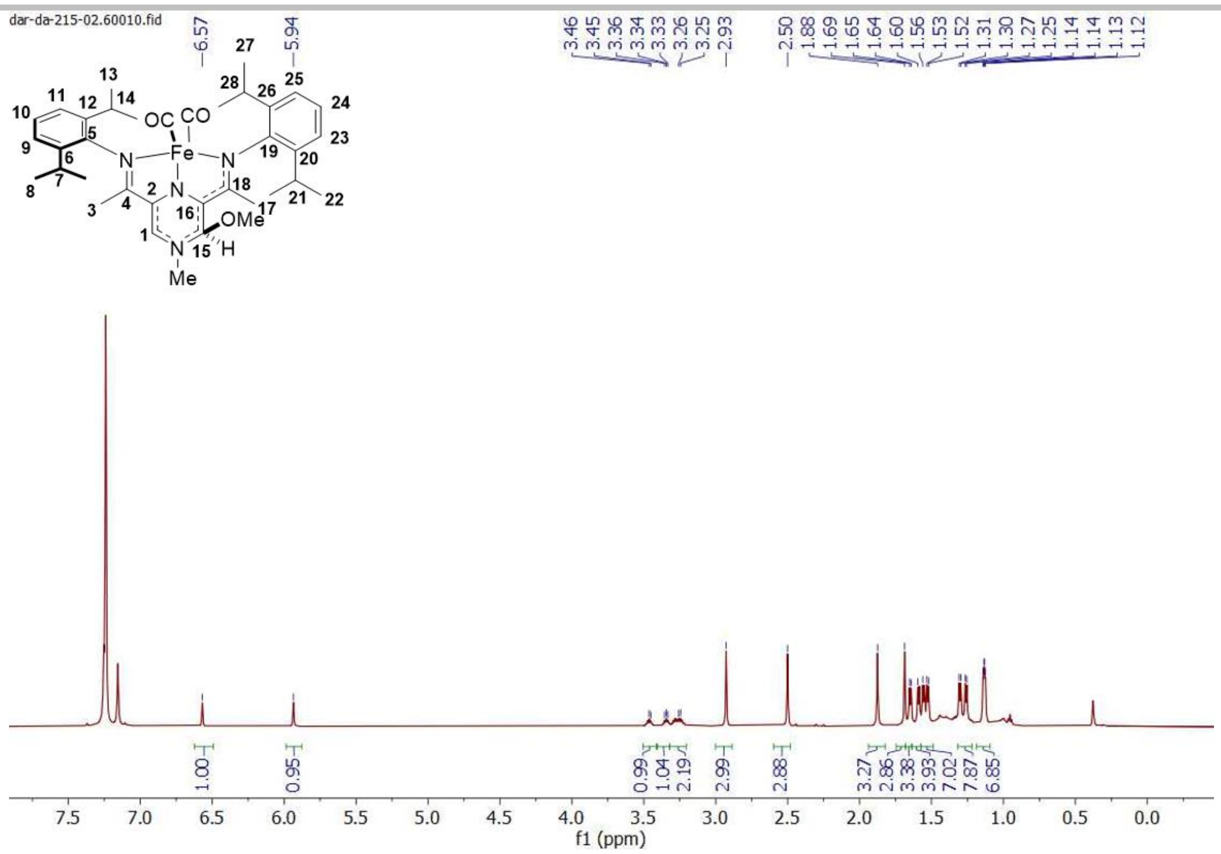
dar-da-215-02.60010.fid
6.57
5.94
3.46
3.45
3.36
3.34
3.33
3.26
3.25
2.93
2.50
1.88
1.69
1.65
1.64
1.60
1.56
1.53
1.52
1.31
1.30
1.27
1.25
1.14
1.14
1.13
1.12
OC
CO
Fe
N
OMe
H
Me
1.00
0.95
0.99
1.04
2.19
2.99
2.88
3.27
2.86
3.38
3.93
7.02
7.87
6.85
f1 (ppm)

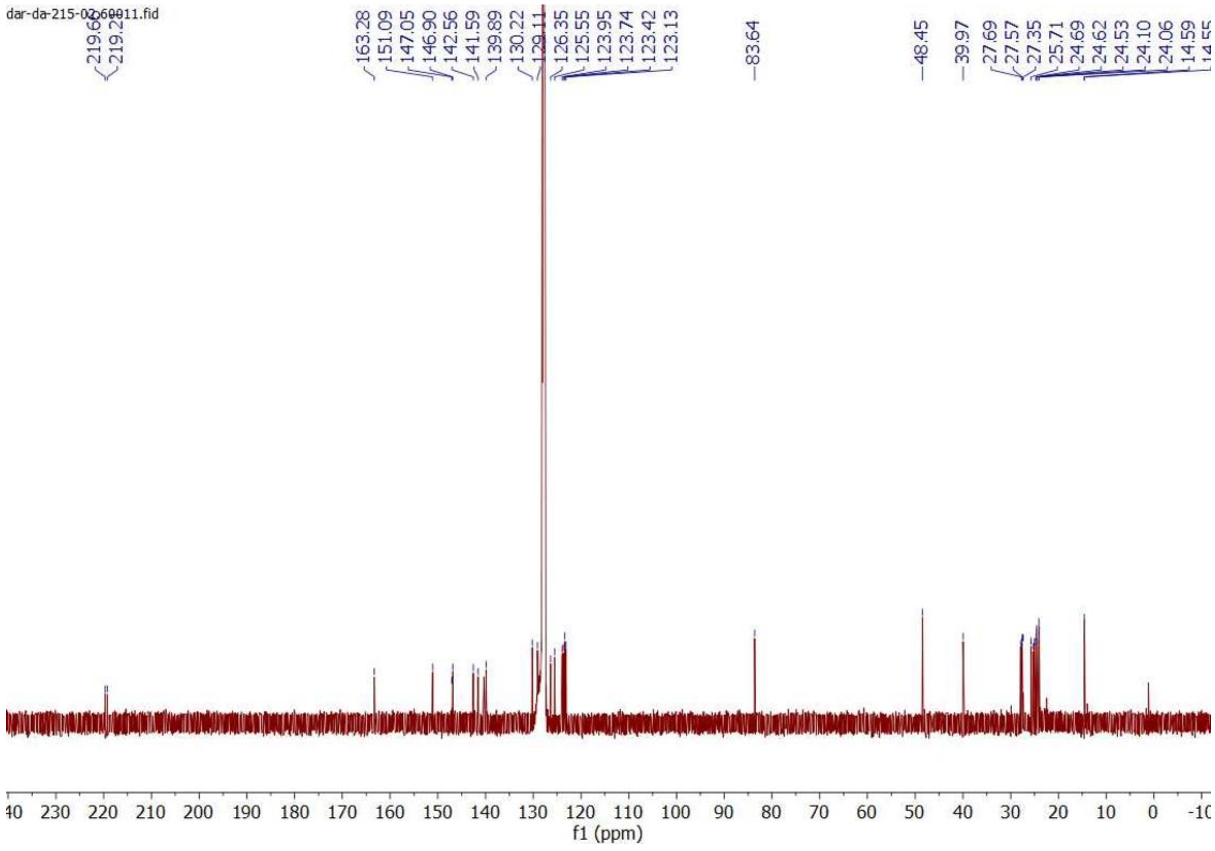
dar-da-215-02.60011.fid
219.66
219.2
163.28
151.09
147.05
146.90
142.56
141.59
139.89
130.22
129.11
126.35
125.55
123.95
123.74
123.42
123.13
83.64
48.45
39.97
27.69
27.57
27.35
25.71
24.69
24.62
24.53
24.10
24.06
14.59
f1 (ppm)

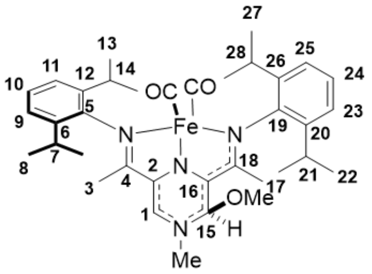
OC
CO
Fe
N
OMe
H
Me
1
2
3
4
5
6
7
8
9
10
11
12
13
14
15
16
17
18
19
20
21
22
23
24
25
26
27
28

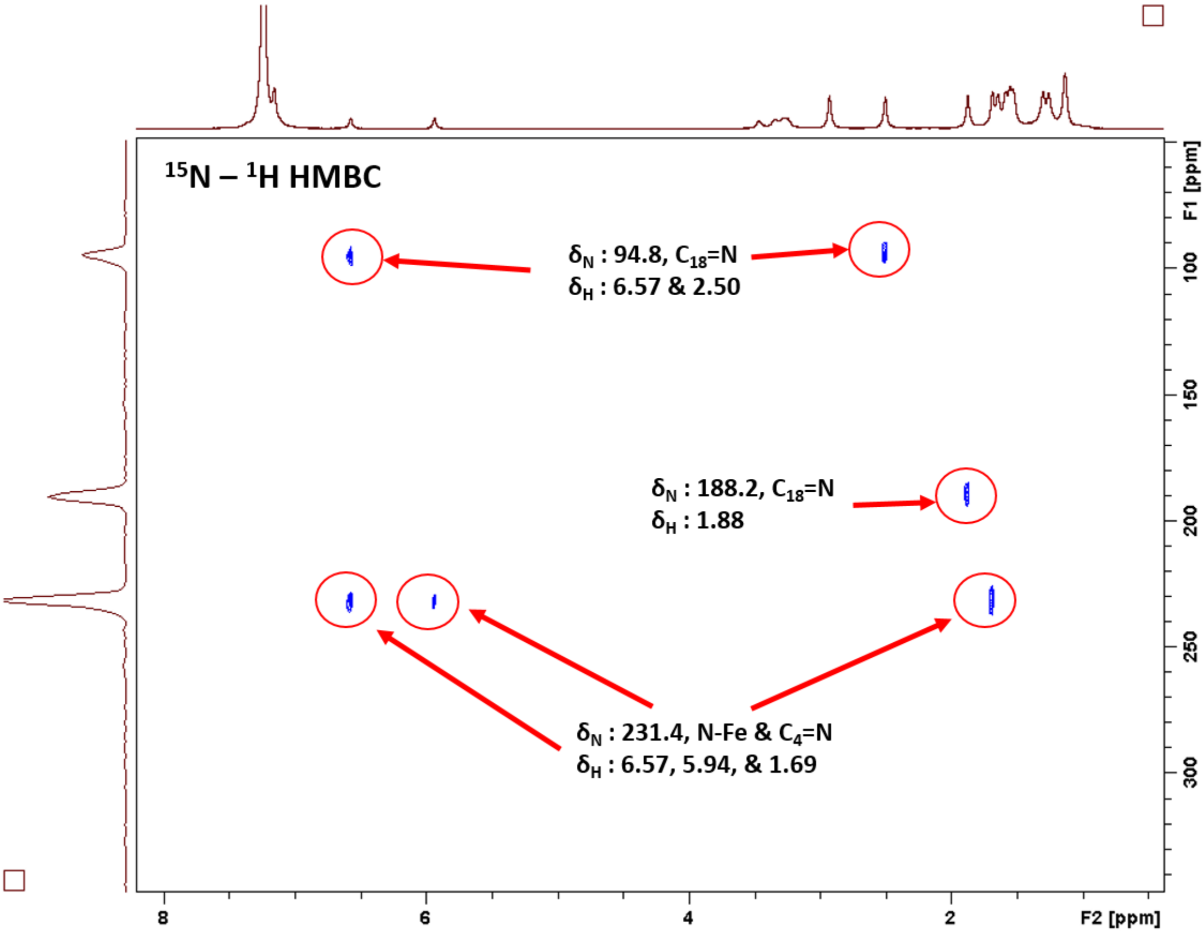
15N – 1H HMBC
δN : 94.8, C18=N
δH : 6.57 & 2.50
δN : 188.2, C18=N
δH : 1.88
δN : 231.4, N-Fe & C4=N
δH : 6.57, 5.94, & 1.69
F1 [ppm]
100
150
200
250
300
8
6
4
2
F2 [ppm]

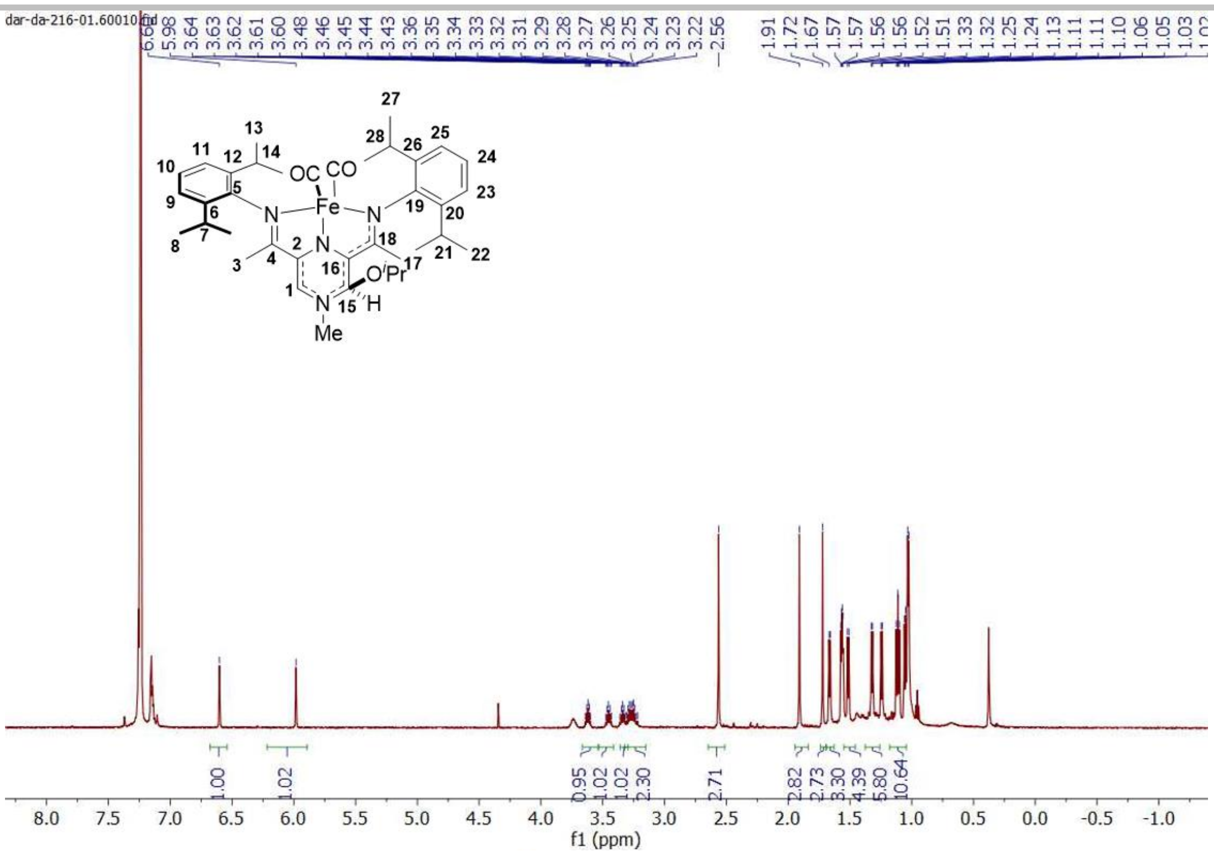
dar-da-216-01.60010.fid
f1 (ppm)
OC CO
Fe
N
OⁱPr
H
Me

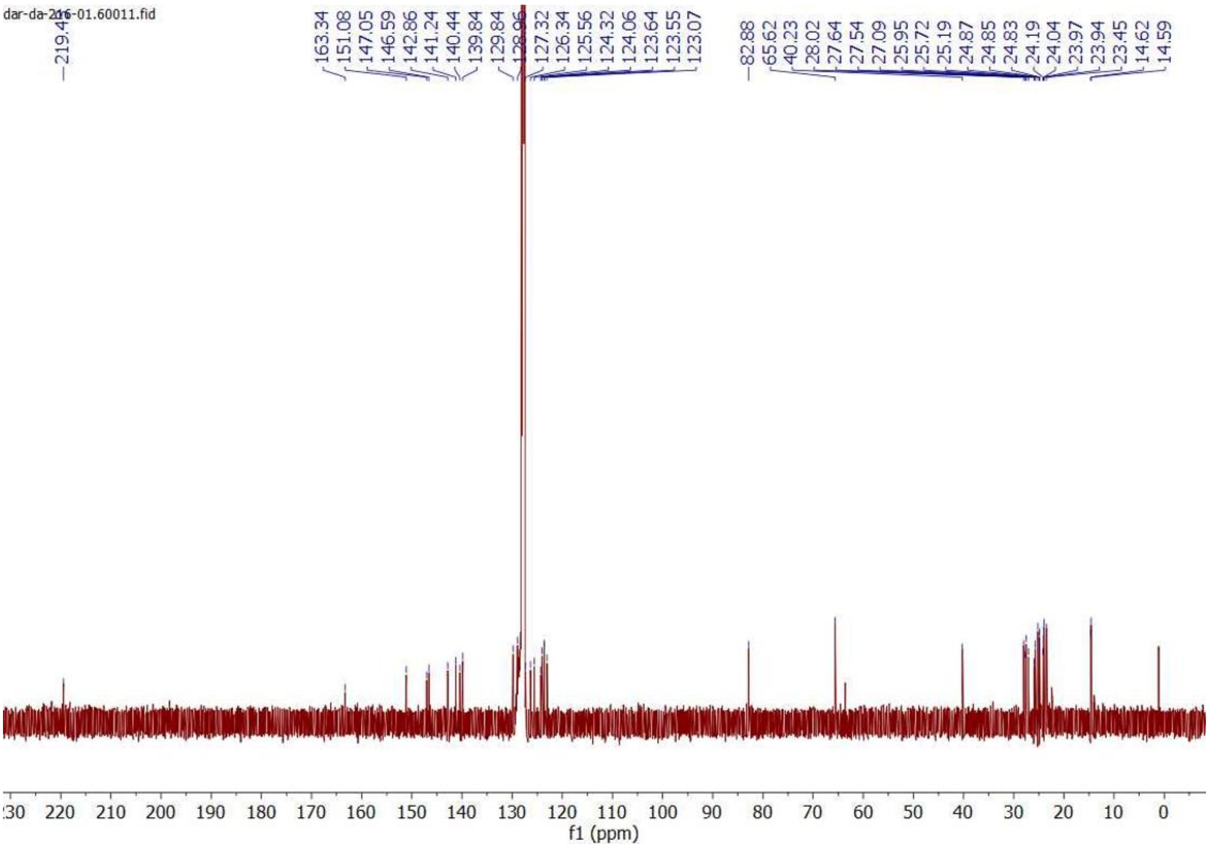
dar-da-216-01.60011.fid
f1 (ppm)

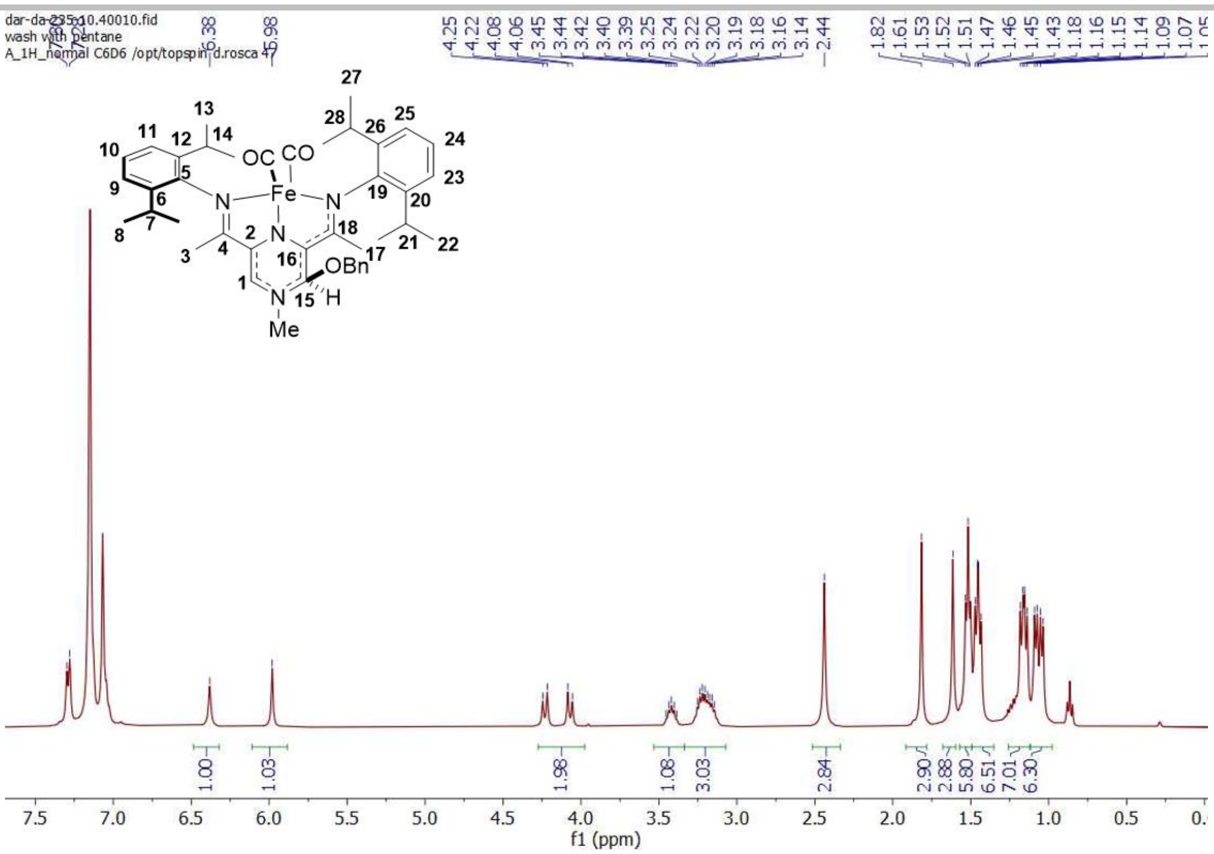
dar-da-235-10.40010.fid
wash with pentane
A_1H_normal C6D6 /opt/topspin d.rosca 47
f1 (ppm)

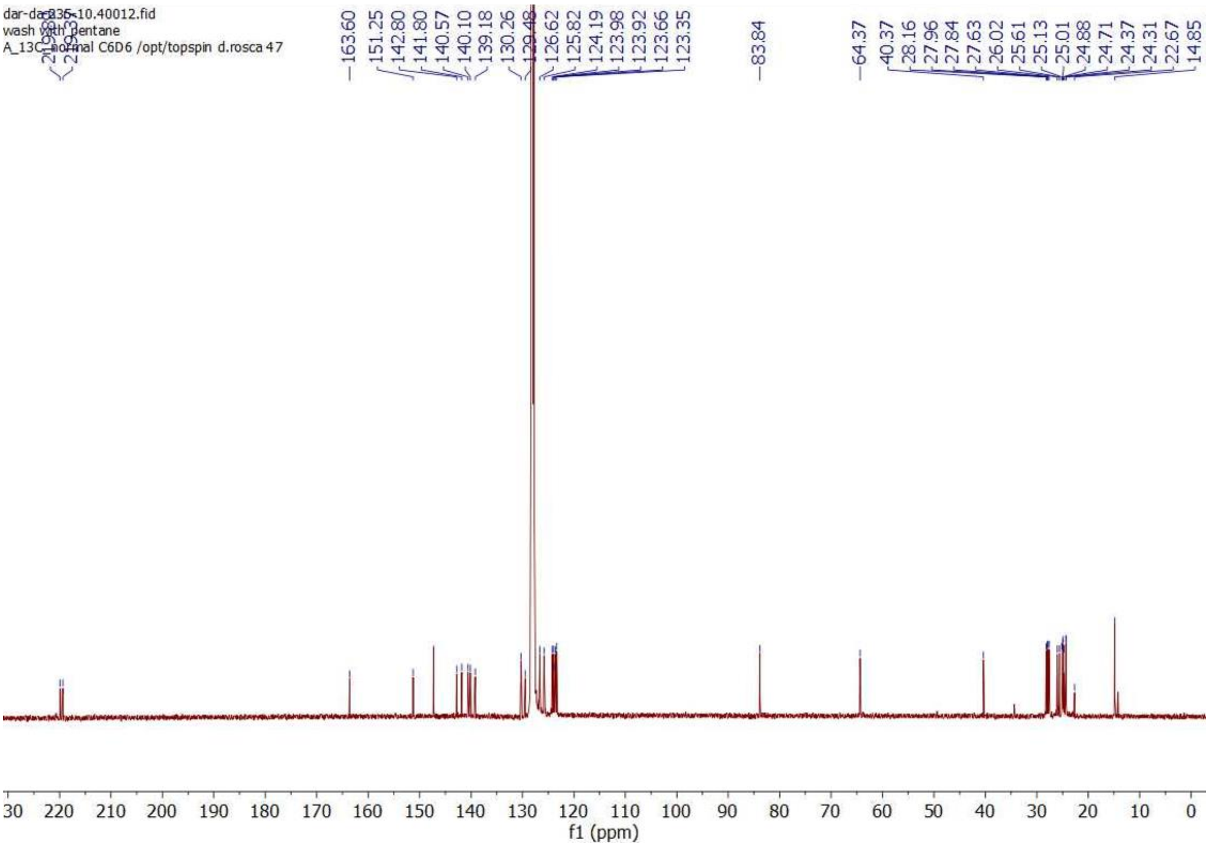
dar-da-235-10.40012.fid
wash with pentane
A_13C_normal C6D6 /opt/topspin d.rosca 47
f1 (ppm)

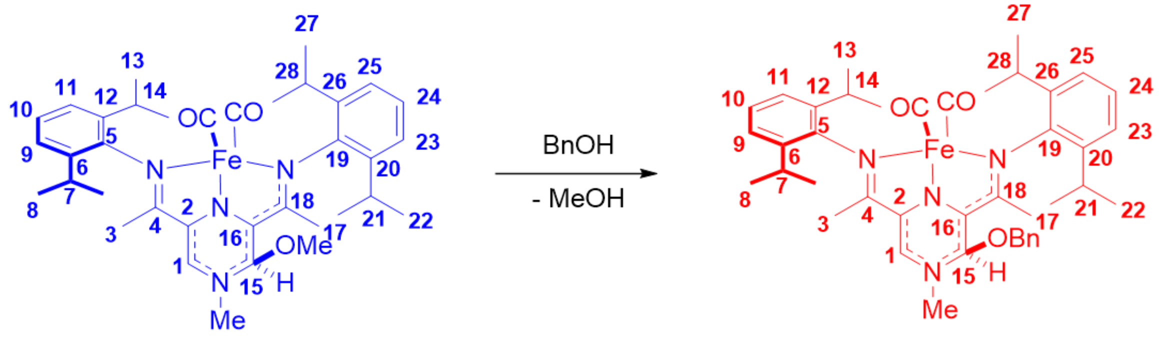

Selected assignments below are colour coded

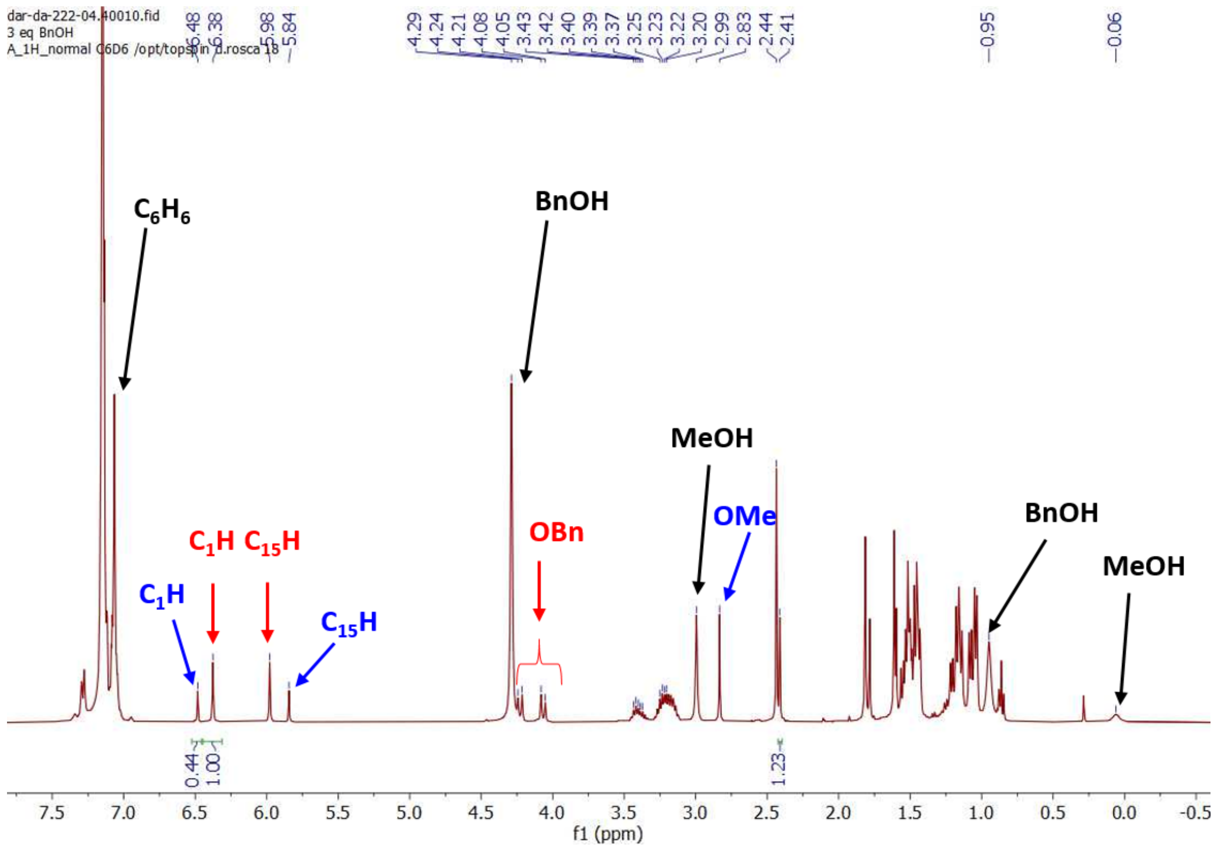

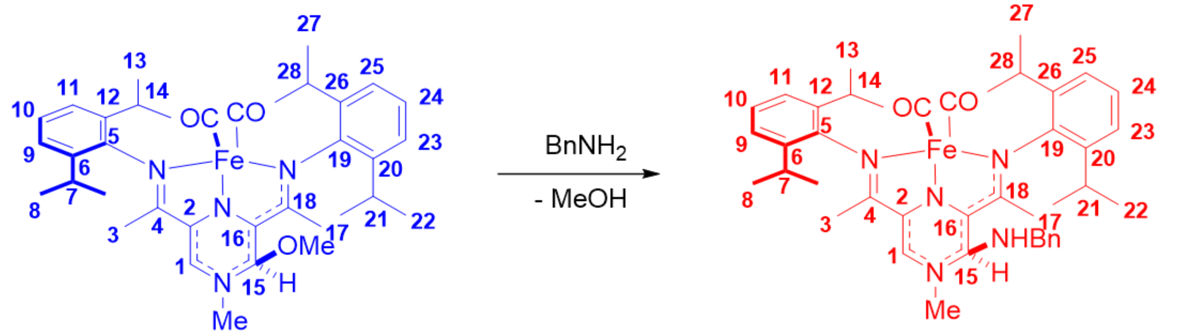

Selected assignments below are colour coded

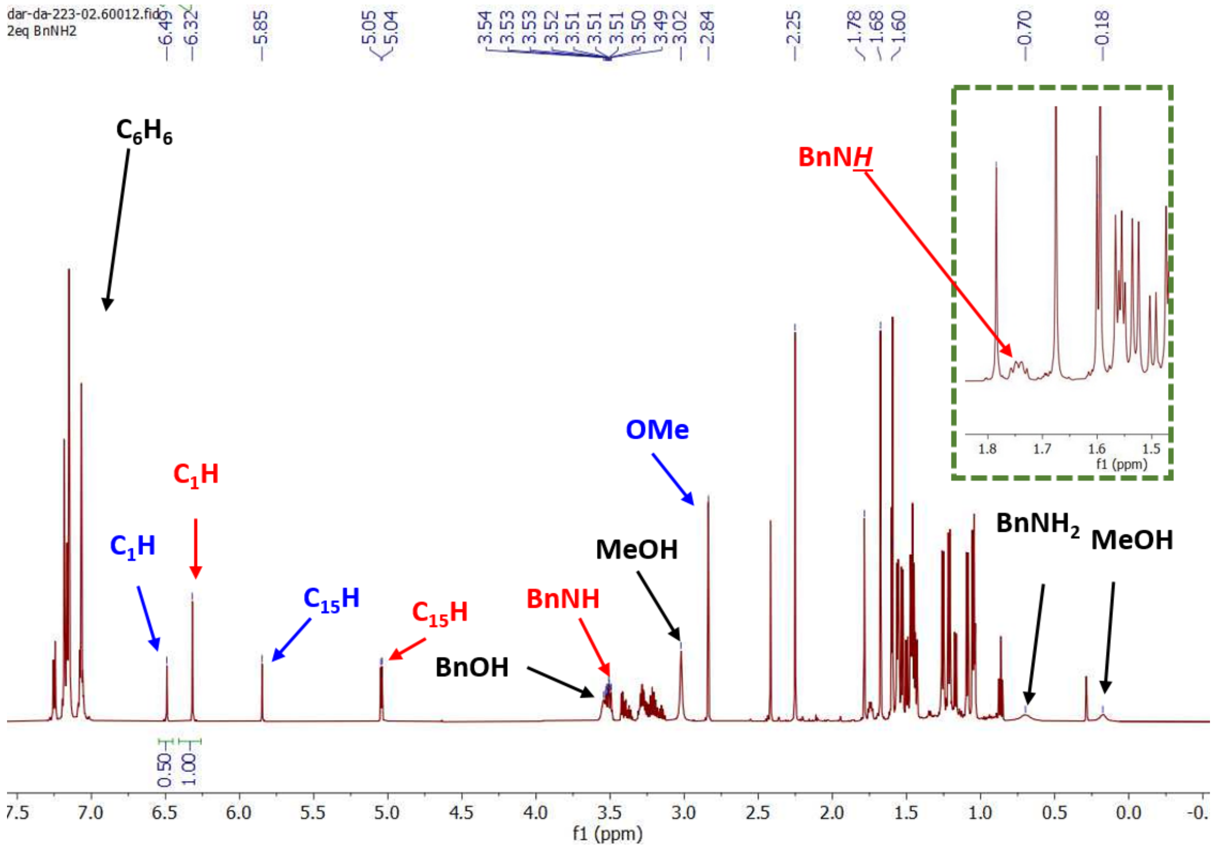

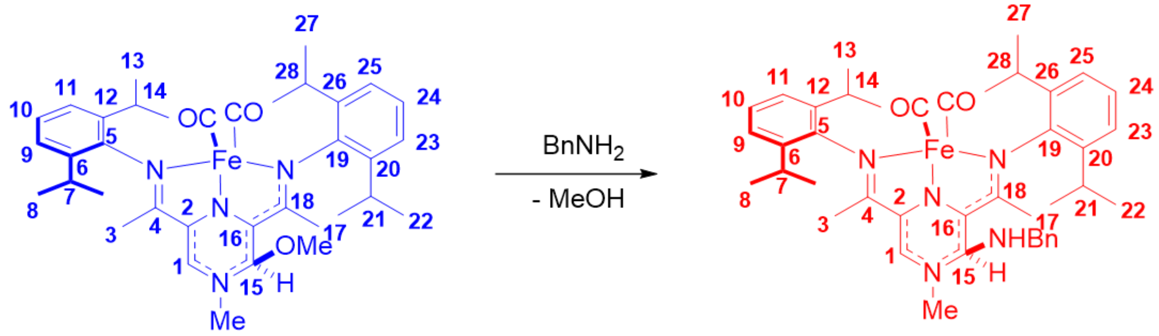

Only assignments for product are given

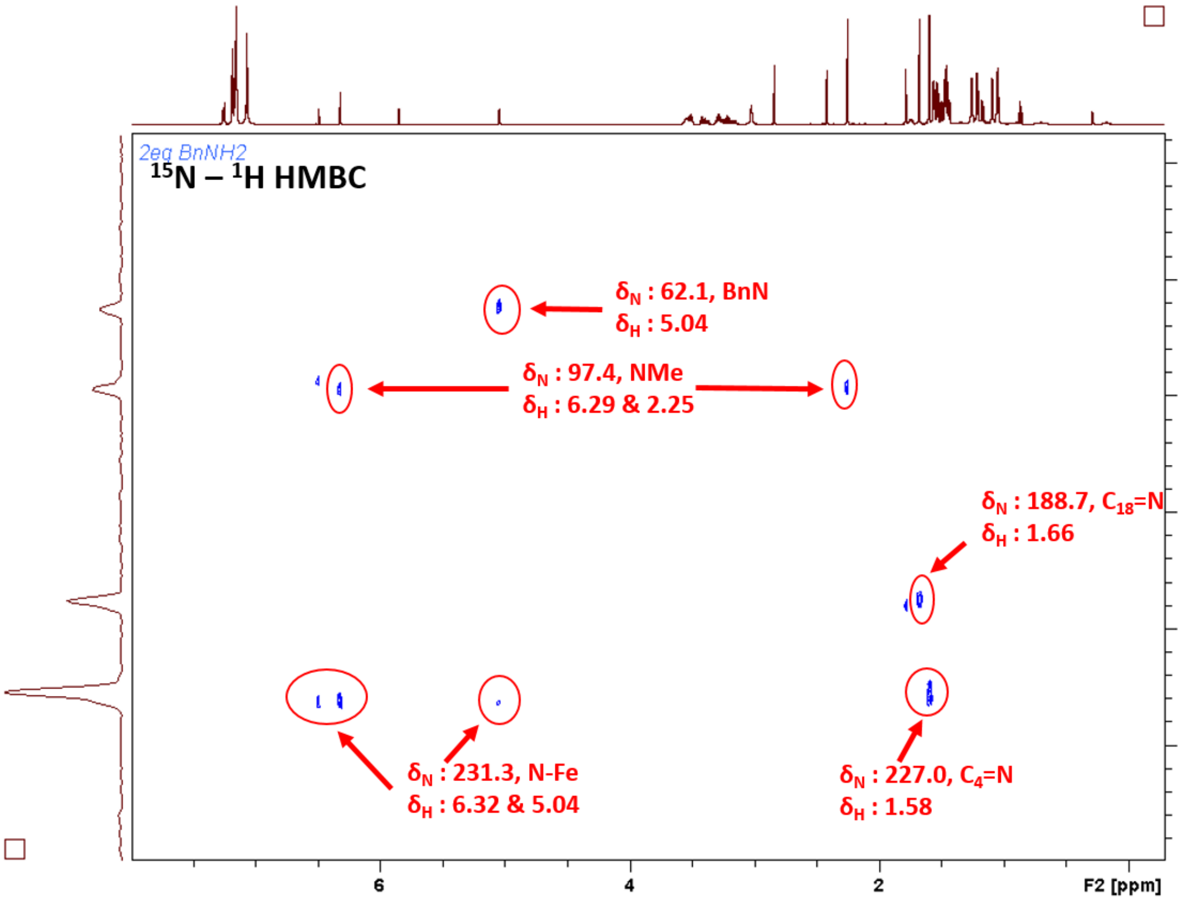

IR Spectra

Table S5. Solid state (ATR) and solution IR Stretching frequencies (cm^{-1}) for selected compounds

Number	Compound	**ν_{CO} (cm^{-1})** *(sym, asym)*(ATR)	**ν_{CO} (cm^{-1})** *(sym, asym)[soln]**
**1 · [I]**	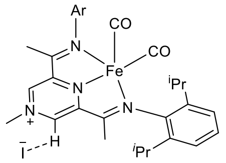	1999, 1938	2010, 1952
**6**	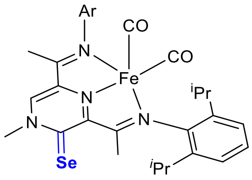	1992, 1938	
**7**	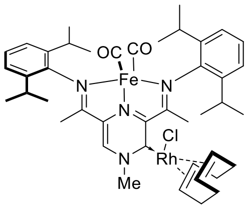	1978, 1918	
**8**	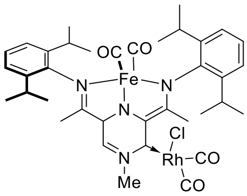	2066, 1988, 1926	2074, 1995, 1931
**9**	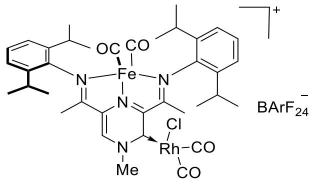	2084, 2045, 1946	2081, 2007, 1948
**10**	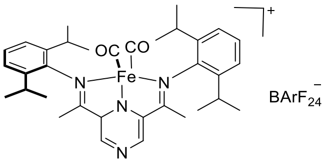	2003, 1946	
**11**	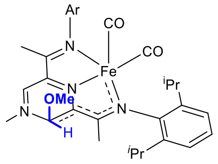	1949, 1882	1974, 1906 (pentane)
**12**	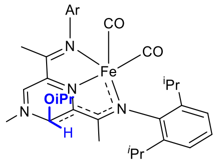	1947, 1880	

* Recorded in CH_2Cl_2 solutions (KBr plates, liquid cell), unless otherwise stated

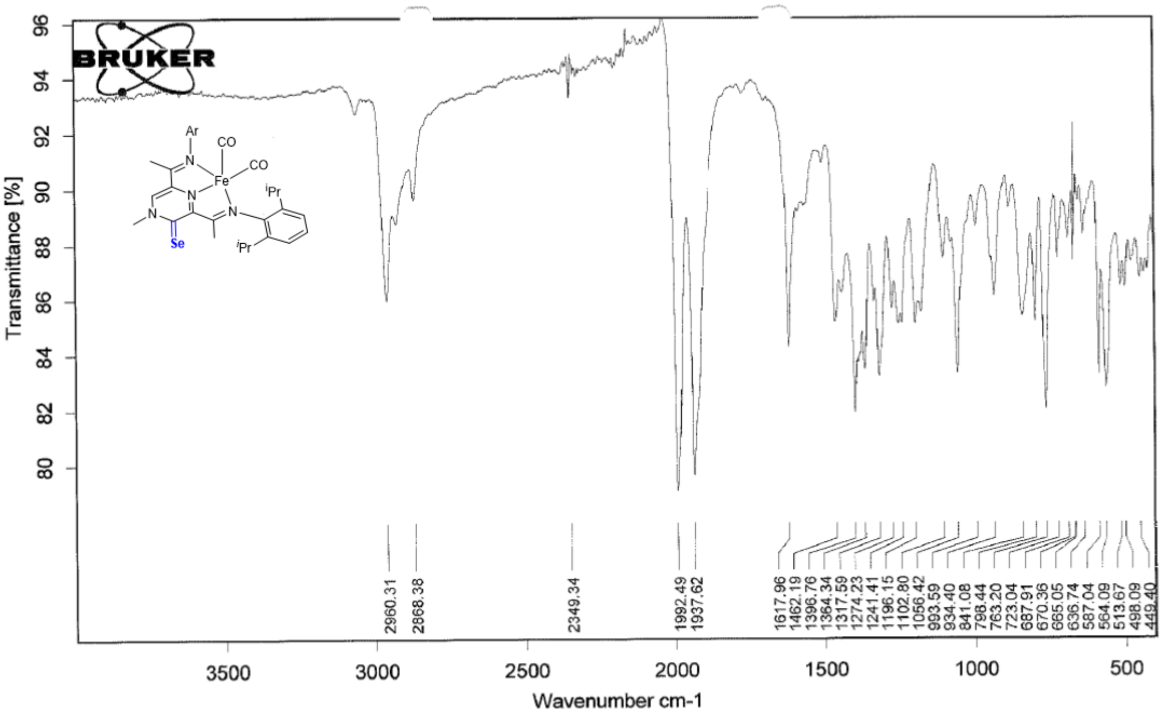
BRUKER
Transmittance [%]
Wavenumber cm-1
Ar
CO
CO
Fe
N
Se
iPr
2960.31
2868.38
2349.34
1992.49
1937.62
3500
3000
2500
2000
1500
1000
500

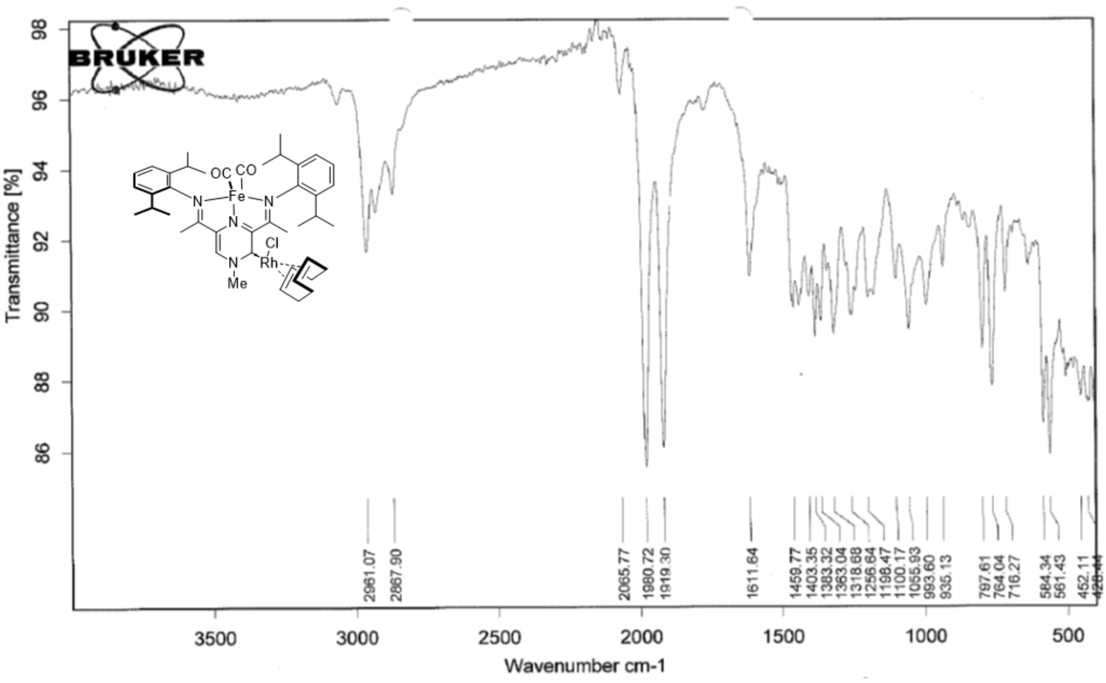
BRUKER
Transmittance [%]
Wavenumber cm-1
OC
CO
Fe
N
Cl
Rh
Me
2961.07
2867.90
2065.77
1980.72
1919.30
1611.64
3500
3000
2500
2000
1500
1000
500

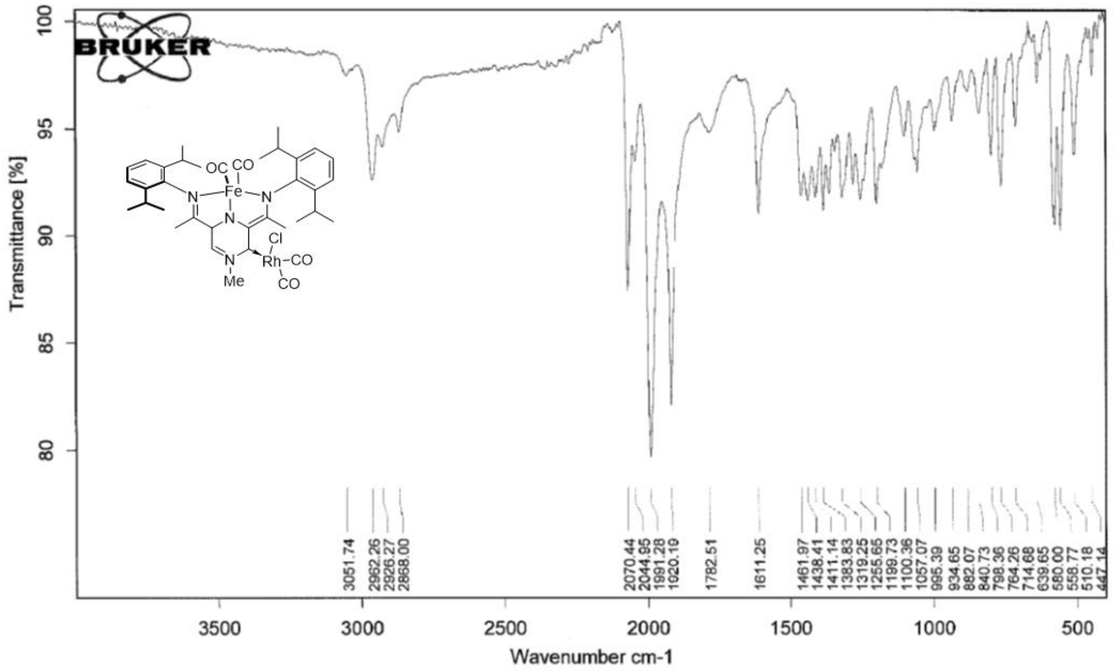

Solid state

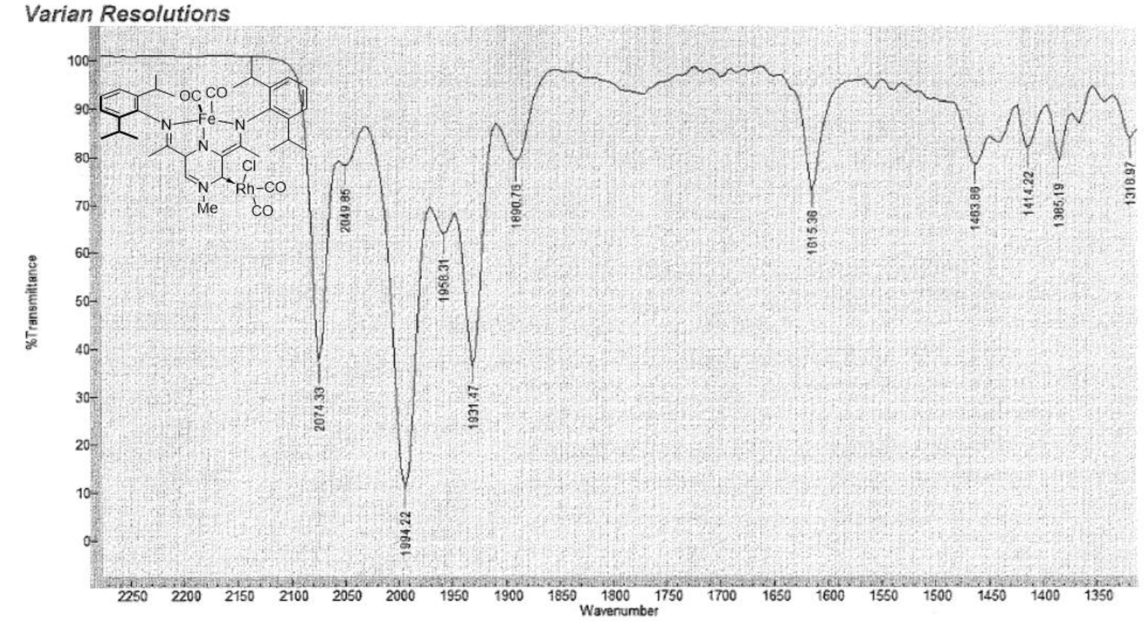

CH_2Cl_2 solution / KBr liquid cell

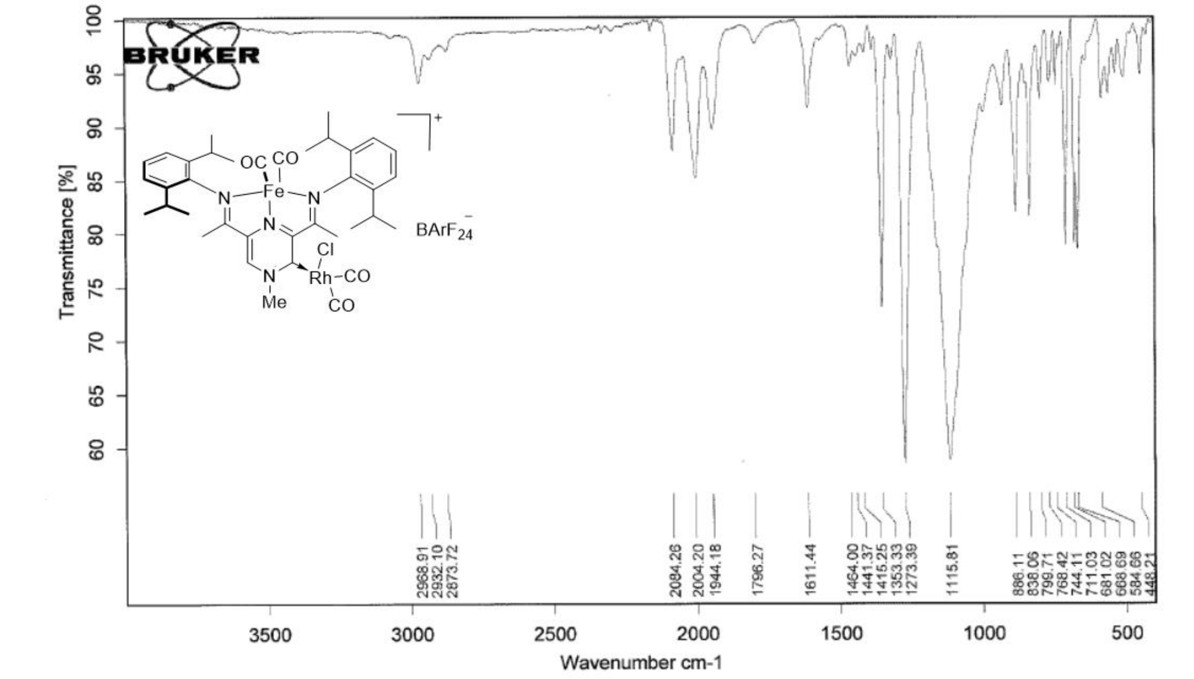

Solid state

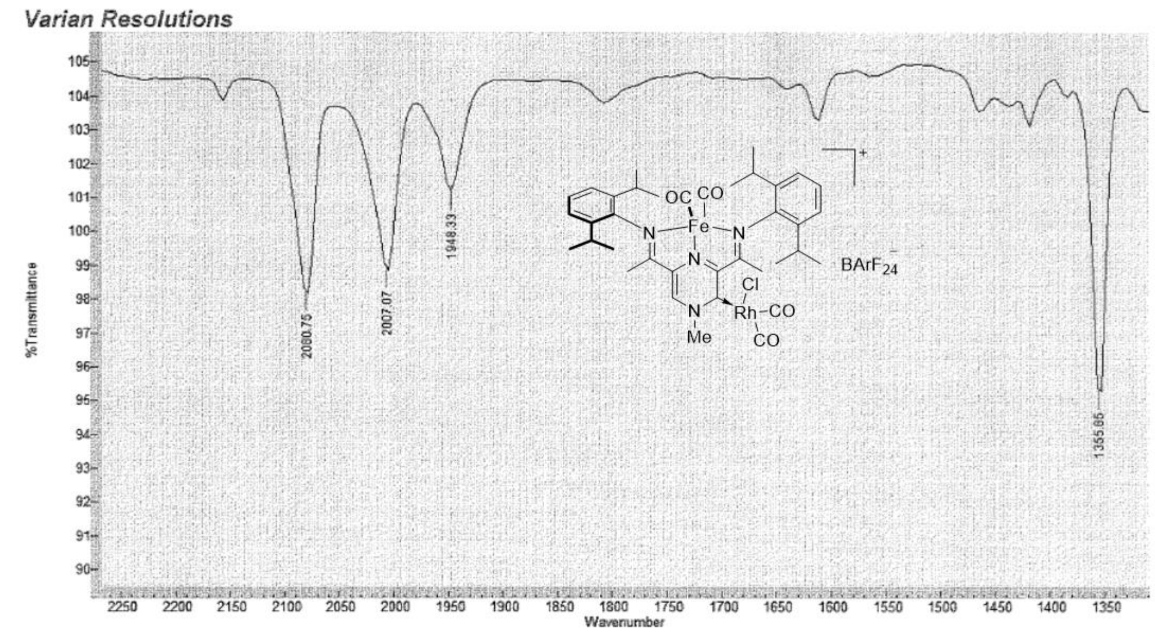

CH_2Cl_2 solution / KBr liquid cell

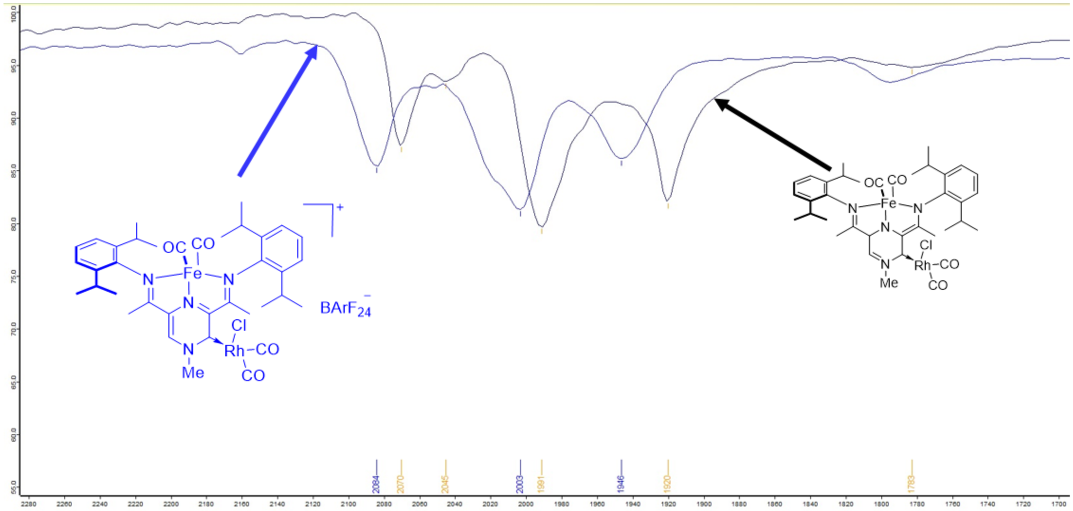

Comparison of solid state (ATR) spectra of 8 (blue) and 9 (black)

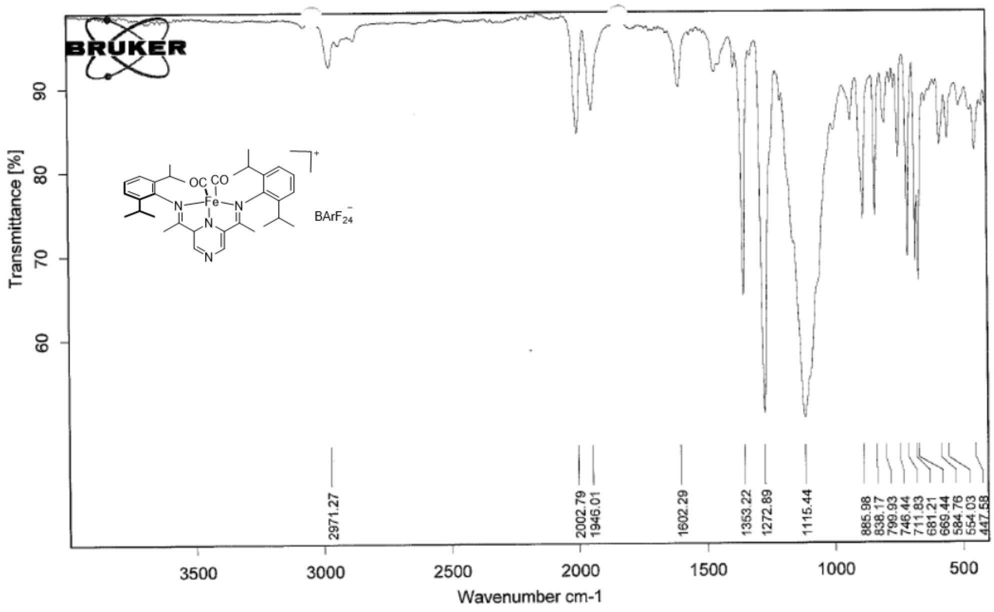

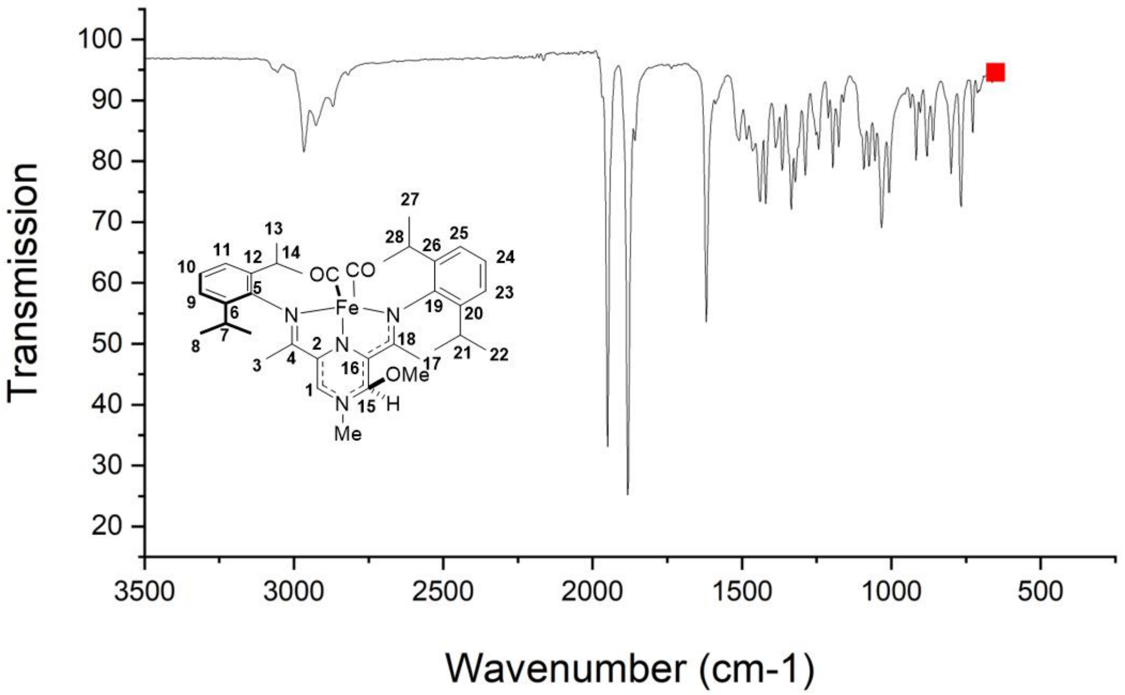
Transmission
Wavenumber (cm-1)
OC
CO
Fe
N
OMe
H
Me
1
2
3
4
5
6
7
8
9
10
11
12
13
14
15
16
17
18
19
20
21
22
23
24
25
26
27
28

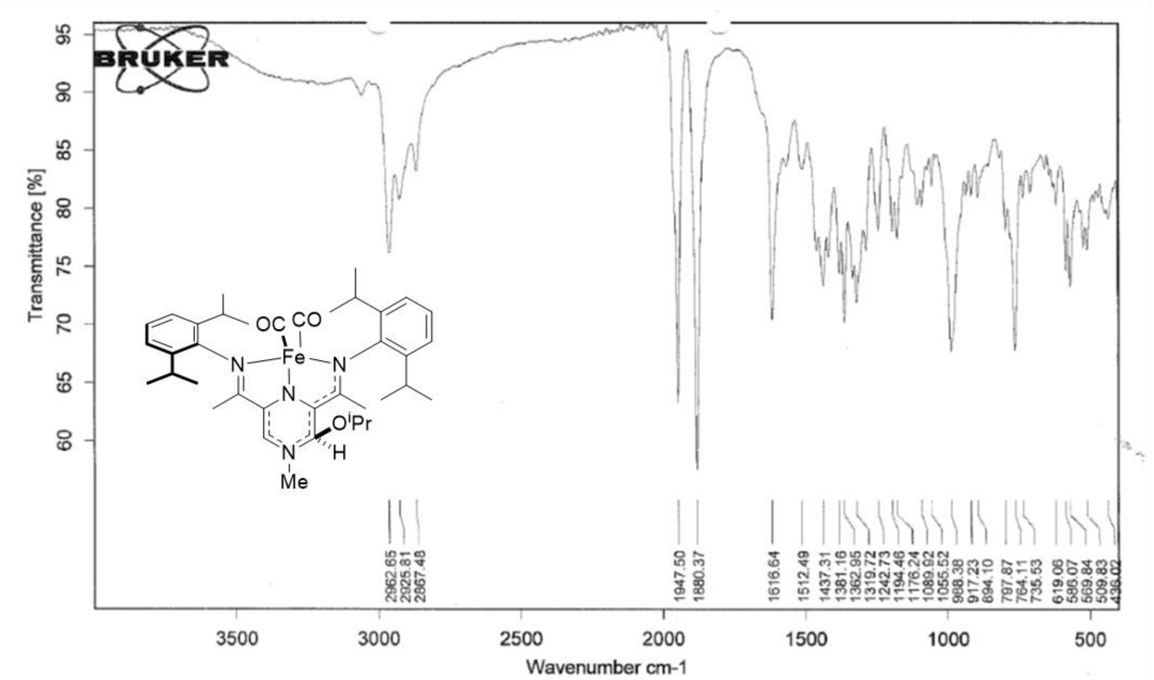
BRUKER
Transmittance [%]
Wavenumber cm-1
OC
CO
Fe
N
O^{i}Pr
H
Me
2962.65
2925.81
2867.48
1947.50
1880.37
1616.64
1512.49
1437.31
1381.16
1362.95
1319.72
1242.73
1194.46
1176.24
1089.92
1055.52
988.38
917.23
894.10
797.87
764.11
735.53
619.06
586.07
569.84
509.83
436.02

Cyclic Voltammetry

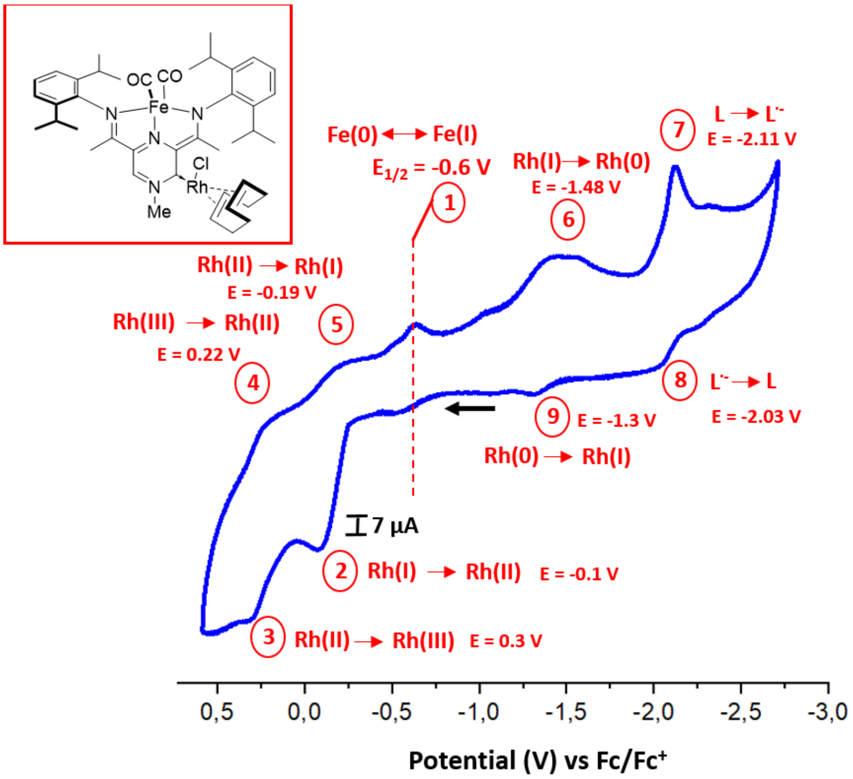

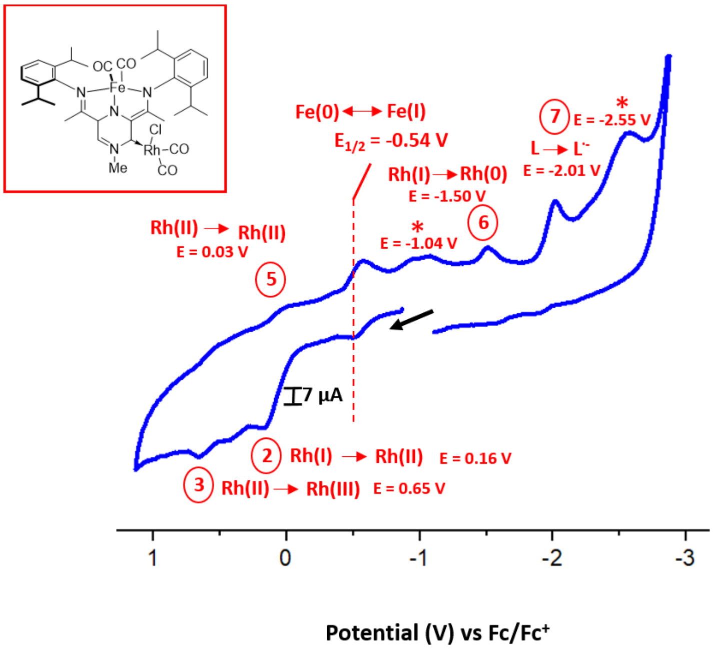

EPR Spectroscopy

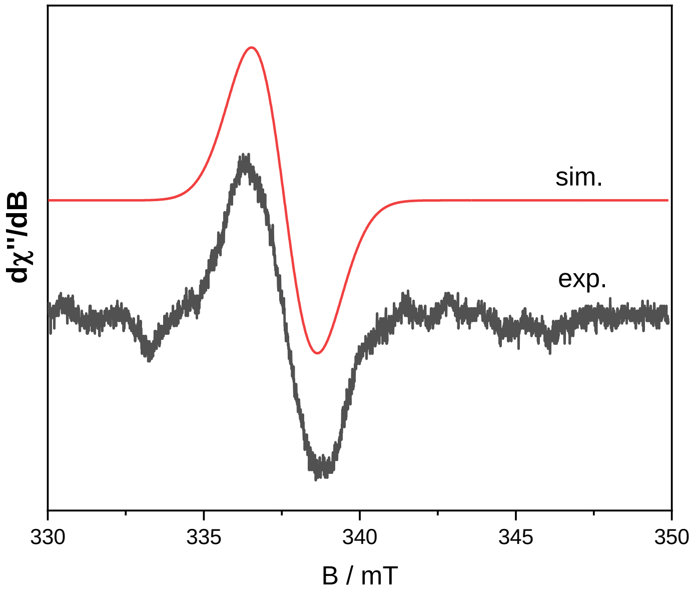

Figure S7. Experimental (black) and simulated (red) X-band CW-EPR spectrum of **9** recorded at RT in THF solution. Fitted parameter: g = 1.999

Kinetic data

ReactIR data

Experimental procedure: A reaction flask fitted with an *in situ* IR probe was evacuated and backfilled with Ar (three cycles), followed by the addition of a solution of **7** (3.9 mg, 4.58 µmol) in THF (3.7 mL). The background was collected, and the solution was allowed to equilibrate at 20°C for 10 minutes. Constant temperature was maintained by employing a cryostat. 4,4'-difluorobenzophenone (50 mg, 229 µmol) in THF (0.1 mL) was added, followed by a solution of Ph_2SiH_2 (48.5 µL, 229.1 µmol) in THF (0.1 mL). When **7**$^+$ was generated, [Fc][BArF$_{24}$] (4.81 mg, 4.58 µmol) in THF (50 µL) was added. For the reduction of **7**$^+$ back to **7**, a solution of cobaltocene (0.87 mg, 4.58 mmol) in THF (50 µL) was added. Measurements were taken every 15 seconds. The concentration of the silane was determined by following the sharp wagging vibration mode at 846 cm^{-1}. 4,4'-difluorobenzophenone, Ph_2SiH_2, [Fc][BArF$_{24}$] and cobaltocene were handled as stock solutions in THF.

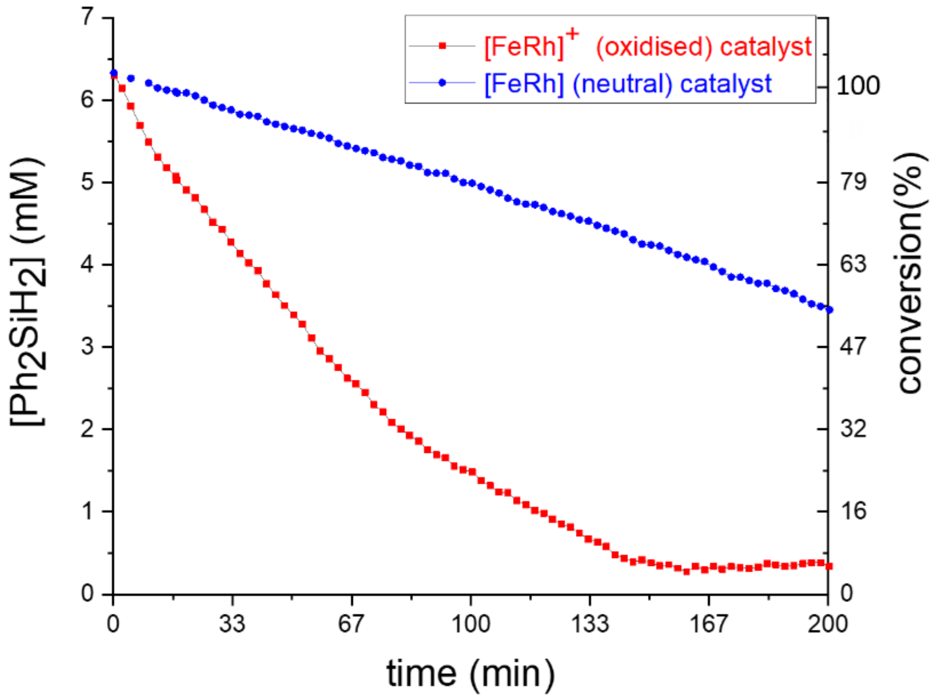

Figure S8. Plot of Ph_2SiH_2 consumption over time for the hydrosilylation of 4,4'-difluorobenzophenone employing either the neutral [FeRh] complex **7** (blue trace), or its oxidised analogue **7**$^+$ (red trace). For clarity, only the every 10th data point is displayed.

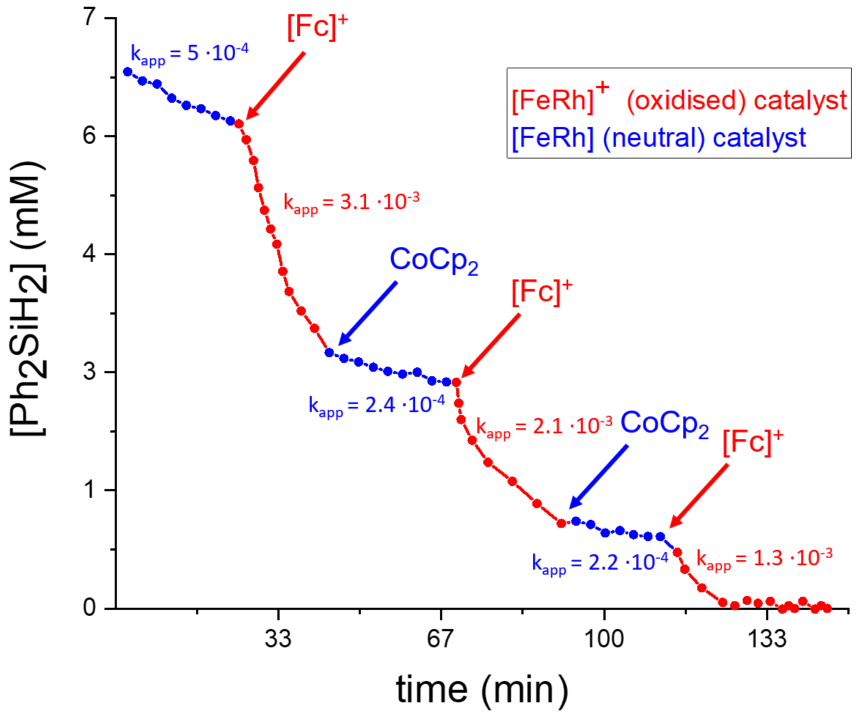

Figure S9. Plot of Ph_2SiH_2 consumption over time for the hydrosilylation of 4,4'-difluorobenzophenone during the *in situ* oxidation and reduction of complex **7/7**$^+$ illustrating temporal control. For clarity, only the every 10th data point is displayed. Apparent relative reaction rates (given in mmol/L·s) were determined by employing the initial rates method (by determing the slope of the liniar protion of the various kinetic regimes)

NMR data

Experimental procedure: In a glovebox, **7** (3.92 mg, 4.58 µmol) together with [Fc][BArF$_{24}$] (4.81 mg, 4.58 µmol) were weighed in a vial, to which THF-d$_8$ (0.6 mL) was added. The reaction mixture was briefly shaken, before the addition of 4,4'-difluorobenzophenone (50 mg, 229.1 µmol) and the internal standard hexafluorobenzene (10 µL, 86.5 µmol). The resulting solution was transferred into a J-Young NMR tube, and Ph_2SiH_2 (49 µL, 231 µmol) was added. The NMR tube was then inserted in an NMR spectrometer and ^{19}F{^{1}H} NMR spectra were collected every 4 minutes. The concentration of the 4,4-difluorobenzophenone (δ_{19F} -107.8) and silylether product (δ_{19F} -116.3) was determined by integrating the ^{19}F resonances against the internal standard. Constant temperature (20°C) in the spectrometer was maintained by employing a BCU unit. NMR data for product: **^{1}H NMR** (400 MHz, THF-*d*$_8$) 7.69 – 7.55 (m, 6H), 7.50 – 7.27 (m, 8H), 7.10 – 6.96 (m, 4H), 6.00 (s, 1H) 5.44 (s, 1H) **^{19}F{^{1}H} NMR** (386 MHz, THF-*d*$_8$) -116.3

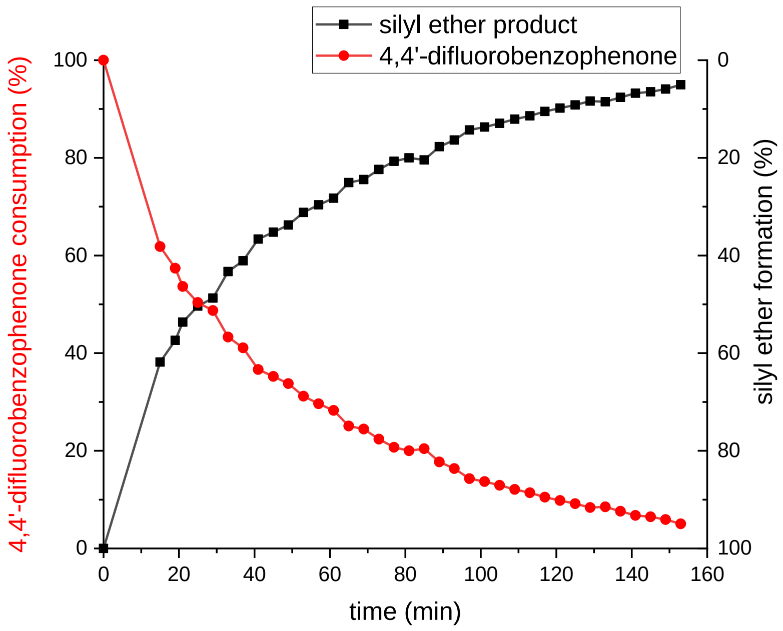

Figure S10. Plot of 4,4'-fluorobenzophenone consumption (red trace) and the silylether formation (black trace) as determined by 19F{1H} NMR spectroscopy. **7⁺** was employed as catalyst.

Computational Details

All density functional theory (DFT) calculations were performed using the ORCA quantum chemical program package (Version 4.1.2).[22] Geometry optimisations of the complexes **7, 8, 10, 11** and the Np-substituted diaminocarbene[3]ferrocenophane[23] (Np = Neopentyl) were performed using the corresponding crystal structures, without any truncation of their structures, as starting geometries. Computational models for the oxidised [MeP^{Pz}DIFe(CO)$_2$(RhCODCl)]$^+$ and **9** were built starting from the crystal structures of **7** and **8** respectively, without the inclusion of the BArF$_{24}$ counteranion in the calculations. The computational model for **2** was built from the crystal structure of **[1]·I.** Geometry optimisations of the computational models were undertaken by employing the hybrid-GGA (GGA = generalised gradient approximation) density functional, B3LYP,[24,25] in conjunction with Ahlrichs triple-ζ def2-TZVP(-f) basis set.[26] To speed up the overall calculations, the RIJCOSX[27] approximation was applied for the expensive integral calculations. Noncovalent interactions were accounted for by using atom-pairwise dispersion corrections with Becke-Johnson (D3BJ) damping.[28] For complexes **2**, **7**, **8** and **11,** spin unrestricted Broken Symmetry (BS) calculations were performed, to check for ligand non-innocence. Subsequently, these structures, as well as the Np-substituted diaminocarbene[3]ferrocenophane were calculated as restricted singlets. The oxidised species [MeP^{Pz}DIFe(CO)$_2$(RhCODCl)]$^+$ **9** and **10** were calculated as unrestricted doublets. Subsequent analytical frequency calculations were undertaken for the optimized geometries to confirm they correspond to stationary points featuring no imaginary frequencies below 50 cm^{-1}. The zero-point vibrational energies, thermal corrections and entropy terms were obtained from the frequency calculations. The vibrational frequencies were corrected using the tabulated scaling factor of 0.965 according to the computational chemistry comparison benchmark data base (cccbdb).[29] Molecular orbitals were visualised with Avogadro (Version 1.2.0) and plotted with an isosurface value of 0.05. NBO calculations were performed with the NBO 7.0 program package.[30]

---

[22] (a) F. Neese *WIREs Comput. Mol. Sci.* **2012**, *2*, 73-78. b) F. Neese *Wiley Interdiscip. Rev.: Comput. Mol. Sci.* **2017**, *8*, e1327. Doi: 10.1002/wcms.1327
[23] U. Siemeling, C. Färber, M. Leibold, C. Bruhn, P. Mücke, R. F. Winter, B. Sarkar, M. von Hoffgarten, G. Frenking *Eur. J. Inorg. Chem.* **2009**, 4607 – 4612.
[24] A. D. Becke *Phys. Rev. A.* **1988**, *38*, 785.
[25] C. Lee, W. Yang, R. G. Parr *Phys. Rev. B.* **1988,** *38*, 785.
[26] A. Schäfler, C. Huber, R. Ahlrichs *J. Chem. Phys.* **1994**, *100*, 5829.
[27] F. Neese, F. Wennmohs, A. Hansen, U. Becker *Chem. Phys.* **2009**, *356,* 98.
[28] S. Grimme, S- Ehrlich, L. J. Goerigk *Comput. Chem.* **2011**, *32*, 1456.
[29] For metal carbonyl complexes, see also M. K. Assefa, J. L. Devera, A. D. Brathwaite, J. D. Mosley, M. A. Duncan *Chem. Phys. Lett.* **2015**, *640*, 175.
[30] NBO 7.0 E. D. Glendening, J. K. Badenhoop, A. E. Reed, J. E. Carpenter, J. A. Bohmann, C. M. Morales, P. Karafiloglou, C. R. Landis, F. Weinhold Theoretical Chemistry Institute, University of Wisconsin, Madison, WI (2018).

Broken-Symmetry Calculations

The broken symmetry (BS) formalism[31] was employed in unrestricted calculations to check for antiferromagnetic coupling of two spins. BS calculations were performed for complexes **2**, **7**, **8** and **11** using the B3LYP functional and the same basis set (def2-TZVP(-f)) as mentioned earlier. Because several BS solutions of the spin-unrestricted Kohn – Sham equations may be obtained, the general notation BS(*m,n*) was used, where *m (n)* denotes the number of spin-up (spin-down) electrons at the interacting fragments. The spin multiplicity for the broken symmetry calculations were chosen according to the high spin state - triplet for BS(1,1) and quintet for BS(2,2).

For the aforementioned complexes, BS(1,1) and BS(2,2) geometry optimisations were performed. The calculations converged to a BS(0,0) solution (S=0) in all cases. No spin density on the ligand or the iron atom was determined via Löwdin population analysis. Additionally, the overlap integrals (S[illegible] of the unrestricted corresponding orbitals (UCOs) with values of unity or close to unity (for the highest occupied orbitals) provide further evidence for a BS(0,0) ground state. Because of these results, all subsequent calculations for **2**, **7**, **8** and **11** were performed for the complexes as closed shell singlets.

Nuclear Independent Chemical Shift (NICS)

Chemical shifts for **7**, **8**, pyrazine and $P^{Pz}DIFe(CO)_2$ were computed as described by von Ragué Schleyer[32] at the B3LYP/TZVP level of theory from the optimised geometries, using the EPR/NMR module in Orca, with gauge-independent atomic orbital (GIAO) method. Both $NICS(1)_{iso}$ and $NICS(1)_{\pi yy}$ values are given for comparison. Because of small differences (<1 ppm) between the two sides of the 6-member hereocyclic core, both NICS(1) and NICS(-1) were calculated and averaged.

Typical Input:

```
! TightSCF NMR B3LYP def2-TZVP def2/JK def2/J RIJCOSX Pal16 Grid6 GridX6
* xyz 0 1
coordinates from optimised structure
%eprnmr
   GIAO_2el = giao_2el_same_as_scf
   GIAO_1el = giao_1el_analytic
end
```

[31] (a) A. P. Ginsberg *J. Am. Chem. Soc.* **1980**, *102*, 111. (b) L. Noodleman, C. Y. Peng, D. A. Case, J. M. Mouesca *Coord. Chem. Rev.* **1995**, *144*, 199.

[32] (a) P. von R. Schleyer, C. Maerker, A. Dransfeld, H. Jiao, N. J. R. van E. Hommes, *J. Am. Chem. Soc.* **1996**, *118*, 6317-6318. (b) Z. Chen, C. S. Wannere, C. Corminboeuf, R. Puchta, P. von R. Schleyer, *Chem. Rev.* **2005**, *105*, 3842-3888. (c) H. Fallah-Bagher-Shaidaei, C. S. Wannere, C. Corminboeuf, R. Puchta, P. von R. Schleyer, *Org. Lett.* **2006**, *8*, 863-866.

Comparison between experimental and calculated metric data

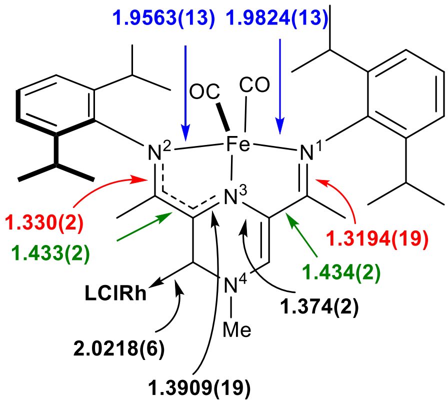

L = COD

complex 7: Xray metric data

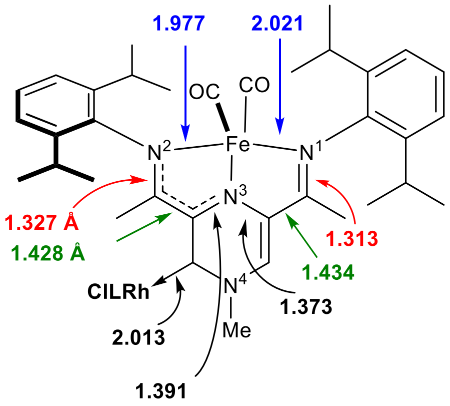

L = COD

B3LYP/ def-TZVP(-f)

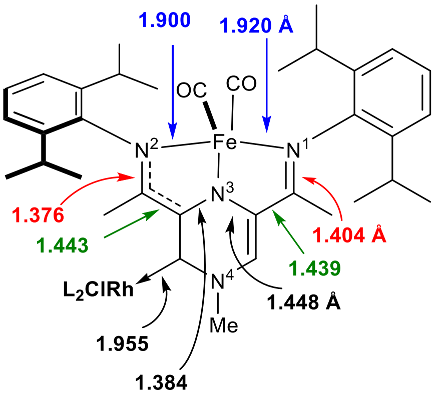

L = CO

complex 8: Xray metric data

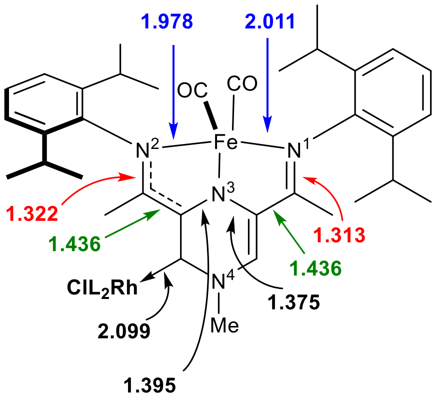

L = CO

B3LYP/ def-TZVP(-f)

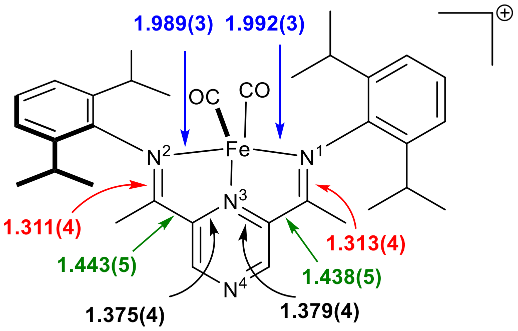

complex 10: Xray metric data

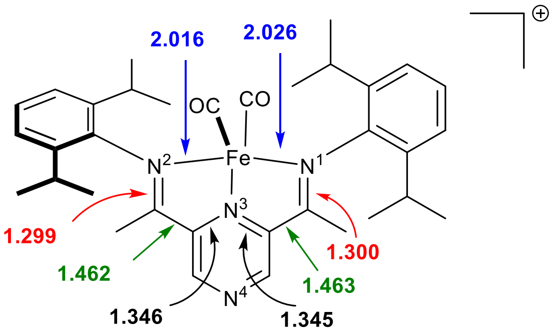

B3LYP, def2-TZVP(-f)

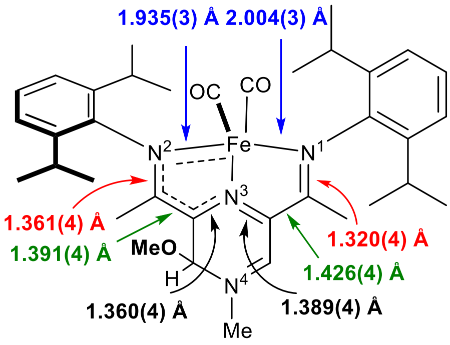

complex 11: Xray metric data

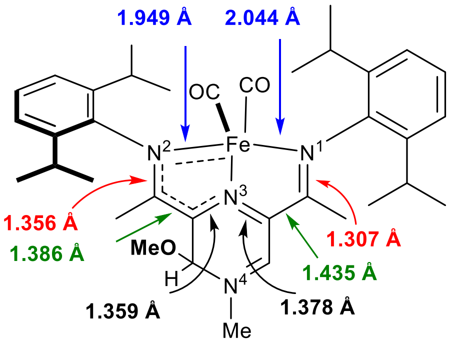

B3LYP/ def-TZVP(-f)

Calculated metric data for complexes which were not crystallised

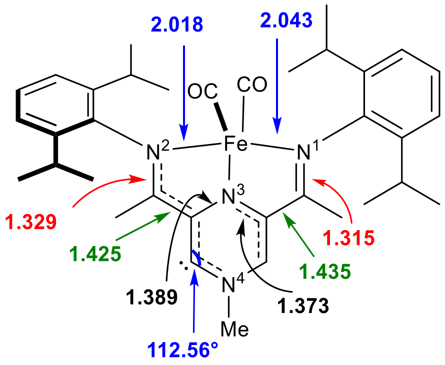

Compound 2_B3LYP/ def-TZVP(-f)

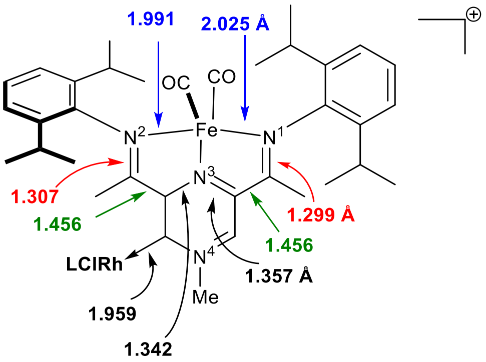

L = COD

B3LYP/ def-TZVP(-f)

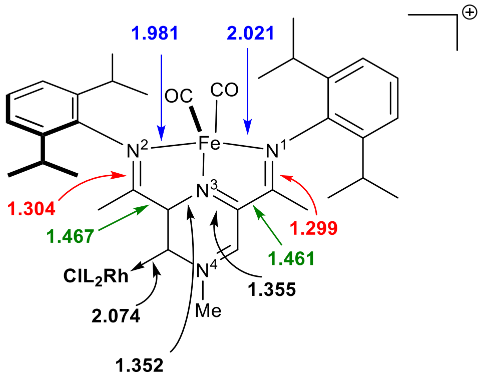

L = CO

Compound 9_B3LYP/ def-TZVP(-f)

Molecular orbital diagrams

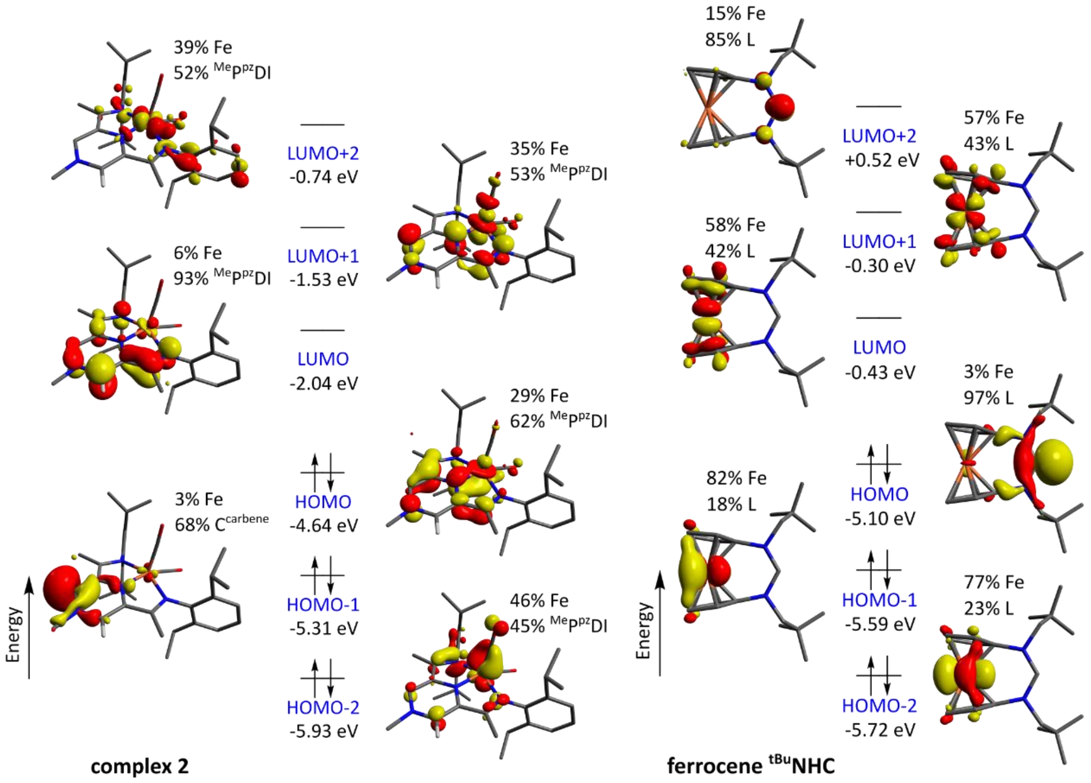

Figure S11. Qualitative molecular orbital diagrams of **2** and the Np-diamino[3]ferrocenophane reported by Siemeling. Canonical molecular orbitals are displayed.

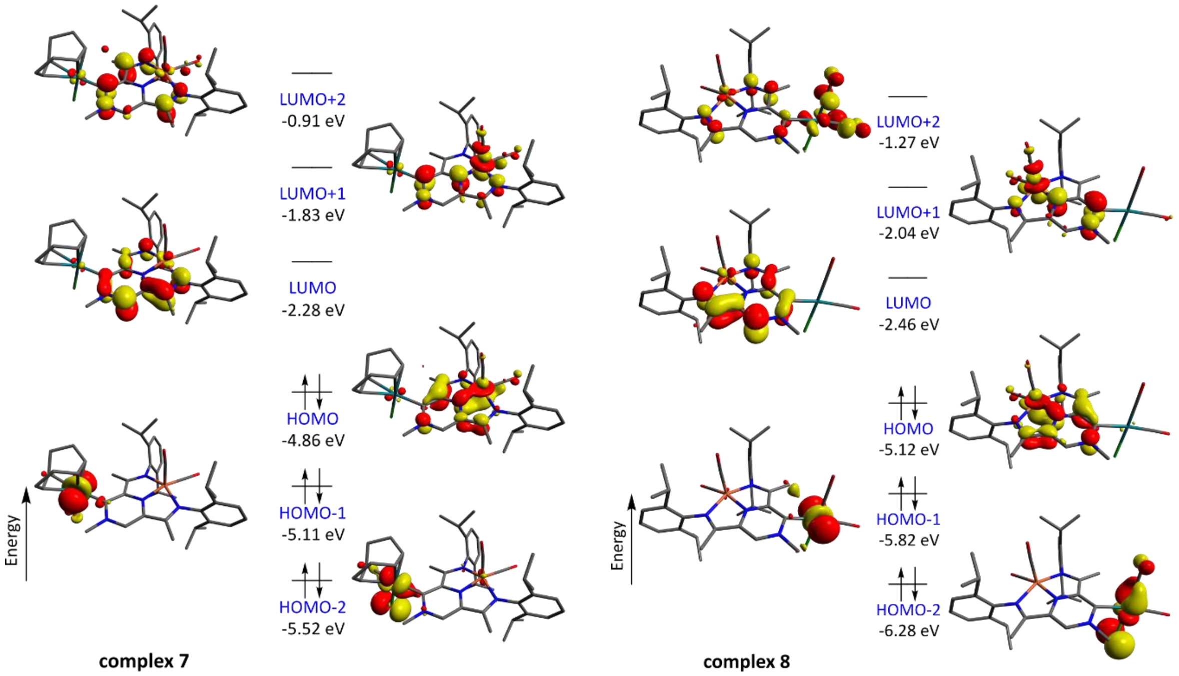

Figure S12. Qualitative molecular orbital diagrams of **7** and **8**. Canonical molecular orbitals are displayed.

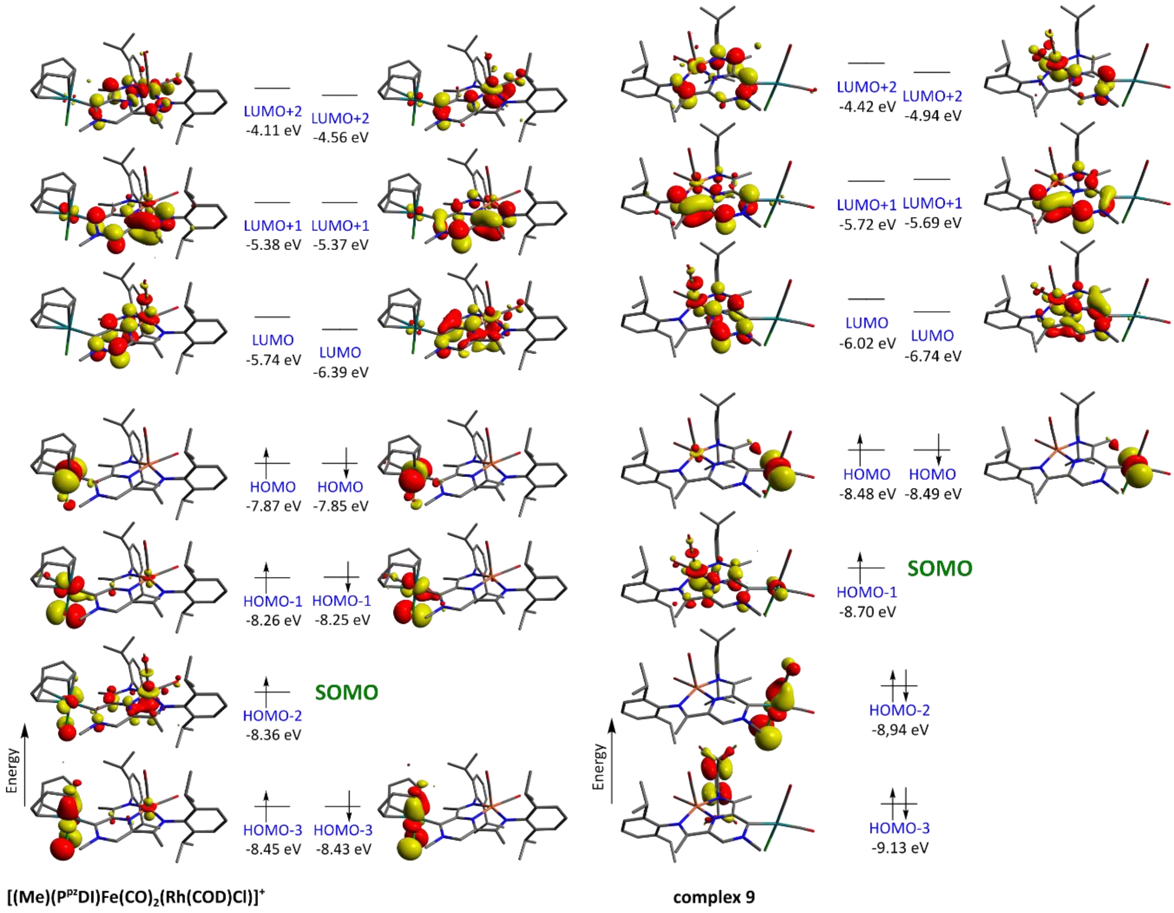

Figure S13. Qualitative molecular orbital diagrams of **[MeP^{Pz}DIFe(CO)$_2$(RhCODCl)]$^+$** and **9**. Canonical molecular orbitals are displayed.

SOMO plots

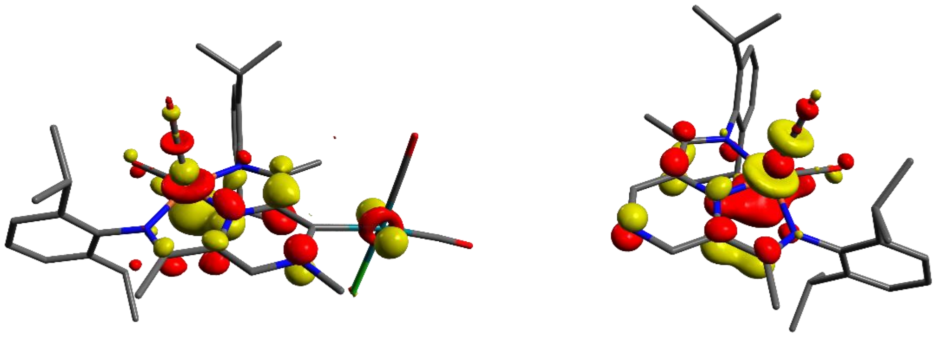

Figure S14. Singly occupied molecular orbitals of **9** (HOMO-1) (left) and **10** (HOMO) (right). Canonical molecular orbitals are displayed.

NBO Analysis

Additional details for 2:

The carbene lone pair (42.06% s character) is delocalised in the σ* orbitals of N^3-C^{18} (14 kcal mol^{-1}) and N^4-C^1 (10 kcal mol^{-1}). The high s character of the lone pair is also mirrored in the acute N4-C17-C18 angle (112.56°) significantly compressed from the ideal 120° (for a perfectly sp^2 hybridised carbon) or 119.03(18) in **1·[I]**. The carbene empty p orbital is in bonding combination with the (partially) filled lone pairs on N^4 and C^{18} (occupancy 1.08). Therefore N^4-C^{17}-C^{18} for a 3c/4e bond (weight N^4-C^{17} : C^{18} to N^4 : C^{17}-C^{18} 32% to 68%). The partially filled orbital on C^{18} is also delocalised into the π* of the N^2-C^{19} imine bond. The strong delocalisation over N^4-C^{17}-C^{18}-C^{19}-N^2 is reflected also in the second-order donor-acceptor interactions $p(C^{18}) \rightarrow \pi^*(N^4\text{-}C^{17})$ ($E^{(2)}$ = 156 kcal mol^{-1}) and $p(C^{18}) \rightarrow \pi^*(N^2\text{-}C^{19})$ ($E^{(2)}$ = 175 kcal mol^{-1}). The π bond between N^4-C^{17} in the "best" Lewis structure is also strongly polarised (83% contribution from N). All in all, the bonding picture suggests strong stabilisation of the carbene empty p orbital over the entire N^4-C^{17}-C^{18}-C^{19}-N^2 system. The second half of the pyrazine ring is described as a partially filled p orbital on N^3 (occupancy 1.42) which is delocalised into the π* of C^1-C^2 (occupancy 0.29, $E^{(2)}$ = 22 kcal mol^{-1}). The π-bond of C^8-C^{10} is also in conjugation with the N^1-C^3 imine (E = 24 kcal mol^{-1}).

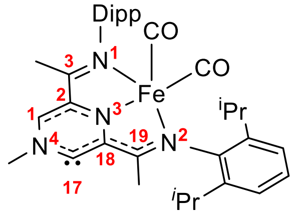

2

Additional plots:

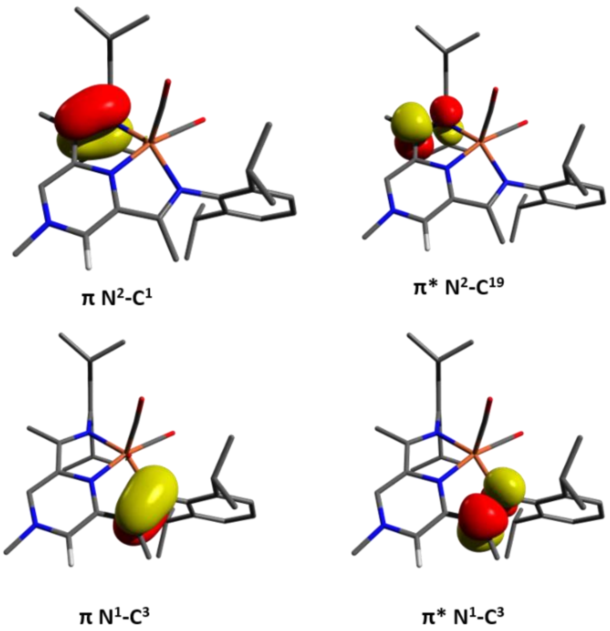

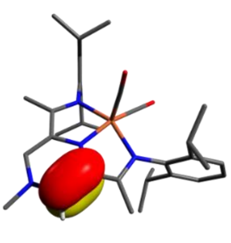

π C^1-C^2

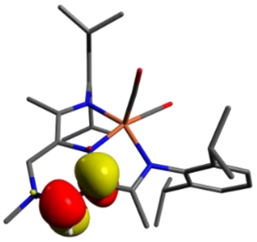

π* C^1-C^2

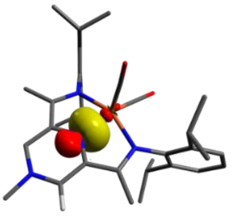

sp^2-lone pair at N^3
engaged in N-Fe coordination

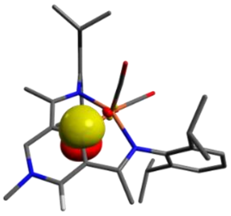

p-lone pair at N^3
engaged in hyperconjugation with
π*-C^1-C^2

Additional details for 8:

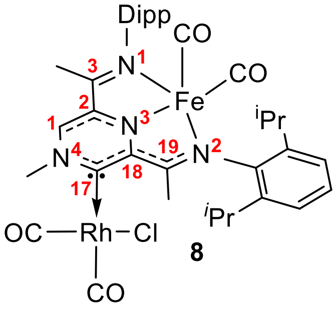

8

The bonding picture in the carbene fragment of **8** is very similar to **2**. The core atoms N^4-C^{17}-C^8 form a 3c/4e bond bond (weight N^4-C^{17} : C^{18} to N^4 : C^{17}-C^{18} 32% to 68%), consisting of a strongly polarised π orbital formed between N^4-C^{17} (80%N composition) and partially occupied p AO at C^{18} (occupancy 1.08). The partially occupied p AO at C^{18} is strongly delocalised into the ring N^4-C^{17} π* MO ($E^{(2)}$ = 264 kcal mol^{-1}) and into the adjacent imine N^{19}-C^2 π* MO ($E^{(2)}$ = 139 kcal mol^{-1}). The other half of the ring consist of a lone pair at N^3 (occupancy 1.42), delocalised in the C^1-C^2 π* molecular orbital ($E^{(2)}$ = 20 kcal mol^{-1}). The C^1-C^2 π molecular orbital is delocalised into the N^1-C^3 imine π* molecular orbital ($E^{(2)}$ = 23 kcal mol^{-1}). The carbene lone pair (30.3 % s character) is involved in a 3c/4e bond with Rh-CO (trans) bond (weight C^{17}-Rh : CO to C^{17} : Rh-CO 51% to 49%). The donor-acceptor interaction between the C^{17} sp^2 lone pair and the σ* of Rh-CO is strong ($E^{(2)}$ = 115 kcal mol^{-1}).

NBO plots:

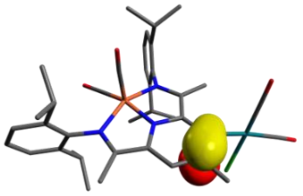

π N^{4}-C^{17}

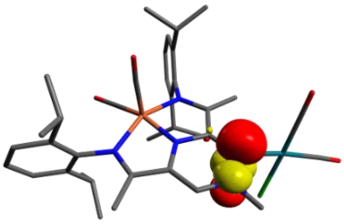

π^* N^{4}-C^{17}

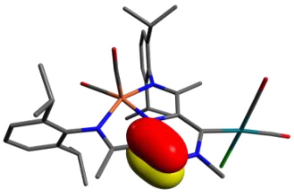

π C^{1}-C^{2}

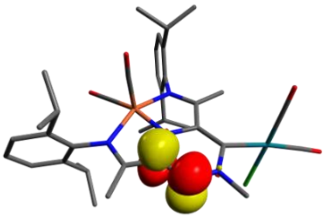

π^* C^{1}-C^{2}

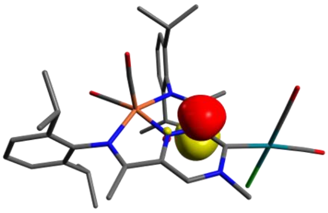

p-lone pair at C^{18}
engaged in hyperconjugation with
π^*-N^{4}-C^{17} and π^*-N^{2}-C^{19}

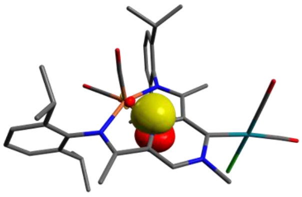

p-lone pair at N^{3}
engaged in hyperconjugation with
π^*-C^{1}-C^{2}

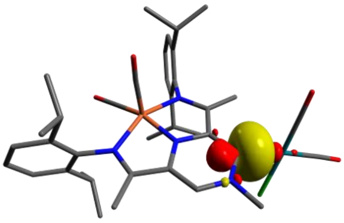

sp^{2}-lone pair at C^{17}
engaged in hyperconjugation with
σ^*-Rh-CO

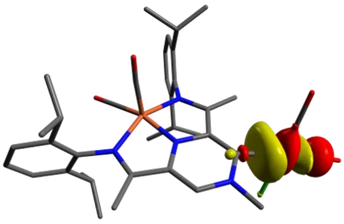

σ^*-Rh-CO

Additional details for 11:

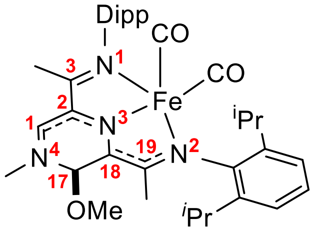

11

The C^{18}-C^{19}-N^2 form a 3c/4e bond (weight C^{18}-C^{19} : N^2 to C^{18} : C^{19}-N^2 60% to 40 %), which consists of the N^2-C^{19} π-molecular orbital and a partially filled p-type orbital at C^{18} (occupancy 1.15). This orbital is strongly delocalised into the π* MO of N^2-C^{19} ($E^{(2)}$ = 227 kcal mol^{-1}) and more weakly in the σ* of the C^{17}-OMe ($E^{(2)}$ = 28 kcal mol^{-1}). A partially filled lone pair at N3 interacts with the π* MO of C^1-C^2 ($E^{(2)}$ = 19 kcal mol^{-1}). The latter orbital also interacts with a p-type lone pair at N^4 ($E^{(2)}$ = 23 kcal mol^{-1}) suggesting conjugation between N^4-C^1-C^2-N^3. This conjugation is absent on the other side of the heterocyclic ring (i.e. between N^4-C^{17}-C^{18}). The π MO of C^1-C^2 interacts with the π* of the adjacent imine N^1-C^3 ($E^{(2)}$ = 24 kcal mol^{-1}).

NBO plots:

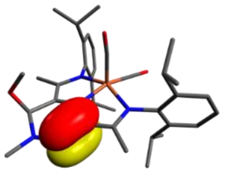
π C^1-C^2

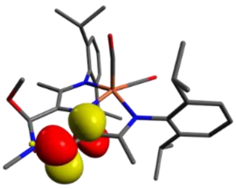
π* C^1-C^2

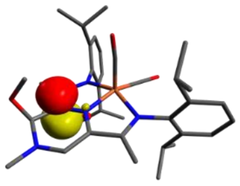
**p-lone pair at C^{18}
engaged in hyperconjugation with
π*-N^2-C^{19} and σ* of C^{17}-OMe**

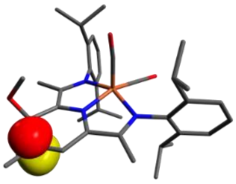
**p-lone pair at N^4
engaged in hyperconjugation with
π*-C^1-C^2**

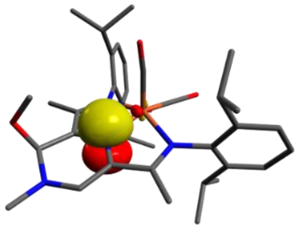
**p-lone pair at N^3
engaged in hyperconjugation with
π*-C^1-C^2**

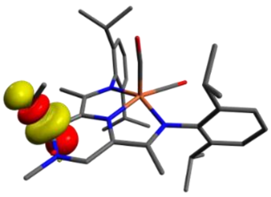
and σ* of C^{17}-OMe

IR Spectra

Table S6. Comparison between solution* and calculated IR Stretching frequencies (cm^{-1}) for selected compounds. Scaling of 0.965 for the calculated frequencies[29] was applied

No.	Compound	ν_{CO} (cm^{-1})*[soln]*	ν_{CO} (cm^{-1}) (calcd)
**8**	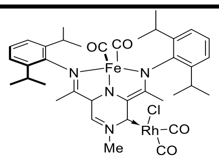	2074, 1995, 1931	2055 (RhCO), 2002(FeCO), 1980(RhCO), 1999 (FeCO)
**9**	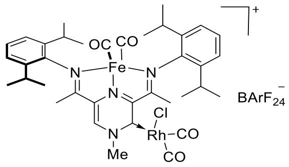	2081, 2007, 1948	2087 (RhCO), 2063 (FeCO), 2026 (FeCO), 2005 (RhCO)
**11**	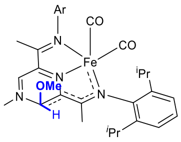	1974, 1906 (pentane)	1982, 1948

* Recorded in CH_2Cl_2 solutions (KBr plates, liquid cell), unless otherwise stated.

Cartesian Coordinates for Optimised Structures

Table S7. Optimised coordinates for **2-singlet.** Final single point energy: -2992.899012145072 Ha

! RKS B3LYP OPT D3BJ def2-TZVP def2/J RIJCOSX Grid4 FinalGrid5 VeryTightSCF PAL16

Fe	0,05587	4,31305	2,49224
O	1,86998	6,03569	0,90038
O	-1,37329	3,15617	0,23497
N	-1,21837	5,80221	3,07042
N	1,36653	2,82286	2,85712
N	-0,62606	3,60714	4,03436
N	-1,53205	2,60238	6,40422
C	-2,07704	3,74541	5,89927
H	-2,83503	4,23993	6,49027
C	-1,63063	4,2541	4,71086
C	-1,96898	5,51308	4,11071
C	-3,06449	6,38415	4,6497
H	-3,18152	6,25288	5,72491
H	-4,01807	6,12276	4,18183
H	-2,87532	7,43616	4,44817
C	-1,29541	7,12562	2,50929
C	-0,4063	8,09984	3,02167
C	0,59592	7,77551	4,12732
H	0,67492	6,6895	4,17647
C	2,00625	8,31542	3,84829
H	2,348	8,06093	2,84467
H	2,70996	7,88418	4,56189
H	2,05617	9,40113	3,96448
C	0,1306	8,2655	5,5107
H	0,0939	9,35744	5,54867
H	0,83072	7,931	6,2798
H	-0,85714	7,88547	5,77355
C	-0,48075	9,39114	2,5044
H	0,18446	10,15507	2,88606
C	-1,38884	9,719	1,5076
H	-1,43078	10,72876	1,11821
C	-2,23886	8,74755	1,00754
H	-2,94089	9,00998	0,22599
C	-2,22254	7,44035	1,49675
C	-3,22284	6,43793	0,93263
H	-3,10606	5,50079	1,47762
C	-2,97232	6,1433	-0,55657
H	-3,17183	7,02663	-1,16765
H	-3,63474	5,34655	-0,90136
H	-1,94564	5,8288	-0,74225
C	-4,67517	6,91574	1,12397
H	-4,88738	7,1828	2,16106

H	-5,37424	6,13283	0,82104
H	-4,88555	7,79455	0,51097
C	-0,54507	1,85909	5,77801
C	-0,12959	2,42794	4,57422
C	1,00085	1,98933	3,82493
C	1,68793	0,69487	4,14148
H	1,41268	-0,05838	3,39716
H	1,35086	0,34679	5,11565
H	2,77326	0,78195	4,12848
C	2,663	2,61936	2,26716
C	3,77106	3,20875	2,92204
C	3,5991	4,12181	4,13309
H	2,5679	4,4774	4,11615
C	4,50174	5,36077	4,06876
H	5,55422	5,10569	4,21386
H	4,22858	6,05798	4,86331
H	4,40344	5,88012	3,1143
C	3,80451	3,39848	5,47606
H	3,1214	2,56036	5,60205
H	3,62596	4,09211	6,30194
H	4,82592	3,01868	5,56876
C	5,04813	2,93548	2,43564
H	5,90937	3,35515	2,93944
C	5,23869	2,13788	1,3166
H	6,23833	1,93011	0,95417
C	4,14025	1,61479	0,65667
H	4,29347	1,00811	-0,2269
C	2,83952	1,83352	1,11433
C	1,68139	1,20339	0,35455
H	0,77336	1,37004	0,93289
C	1,49366	1,86405	-1,02314
H	1,34776	2,94124	-0,93842
H	0,6238	1,44494	-1,5336
H	2,36791	1,6964	-1,65635
C	1,85175	-0,31574	0,19385
H	2,67334	-0,55818	-0,48339
H	0,94535	-0,75667	-0,22737
H	2,05944	-0,80165	1,14885
C	-1,93094	2,20488	7,75983
H	-3,00124	2,35932	7,90951
H	-1,38092	2,79181	8,4998
H	-1,67713	1,15699	7,88399
C	1,18136	5,36762	1,52387
C	-0,79173	3,64926	1,09274

Table S8. Optimised for **2 – triplet** Final single point energy: -2992.839608311824 Ha

! UKS B3LYP OPT D3BJ def2-TZVP def2/J RIJCOSX Grid5 FinalGrid7 VeryTightSCF PAL16

**Fe**	0,12801	4,40776	2,4613
**O**	1,83889	6,0766	0,69886
**O**	-1,58033	3,02431	0,39124
**N**	-1,26558	5,86696	3,10658
**N**	1,47325	2,85134	2,87716
**N**	-0,73904	3,52704	3,9483
**N**	-1,62059	2,48719	6,30225
**C**	-2,17409	3,66197	5,81463
**H**	-2,9282	4,14892	6,41413
**C**	-1,70541	4,17378	4,63699
**C**	-2,03757	5,48703	4,07653
**C**	-3,1819	6,27685	4,63988
**H**	-3,09104	6,37935	5,72255
**H**	-4,11796	5,7489	4,43917
**H**	-3,24109	7,26834	4,20048
**C**	-1,34126	7,19896	2,57423
**C**	-0,40898	8,13619	3,07442
**C**	0,58609	7,75259	4,1662
**H**	0,66297	6,66453	4,15324
**C**	2,00169	8,29667	3,93266
**H**	2,35861	8,07521	2,92606
**H**	2,69125	7,83609	4,64224
**H**	2,05537	9,3769	4,08919
**C**	0,10137	8,15795	5,57018
**H**	0,08131	9,24577	5,6753
**H**	0,7772	7,76209	6,33154
**H**	-0,90004	7,78151	5,78337
**C**	-0,43223	9,42598	2,55282
**H**	0,26463	10,16449	2,92737
**C**	-1,32772	9,78229	1,55364
**H**	-1,32492	10,78836	1,15303
**C**	-2,21833	8,84343	1,06246
**H**	-2,90507	9,12733	0,2749
**C**	-2,25593	7,5395	1,56003
**C**	-3,27814	6,56379	0,98674
**H**	-3,20554	5,62998	1,54482
**C**	-3,00237	6,23689	-0,49209
**H**	-3,15901	7,11536	-1,12241
**H**	-3,68181	5,45551	-0,83916
**H**	-1,98192	5,88686	-0,64663
**C**	-4,71746	7,09099	1,13867
**H**	-4,95037	7,36145	2,17054
**H**	-5,43307	6,33257	0,81272
**H**	-4,88134	7,97733	0,52262

C	-0,5655	1,82812	5,72105
C	-0,14265	2,41969	4,522
C	1,06742	2,04162	3,8354
C	1,78024	0,78065	4,22724
H	1,5244	-0,01202	3,5175
H	1,44173	0,46908	5,2128
H	2,86231	0,89645	4,20971
C	2,75509	2,61462	2,27778
C	3,88026	3,26121	2,83763
C	3,73107	4,26447	3,97684
H	2,73782	4,70847	3,87109
C	4,75038	5,40928	3,90321
H	5,76091	5,06568	4,13578
H	4,49696	6,17699	4,63697
H	4,76635	5,8757	2,91673
C	3,79266	3,61255	5,36877
H	3,01329	2,867	5,51363
H	3,66845	4,37346	6,14404
H	4,75874	3,1256	5,52705
C	5,14055	2,96611	2,32139
H	6,01579	3,43049	2,75622
C	5,29566	2,08752	1,2589
H	6,2826	1,86082	0,87354
C	4,17793	1,51356	0,67886
H	4,3027	0,85118	-0,16846
C	2,89431	1,75888	1,16884
C	1,70909	1,10301	0,47406
H	0,82332	1,28118	1,08273
C	1,46409	1,73387	-0,90955
H	1,30872	2,81096	-0,83996
H	0,58125	1,29476	-1,37974
H	2,31698	1,56246	-1,57049
C	1,88058	-0,41858	0,33607
H	2,67894	-0,67083	-0,3649
H	0,96268	-0,87069	-0,04695
H	2,12398	-0,88715	1,29154
C	-2,09602	2,02358	7,60814
H	-3,18599	1,94942	7,62239
H	-1,77627	2,71455	8,39348
H	-1,65268	1,0499	7,78816
C	1,17864	5,42396	1,37116
C	-0,91137	3,61667	1,1078

Table S9. Optimised coordinates for Np-diaminocarbene[3]ferrocenophane**.** Final single point energy: -2191.120442681731 Ha

! RKS B3LYP OPT D3BJ def2-TZVP def2/J RIJCOSX Grid5 FinalGrid5 VeryTightSCF PAL16

C	0,05931	9,25854	5,99069
C	-0,21904	6,85432	5,28716
C	-1,48563	6,20503	5,12609
H	-2,40565	6,69008	4,84561
C	-1,32548	4,83422	5,46693
H	-2,10603	4,09049	5,46936
C	0,04009	4,6154	5,80641
H	0,47386	3,67744	6,11425
C	0,73174	5,85109	5,67375
H	1,77564	6,02891	5,87782
C	-0,75533	7,87121	7,94179
C	-2,06594	7,30316	8,06675
H	-2,96946	7,71223	7,64827
C	-1,95061	6,07614	8,7751
H	-2,76067	5,40214	9,00362
C	-0,5835	5,88972	9,1239
H	-0,17643	5,04843	9,66116
C	0,15254	7,00396	8,63694
H	1,21609	7,16003	8,72558
C	0,68656	8,53603	3,77031
H	0,94379	7,56523	3,34246
H	1,61887	9,07566	3,94236
C	-0,15161	9,32162	2,72177
C	-0,10552	10,83034	3,00099
H	0,91799	11,20555	2,91444
H	-0,46113	11,0621	4,00401
H	-0,71769	11,36993	2,27406
C	0,49624	9,06836	1,34853
H	0,40655	8,01971	1,05204
H	1,55772	9,33354	1,35405
H	0,01222	9,67644	0,58114
C	-1,60957	8,84768	2,67375
H	-2,11804	9,03328	3,62081
H	-1,68271	7,78112	2,44566
H	-2,15041	9,39049	1,89527
C	-0,25216	10,24938	8,16169
H	0,6551	10,78169	7,87636
H	-0,10211	9,84946	9,16763
C	-1,41923	11,27181	8,20599
C	-1,41432	12,15323	6,94971
H	-1,59443	11,56094	6,05312
H	-0,45263	12,65539	6,82269
H	-2,19117	12,91981	7,01936

**C**	-1,17832	12,14234	9,45041
**H**	-0,17099	12,56788	9,45728
**H**	-1,3041	11,55342	10,36348
**H**	-1,8881	12,97168	9,48494
**C**	-2,79242	10,60245	8,34163
**H**	-3,56218	11,36538	8,48451
**H**	-2,83431	9,92233	9,19505
**H**	-3,04907	10,04147	7,44205
**N**	0,07847	8,24683	5,09667
**N**	-0,37966	9,08089	7,25858
**Fe**	-0,769	6,10364	7,04212

Table S10. Optimised coordinates for **7.** Final single point energy: -3875.953544763079 Ha

! RKS B3LYP D3BJ RIJCOSX def2-TZVP(-f) def2/J PAL16 VeryTightSCF Grid5 Finalgrid5 Opt

**Rh**	17,47887	3,46937	8,79998
**Fe**	11,95015	2,07409	7,73331
**Cl**	17,64376	3,58425	11,19462
**O**	9,05781	2,7443	7,68707
**O**	12,56778	2,36512	4,9139
**N**	12,61373	3,81586	8,3923
**N**	11,75803	0,06336	7,78344
**N**	13,57959	1,60254	8,40825
**N**	16,13984	0,90685	9,03255
**C**	15,86585	2,27099	8,92582
**C**	14,52354	2,57908	8,70838
**C**	13,89204	3,85968	8,74601
**C**	14,57075	5,10205	9,22449
**H**	15,09226	5,59681	8,40536
**H**	15,31433	4,86557	9,98378
**H**	13,84276	5,80448	9,62528
**C**	11,79922	4,99202	8,45007
**C**	11,81298	5,90778	7,38492
**C**	12,70581	5,72909	6,16992
**H**	13,26416	4,80303	6,29624
**C**	11,87897	5,60784	4,88359
**H**	11,15386	4,7969	4,9508
**H**	12,53031	5,41341	4,02956
**H**	11,33057	6,53078	4,68498
**C**	13,7248	6,86793	6,03468
**H**	13,22839	7,81121	5,79751
**H**	14,43018	6,65233	5,22929
**H**	14,29208	7,01049	6,9536
**C**	10,96949	7,01497	7,45548
**H**	10,96471	7,73141	6,64424
**C**	10,13643	7,21653	8,54338

H	9,49202	8,08524	8,58221
C	10,13434	6,3016	9,58393
H	9,48486	6,4651	10,43361
C	10,95226	5,17473	9,55875
C	10,94121	4,22046	10,74145
H	11,51416	3,33749	10,45934
C	9,52331	3,76228	11,10902
H	8,92359	4,58972	11,49244
H	9,56391	3,00407	11,89301
H	9,00328	3,33806	10,2519
C	11,62427	4,85993	11,95874
H	12,65117	5,15196	11,73557
H	11,64221	4,16056	12,79715
H	11,08349	5,75338	12,27776
C	15,21673	-0,0564	8,7752
H	15,5448	-1,08102	8,85652
C	13,94649	0,27913	8,4149
C	12,86167	-0,59067	8,06455
C	13,00549	-2,07935	8,00799
H	12,04309	-2,57116	8,12225
H	13,67865	-2,44109	8,78453
H	13,4184	-2,38085	7,0416
C	10,54497	-0,66064	7,54739
C	10,25516	-1,18282	6,27985
C	11,15871	-0,95984	5,08263
H	12,06338	-0,46111	5,42898
C	10,47231	-0,03302	4,07014
H	9,60539	-0,52582	3,62456
H	11,15929	0,24	3,26757
H	10,12616	0,8835	4,54781
C	11,57725	-2,27687	4,41399
H	12,04812	-2,9563	5,12608
H	12,28835	-2,08407	3,60815
H	10,71974	-2,79242	3,97829
C	9,06772	-1,89673	6,11872
H	8,82125	-2,30531	5,14723
C	8,19965	-2,09105	7,17807
H	7,28801	-2,65688	7,03652
C	8,49254	-1,54517	8,41997
H	7,80047	-1,69136	9,23693
C	9,65588	-0,81035	8,62896
C	9,96632	-0,20542	9,98873
H	10,54765	0,70047	9,80905
C	8,71307	0,1977	10,76676
H	8,06063	0,83457	10,16851
H	8,99895	0,74947	11,66258
H	8,13968	-0,67278	11,09161
C	10,84063	-1,13594	10,83976

H	10,30074	-2,05332	11,08563
H	11,11978	-0,64053	11,77249
H	11,75861	-1,41496	10,32544
C	17,49806	0,477	9,39637
H	18,16671	0,59333	8,54469
H	17,46699	-0,56791	9,70364
H	17,85406	1,09449	10,21681
C	10,17182	2,49036	7,72429
C	12,24421	2,26418	6,01071
C	17,68311	2,921	6,73225
C	16,95305	4,1194	6,85173
H	15,87674	4,03306	6,73648
C	17,4727	5,51819	6,61512
H	17,41274	5,77875	5,54963
H	16,80168	6,20911	7,13024
C	18,90079	5,74212	7,14348
H	19,63291	5,40383	6,41119
H	19,07203	6,81309	7,26837
C	19,12492	5,04103	8,46322
C	19,68000	3,79137	8,58567
C	20,14126	2,91932	7,44125
H	21,11133	3,27017	7,06667
H	20,31735	1,91883	7,84183
C	19,13244	2,82176	6,27891
H	19,27893	1,86837	5,76702
H	19,33758	3,58897	5,53112
H	17,09344	2,03109	6,5355
H	20,02311	3,49139	9,56875
H	19,03521	5,63772	9,36276

Table S11. Optimised coordinates for **8**. Final single point energy: -3790.655631141529 Ha

! RKS B3LYP D3BJ RIJCOSX def2-TZVP(-f) def2/J PAL16 VeryTightSCF Grid5 Finalgrid7 Opt

Rh	-0,94671	2,24954	6,69012
Fe	2,63228	0,76442	2,19827
Cl	0,47911	0,86637	8,02298
O	5,45357	1,06688	1,33242
O	1,23647	1,59267	-0,21425
O	-1,62108	3,71993	9,28378
O	-2,692	3,91116	4,90455
N	2,5076	-1,22334	1,91694
N	2,52522	2,44502	3,23495
N	1,33227	0,3528	3,40796
N	-0,7909	-0,2237	5,01613
C	-0,25655	-1,19081	4,22067
H	-0,70837	-2,16883	4,27271

C	0,80127	-0,91542	3,41188
C	1,51811	-1,81126	2,54835
C	1,15803	-3,25661	2,40475
H	0,37512	-3,38067	1,65209
H	0,78455	-3,6636	3,34381
H	2,01561	-3,84327	2,08582
C	3,40646	-2,01334	1,12478
C	3,1029	-2,32414	-0,20782
C	1,88402	-1,76785	-0,9198
H	1,30124	-1,19599	-0,19939
C	2,32522	-0,81022	-2,03555
H	2,98694	-0,03555	-1,64918
H	1,46155	-0,32363	-2,49205
H	2,86458	-1,35179	-2,81525
C	0,97356	-2,8659	-1,48389
H	1,49089	-3,46003	-2,2395
H	0,09588	-2,42108	-1,95725
H	0,63108	-3,54761	-0,70398
C	3,99322	-3,13385	-0,91255
H	3,77649	-3,38882	-1,94165
C	5,14843	-3,61361	-0,32028
H	5,82525	-4,24539	-0,88094
C	5,44918	-3,26237	0,98654
H	6,36576	-3,62226	1,43272
C	4,5988	-2,44982	1,73163
C	4,95193	-2,07469	3,16116
H	4,39799	-1,16861	3,40775
C	6,44162	-1,76286	3,33401
H	7,05927	-2,65332	3,20418
H	6,62639	-1,38137	4,33872
H	6,77498	-1,01148	2,61797
C	4,50909	-3,15697	4,15485
H	3,43881	-3,35389	4,08738
H	4,72893	-2,83892	5,17618
H	5,03621	-4,09483	3,96544
C	-0,27067	1,05732	5,10055
C	0,78522	1,32931	4,24131
C	1,54167	2,5415	4,11282
C	1,27779	3,79483	4,88132
H	2,1469	4,44618	4,87245
H	0,99726	3,571	5,90968
H	0,44465	4,33835	4,42832
C	3,52983	3,46623	3,16927
C	3,46224	4,47519	2,19813
C	2,30929	4,57434	1,21547
H	1,6243	3,75536	1,42504
C	2,79227	4,42875	-0,23293
H	3,44953	5,25556	-0,50932

H	1,94437	4,42753	-0,92005
H	3,34915	3,50272	-0,37731
C	1,51909	5,87898	1,38305
H	1,17671	6,01192	2,41046
H	0,64314	5,87329	0,73097
H	2,12788	6,74447	1,11348
C	4,49236	5,41356	2,1565
H	4,46024	6,20363	1,41737
C	5,55433	5,35539	3,04445
H	6,34464	6,09319	2,9935
C	5,60019	4,35133	4,00004
H	6,43038	4,31418	4,69325
C	4,59644	3,39016	4,08595
C	4,66883	2,31509	5,16069
H	3,86433	1,604	4,98114
C	5,98573	1,53063	5,10327
H	6,19058	1,16335	4,0985
H	5,93529	0,67366	5,77832
H	6,83123	2,14553	5,41835
C	4,46538	2,9009	6,56512
H	5,2466	3,62722	6,79924
H	4,50588	2,10727	7,31342
H	3,50252	3,40154	6,66134
C	-1,87654	-0,63415	5,91854
H	-2,54336	0,20881	6,07638
H	-1,45893	-0,93162	6,8797
H	-2,4225	-1,45862	5,46238
C	4,36661	0,95524	1,66555
C	1,85346	1,27755	0,69938
C	-1,39795	3,19166	8,30233
C	-2,03321	3,27196	5,59149

Table S12. Optimised coordinates for MeP[Pz]DIFe(CO)$_2$Rh(COD)Cl -3875.748622717002 Ha

! UKS B3LYP D3BJ RIJCOSX def2-TZVP(-f) def2/J PAL16 TightSCF Grid5 Finalgrid7 SlowConv Opt

Rh	17,45246	3,44303	8,71364
Fe	11,91083	2,07273	7,8251
Cl	17,5966	3,55096	11,09272
O	9,03065	2,84377	7,65677
O	12,54733	2,35445	4,96239
N	12,61544	3,81738	8,47647
N	11,73402	0,05555	7,80289
N	13,6415	1,59003	8,35926
N	16,16205	0,8759	8,96195
C	15,91428	2,2361	8,84215
C	14,53744	2,54161	8,6622
C	13,88633	3,8493	8,77857

C	14,60294	5,04636	9,29433
H	15,3508	5,3799	8,57515
H	15,15711	4,78687	10,19862
H	13,90826	5,85705	9,49254
C	11,80076	4,99969	8,55442
C	11,82487	5,92268	7,498
C	12,71055	5,751	6,27706
H	13,25376	4,81163	6,37804
C	11,87806	5,67036	4,9912
H	11,12674	4,88164	5,04793
H	12,52172	5,47035	4,13318
H	11,35541	6,60956	4,80466
C	13,75518	6,86899	6,16854
H	13,27903	7,83108	5,97285
H	14,44301	6,66586	5,34572
H	14,33754	6,96508	7,08429
C	10,98607	7,03231	7,58748
H	10,98372	7,76151	6,78849
C	10,15638	7,2203	8,68024
H	9,51996	8,09337	8,73503
C	10,14251	6,28748	9,70507
H	9,49291	6,44311	10,55519
C	10,95061	5,15473	9,66164
C	10,93148	4,17192	10,81916
H	11,43107	3,25647	10,49206
C	9,50542	3,79791	11,24184
H	8,98591	4,64719	11,68637
H	9,53098	3,00982	11,99543
H	8,9121	3,44699	10,39823
C	11,71761	4,72994	12,01509
H	12,74739	4,96961	11,7466
H	11,73431	4,00877	12,8339
H	11,24999	5,64466	12,38363
C	15,23071	-0,08135	8,74352
H	15,54352	-1,10784	8,8543
C	13,95952	0,27061	8,37968
C	12,82982	-0,59132	8,06342
C	12,97911	-2,07559	8,07199
H	13,47677	-2,40512	8,98519
H	13,59297	-2,39578	7,22702
H	12,01376	-2,56669	8,00014
C	10,51273	-0,64319	7,5105
C	10,29292	-1,20027	6,24236
C	11,29424	-1,12719	5,10191
H	12,17565	-0,58369	5,44577
C	10,70974	-0,35833	3,90769
H	9,87014	-0,89976	3,46973
H	11,46447	-0,23201	3,13042

H	10,34982	0,62835	4,20122
C	11,74794	-2,52607	4,65434
H	12,16396	-3,10542	5,4787
H	12,50896	-2,44828	3,87567
H	10,91262	-3,09238	4,2408
C	9,06997	-1,83565	6,02174
H	8,86668	-2,27034	5,05252
C	8,11305	-1,91991	7,01517
H	7,17538	-2,42317	6,82025
C	8,3513	-1,35266	8,25939
H	7,59236	-1,42402	9,02404
C	9,54459	-0,69252	8,5319
C	9,80734	-0,07661	9,8975
H	10,39117	0,83602	9,73708
C	8,53025	0,32641	10,63513
H	7,89425	0,96399	10,02013
H	8,78456	0,87164	11,54331
H	7,94964	-0,5467	10,93597
C	10,65865	-0,99818	10,78188
H	10,11097	-1,9125	11,01675
H	10,90718	-0,49759	11,7198
H	11,5915	-1,2874	10,29978
C	17,52973	0,44194	9,31489
H	18,18481	0,59508	8,46026
H	17,50555	-0,61001	9,59239
H	17,8757	1,04994	10,1472
C	10,12208	2,54491	7,72479
C	12,25497	2,26809	6,05923
C	17,7214	2,94	6,62397
C	16,94486	4,10594	6,74243
H	15,8747	3,98473	6,59344
C	17,41776	5,52544	6,53454
H	17,36296	5,78954	5,47078
H	16,71322	6,18942	7,03917
C	18,83061	5,80177	7,07999
H	19,58315	5,50798	6,35075
H	18,95121	6,87661	7,22146
C	19,08099	5,09925	8,39277
C	19,68438	3,87888	8,51338
C	20,17004	3,01547	7,37494
H	21,13716	3,38569	7,01528
H	20,36237	2,0185	7,77567
C	19,18125	2,90193	6,198
H	19,37024	1,96423	5,67314
H	19,36606	3,68808	5,46622
H	17,17068	2,02968	6,40726
H	20,01322	3,58081	9,50188
H	18,94558	5,67802	9,29871

Table S13 Optimised coordinates for **9**. Final single point energy: -3790.439425246364 Ha

! UKS B3LYP D3BJ RIJCOSX def2-TZVP(-f) def2/J PAL16 VeryTightSCF Grid5 Finalgrid5 Opt

**Rh**	-1,0842	2,29492	6,5291
**Fe**	2,75592	0,72994	2,31429
**Cl**	0,36794	0,89054	7,79767
**O**	5,53323	1,16492	1,29275
**O**	1,25513	1,53976	-0,0838
**O**	-1,74767	3,69348	9,17585
**O**	-2,84029	4,00042	4,78428
**N**	2,55497	-1,24876	1,95516
**N**	2,61356	2,41737	3,34236
**N**	1,27616	0,36614	3,3984
**N**	-0,92631	-0,15871	4,84486
**C**	-0,37202	-1,13994	4,08916
**H**	-0,85003	-2,10639	4,10879
**C**	0,74815	-0,88066	3,35103
**C**	1,51873	-1,79756	2,51468
**C**	1,11177	-3,22621	2,3763
**H**	0,24868	-3,30835	1,71087
**H**	0,82448	-3,63983	3,34384
**H**	1,91658	-3,82335	1,95821
**C**	3,43941	-2,03534	1,1357
**C**	3,07199	-2,39633	-0,16942
**C**	1,79987	-1,91685	-0,84766
**H**	1,20305	-1,36488	-0,12031
**C**	2,15312	-0,95396	-1,99161
**H**	2,80145	-0,14693	-1,65042
**H**	1,25088	-0,51279	-2,41815
**H**	2,6777	-1,48377	-2,78798
**C**	0,93671	-3,07094	-1,3752
**H**	1,46294	-3,63754	-2,14407
**H**	0,02185	-2,68114	-1,82466
**H**	0,65769	-3,76891	-0,58505
**C**	3,96617	-3,18207	-0,89628
**H**	3,71153	-3,47638	-1,9053
**C**	5,17281	-3,58631	-0,35445
**H**	5,84814	-4,20104	-0,93454
**C**	5,52618	-3,18828	0,92577
**H**	6,4784	-3,49919	1,33032
**C**	4,67793	-2,39791	1,6951
**C**	5,06245	-1,99757	3,10881
**H**	4,53785	-1,06694	3,34675
**C**	6,56018	-1,72538	3,26588
**H**	7,1484	-2,63623	3,15102
**H**	6,7641	-1,33241	4,26159
**H**	6,91612	-1,00055	2,53304

C	4,58967	-3,04197	4,12967
H	3,51529	-3,21991	4,06201
H	4,81542	-2,7071	5,14371
H	5,09537	-3,99453	3,96223
C	-0,38801	1,0962	4,98632
C	0,75608	1,32881	4,19195
C	1,58497	2,53788	4,13522
C	1,29898	3,76648	4,92084
H	2,16186	4,4253	4,93478
H	1,00268	3,50811	5,93864
H	0,45981	4,30332	4,47233
C	3,60582	3,4583	3,2569
C	3,47125	4,4751	2,30124
C	2,28034	4,56854	1,36341
H	1,608	3,74006	1,5848
C	2,71677	4,44156	-0,10244
H	3,33796	5,28787	-0,39828
H	1,84657	4,41967	-0,76009
H	3,29958	3,53556	-0,27434
C	1,47971	5,86154	1,56885
H	1,16437	5,98272	2,60615
H	0,58723	5,8532	0,94075
H	2,0706	6,73584	1,29219
C	4,48839	5,42496	2,22639
H	4,41309	6,22532	1,50243
C	5,59442	5,36237	3,05859
H	6,37289	6,10932	2,97885
C	5,70044	4,34642	3,99594
H	6,56344	4,31135	4,64687
C	4,7126	3,37255	4,11852
C	4,83919	2,2957	5,18496
H	4,04693	1,56226	5,02726
C	6,1754	1,54702	5,10077
H	6,38234	1,19649	4,08994
H	6,15965	0,68347	5,768
H	7,00692	2,18107	5,41135
C	4,64908	2,88342	6,59097
H	5,40526	3,64353	6,79352
H	4,7488	2,10125	7,34521
H	3,6698	3,34767	6,70991
C	-2,10128	-0,53091	5,66079
H	-2,76659	0,32476	5,72195
H	-1,75656	-0,78555	6,66185
H	-2,6042	-1,37433	5,19358
C	4,47976	0,9944	1,67091
C	1,88876	1,25203	0,81441
C	-1,5336	3,20811	8,17626
C	-2,18354	3,3521	5,45833

Table S14 Optimised coordinates for **10**. Final single point energy: -2953.657048124204 Ha

! UKS B3LYP D3BJ RIJCOSX def2-TZVP(-f) def2/J PAL16 TightSCF Grid4 Finalgrid5 Opt

**Fe**	12,14001	14,8002	9,56893
**O**	14,63681	16,39162	9,90512
**O**	10,46949	17,24746	9,56057
**N**	12,14226	14,52985	7,56118
**N**	11,91175	14,24252	11,4923
**N**	10,89159	13,39972	9,3949
**N**	9,03648	11,39839	9,14285
**C**	9,58289	11,9667	8,06539
**H**	9,25296	11,60122	7,10041
**C**	10,53563	12,97796	8,16833
**C**	11,28763	13,65853	7,11409
**C**	11,07292	13,29894	5,68293
**H**	11,80401	13,78129	5,04173
**H**	10,07366	13,60214	5,36245
**H**	11,14033	12,21701	5,55301
**C**	12,98005	15,27592	6,6605
**C**	12,45951	16,36539	5,94476
**C**	11,01741	16,83047	6,04638
**H**	10,49855	16,20114	6,77073
**C**	10,93546	18,2808	6,5461
**H**	11,51303	18,42878	7,45743
**H**	9,89894	18,55376	6,74986
**H**	11,31974	18,97209	5,79517
**C**	10,28818	16,69802	4,70114
**H**	10,75118	17,33456	3,94618
**H**	9,2465	17,00829	4,80114
**H**	10,30703	15,67621	4,32248
**C**	13,3319	17,06353	5,10989
**H**	12,95869	17,90632	4,54404
**C**	14,66183	16,70186	4,99345
**H**	15,31817	17,25751	4,33697
**C**	15,15502	15,62842	5,72121
**H**	16,19534	15,35419	5,61935
**C**	14,33278	14,89925	6,57439
**C**	14,8696	13,69893	7,33547
**H**	14,24386	13,56491	8,22252
**C**	16,3109	13,88507	7,81848
**H**	17,01658	13,88552	6,98697
**H**	16,59094	13,0623	8,47686
**H**	16,43695	14,81862	8,36723
**C**	14,74696	12,41606	6,49873
**H**	13,71771	12,22517	6,19094
**H**	15,1005	11,55504	7,0697
**H**	15,35185	12,49411	5,59367

C	9,4464	11,80254	10,34591
H	9,00874	11,30723	11,20405
C	10,3962	12,81032	10,4984
C	11,01724	13,32721	11,71666
C	10,64499	12,77639	13,05255
H	11,25261	13,20837	13,84097
H	10,77208	11,69168	13,05787
H	9,5934	12,97911	13,26661
C	12,63342	14,84167	12,5832
C	12,04439	15,88276	13,31434
C	10,66974	16,44015	12,99231
H	10,30404	15,94552	12,09268
C	10,74775	17,94291	12,69247
H	11,03108	18,50569	13,58266
H	9,77993	18,31482	12,3531
H	11,48532	18,15925	11,92017
C	9,65018	16,16583	14,10601
H	9,57712	15,10378	14,34164
H	8,66175	16,51673	13,80364
H	9,9249	16,68868	15,02341
C	12,78568	16,43631	14,35707
H	12,35622	17,2366	14,94446
C	14,06286	15,98616	14,64675
H	14,62146	16,43206	15,45883
C	14,63119	14,97153	13,88993
H	15,63148	14,63353	14,1228
C	13,93436	14,37941	12,8405
C	14,54447	13,23896	12,04312
H	13,99653	13,16775	11,09977
C	16,01873	13,47031	11,69541
H	16,17588	14,44275	11,22903
H	16,3626	12,70071	11,00285
H	16,65379	13,41445	12,5802
C	14,36827	11,89939	12,77677
H	14,91539	11,90614	13,72097
H	14,75438	11,07813	12,16972
H	13,32153	11,6954	13,0068
C	13,69597	15,77372	9,76511
C	11,10166	16,30206	9,57374

Table S15. Optimised coordinates for **11.** Final single point energy: -3108.865107823104 Ha

! RKS B3LYP D3BJ def2-TZVP(-f) PAL16 TightSCF Grid4 Finalgrid5 Opt

Fe	0,12553	4,32356	2,61602
O	-1,64011	0,50559	5,04958
N	-0,6173	3,59001	4,14345

**N**	1,38744	2,8811	2,9706
**N**	-1,12885	5,83187	3,19149
**N**	-1,83124	2,49099	6,34926
**C**	-0,14408	2,40189	4,60273
**C**	-0,91928	1,61556	5,60375
**H**	-0,26829	1,13021	6,33235
**C**	-2,24209	3,68426	5,86162
**H**	-3,0218	4,18627	6,41623
**C**	-1,66348	4,24208	4,75981
**C**	1,00249	2,00583	3,93165
**C**	1,72562	0,72105	4,19656
**H**	1,43917	-0,04533	3,47164
**H**	1,49028	0,33487	5,18751
**H**	2,80436	0,84982	4,1201
**C**	-1,92125	5,53284	4,1876
**C**	-3,01316	6,42401	4,69685
**H**	-3,04617	6,41992	5,78658
**H**	-3,98329	6,07711	4,33342
**H**	-2,87424	7,44655	4,35583
**C**	-2,49418	1,88707	7,49209
**H**	-3,17087	1,08843	7,18164
**H**	-3,05537	2,64786	8,03164
**H**	-1,74797	1,46079	8,16558
**C**	-2,44175	0,82497	3,91642
**H**	-1,83863	1,23368	3,10278
**H**	-3,22877	1,5431	4,16959
**H**	-2,90137	-0,10685	3,59315
**C**	2,64591	2,6966	2,32883
**C**	2,75103	1,94762	1,14571
**C**	4,00328	1,81423	0,54887
**H**	4,09673	1,24368	-0,3667
**C**	5,13054	2,39552	1,10623
**H**	6,09621	2,2774	0,63068
**C**	5,01385	3,13503	2,27315
**H**	5,89588	3,59306	2,70261
**C**	3,7814	3,30466	2,89819
**C**	1,54065	1,30591	0,49796
**H**	0,68922	1,48205	1,15212
**C**	1,70142	-0,21142	0,34489
**H**	2,51575	-0,45926	-0,33919
**H**	0,78553	-0,65147	-0,05618
**H**	1,91748	-0,6873	1,30273
**C**	1,23639	1,95939	-0,85616
**H**	1,13354	3,03992	-0,75619
**H**	0,30641	1,56766	-1,27269
**H**	2,03926	1,76509	-1,57088
**C**	3,68524	4,12186	4,17415
**H**	2,62607	4,27231	4,37968

C	4,3269	5,50428	4,01896
H	5,40666	5,43506	3,87095
H	4,1569	6,10096	4,91786
H	3,90564	6,03948	3,16842
C	4,29195	3,3717	5,36727
H	3,79879	2,41199	5,52523
H	4,18782	3,95986	6,28247
H	5,35611	3,17961	5,20972
C	-1,19071	7,1139	2,56522
C	-2,11404	7,36233	1,53978
C	-2,12574	8,62841	0,95572
H	-2,83244	8,83904	0,16319
C	-1,24787	9,61635	1,36685
H	-1,27303	10,59425	0,90282
C	-0,32438	9,34442	2,36578
H	0,36925	10,11629	2,66935
C	-0,2699	8,09522	2,97666
C	-3,0696	6,29841	1,03476
H	-2,89717	5,39149	1,61179
C	-4,53652	6,7027	1,23173
H	-4,75095	6,93851	2,27498
H	-5,19906	5,89186	0,92097
H	-4,78696	7,5831	0,63633
C	-2,7926	5,97157	-0,43863
H	-3,02743	6,82523	-1,07794
H	-3,40096	5,12577	-0,76384
H	-1,74576	5,71284	-0,59423
C	0,73882	7,81064	4,07614
H	0,90959	6,73294	4,08446
C	0,18195	8,19788	5,45364
H	-0,03218	9,26866	5,49643
H	0,90647	7,96457	6,2373
H	-0,73969	7,66198	5,67904
C	2,08938	8,48902	3,83612
H	2,48348	8,2471	2,84874
H	2,8107	8,15206	4,58143
H	2,0202	9,57553	3,92005
C	1,26833	5,3349	1,63919
C	-0,8421	3,66705	1,30931
O	1,98235	5,98231	1,01991
O	-1,49333	3,13224	0,52493
